# Programmable site-specific DNA double-strand breaks via PNA-assisted prokaryotic Argonautes

**DOI:** 10.1093/nar/gkad655

**Published:** 2023-08-10

**Authors:** Tin Marsic, Sivakrishna Rao Gundra, Qiaochu Wang, Rashid Aman, Ahmed Mahas, Magdy M Mahfouz

**Affiliations:** Laboratory for Genome Engineering and Synthetic Biology, Division of Biological Sciences, 4700 King Abdullah University of Science and Technology, Thuwal 23955-6900, Saudi Arabia; Laboratory for Genome Engineering and Synthetic Biology, Division of Biological Sciences, 4700 King Abdullah University of Science and Technology, Thuwal 23955-6900, Saudi Arabia; Laboratory for Genome Engineering and Synthetic Biology, Division of Biological Sciences, 4700 King Abdullah University of Science and Technology, Thuwal 23955-6900, Saudi Arabia; Laboratory for Genome Engineering and Synthetic Biology, Division of Biological Sciences, 4700 King Abdullah University of Science and Technology, Thuwal 23955-6900, Saudi Arabia; Laboratory for Genome Engineering and Synthetic Biology, Division of Biological Sciences, 4700 King Abdullah University of Science and Technology, Thuwal 23955-6900, Saudi Arabia; Laboratory for Genome Engineering and Synthetic Biology, Division of Biological Sciences, 4700 King Abdullah University of Science and Technology, Thuwal 23955-6900, Saudi Arabia

## Abstract

Programmable site-specific nucleases promise to unlock myriad applications in basic biology research, biotechnology and gene therapy. Gene-editing systems have revolutionized our ability to engineer genomes across diverse eukaryotic species. However, key challenges, including delivery, specificity and targeting organellar genomes, pose barriers to translational applications. Here, we use peptide nucleic acids (PNAs) to facilitate precise DNA strand invasion and unwinding, enabling prokaryotic Argonaute (pAgo) proteins to specifically bind displaced single-stranded DNA and introduce site-specific double-strand breaks (DSBs) independent of the target sequence. We named this technology PNA-assisted pAgo editing (PNP editing) and determined key parameters for designing PNP editors to efficiently generate programable site-specific DSBs. Our design allows the simultaneous use of multiple PNP editors to generate multiple site-specific DSBs, thereby informing design considerations for potential *in vitro* and *in vivo* applications, including genome editing.

## Graphical Abstract

**Graphical Abstract:**
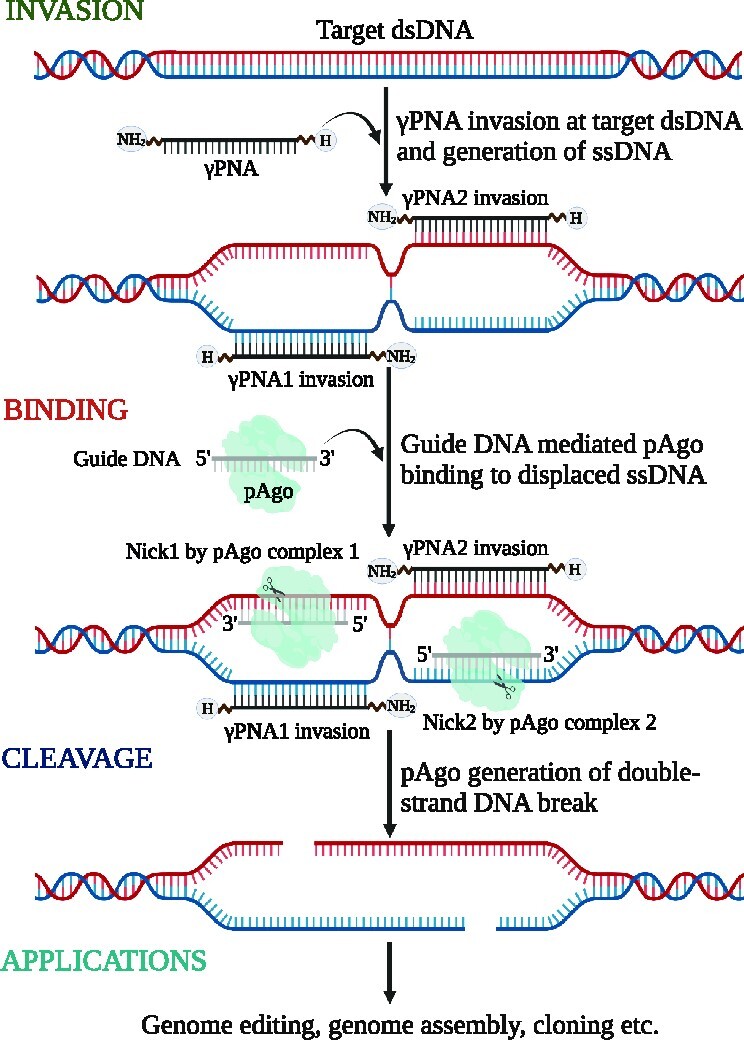

## INTRODUCTION

Members of the Argonaute protein family have been identified in all domains of life (1,2). Among prokaryotes, 32% of Archaea and 9% of Eubacteria harbor genes that encode members of the Argonaute superfamily (3). Prokaryotic Argonaute (pAgo) proteins function as an innate immune system to fend off invading genetic elements from bacteriophages and conjugative plasmids (4,5).

pAgos are classified as long pAgo, short pAgo and PIWI-RE proteins (6). The long-A pAgos have characteristic N, PAZ (PIWI/Argonaute/Zwille), MID (middle) and PIWI (P-element-induced wimpy testes) domains. The MID and PAZ domains mediate the binding of the 5′ and 3′ ends, respectively, of a guide nucleic acid molecule (7). In contrast to eukaryotic Argonautes that use a single-guide RNA, most described pAgos rely on small single-stranded guide DNA (gDNA) molecules to target DNA sequences. The PIWI domain mediates the catalytic activity of pAgo and possesses an RNase-H-like fold with a DEDX catalytic motif mediating the endonuclease activity, in most cases by slicing the target sequence between nucleotides 10 and 11 in relation to the 5′ end of the guide molecule (8).

How pAgos acquire their guides and elicit their function *in vivo* has only partially been elucidated. Many pAgos possess guide-independent activity that is suspected to be involved in guide acquisition from highly expressed or repetitive regions of target plasmids (9). Importantly, pAgos lack intrinsic helicase activity and are thus unable to unwind target double-stranded DNA (dsDNA) on their own and possess only one nuclease domain (3). Therefore, the generation of double-strand breaks (DSBs) requires the binding of two pAgo complexes, on the upper and lower strands of the target DNA sequence.

Many studies have attempted to harness and develop pAgos as programmable DNA editors for genome-editing applications. Initial attempts were limited to pAgos from hyperthermophiles such as *Pyrococcus furiosus* and *Thermus thermophilus* to generate dsDNA breaks due to temperature denaturation (10,11). With the discovery of pAgos from mesophilic organisms such as *Clostridium butyricum, Limnothrix rosea* and *Kurthia massiliensis*, their applications remained limited to regions of supercoiled DNA substrates with low GC content (12–14). Various attempts have been made to overcome this challenge, such as deploying single-stranded DNA (ssDNA)-binding proteins and combining CbAgo with a RecBC helicase from *Escherichia coli* (15). Whether these strategies can be successfully applied to eukaryotic cells *in vivo* remains to be explored. Indeed, the lack of robust pAgo activity at physiological temperatures precluded their translational applications in gene editing and biotechnology and limited their use to bacterial cells (16,17).

Peptide nucleic acids (PNAs) are synthetic oligonucleotide analogs characterized by a neutrally charged 2-aminoethyl glycine backbone replacing the canonical sugar–phosphate backbone (18). As a result of their neutral charge, PNAs can bind to complementary DNA or RNA with high affinity and specificity, resulting in hybrids that are more stable than their naturally occurring RNA or DNA counterparts (19,20). Additionally, their modified backbone renders PNAs stable and resistant to cleavage by proteases and nucleases (21,22). Owing to their remarkable properties, PNAs have been employed for diverse biotechnological applications, such as inhibition of PCR, transcription, translation and design of improved fluorescence *in situ* hybridization probes (21,23).

The potential use of PNAs for genome editing relies on the ability of PNAs to form triplexes with genomic DNA in a site-specific manner (24,25). PNAs have been deployed in gene editing to correct genetic diseases (26–28), and they have been used along with DNA oligomers to carry information for homologous recombination. PNAs were hypothesized to invade target DNA, generating distortions in DNA helix that is recognized and resolved by the nucleotide excision repair pathway, leading to stimulated recombination in the presence of externally provided DNA repair template. For example, PNAs modified at gamma position (γPNAs) and donor DNA oligomers have been delivered intravenously via poly(lactic-*co*-glycolic acid) nanoparticles into a β-thalassemia mouse model for the amelioration of this genetic disease (29). This PNA treatment led to a correction of the *β-globin* gene (30). PNAs hold much promise for gene therapy; however, the low efficiency of gene correction limits their applications in gene editing (30,31).

Here, we harnessed the features of PNA molecules and pAgos and developed PNA-assisted pAgo editors (PNP editors) to generate programmable site-specific DSBs independently of the GC content and DNA form at ambient temperatures. We harnessed the power of PNAs to mediate targeted and specific DNA invasion and the programmability of guided pAgos to bind to any ssDNA sequence of interest specifically and with high efficiency to generate targeted DSBs. Our work shows that PNA-assisted pAgo can generate single and multiple site-specific DSBs and targeted modifications for *in vitro* and *in vivo* applications. The PNP editor technology holds great promise for genome editing and clinical applications in gene therapy.

## MATERIALS AND METHODS

### Purification of Argonaute proteins used in *in vitro* experiments

#### CbAgo purification

The coding sequence of *CbAgo* codon optimized for *E. coli* (Supplementary File S1A; Supplementary Table S1) was subcloned into the His_6_-TwinStrep-SUMO expression vector and transformed into *E. coli* strain BL21. Starter cultures were prepared by growing a single colony in LB broth containing 100 μg/ml ampicillin for 12 h at 37°C and shaking at 180 rpm. Cultures for protein production consisting of Terrific Broth (IBI Scientific, IB49140) containing 100 μg/ml ampicillin were inoculated with the starter culture and incubated at 37°C until OD_600_ reached ∼0.7, placed at 4°C for 30 min and induced with addition of 0.5 mM IPTG (isopropyl β-d-1-thiogalactopyranoside). Cultures were incubated for 18 h at 20°C with shaking at 180 rpm. Cells were harvested by centrifugation at 4°C for 45 min at 4000 rpm (Thermo Scientific, Sorvall LYNX 4000). Cells were resuspended and lysed in lysis buffer (50 mM Tris–HCl, pH 7.5, 300 mM NaCl, 20 mM imidazole, 1 mM TCEP [tris(2-carboxyethyl)phosphine], 5 mM MgCl_2_, 1 mM phenylmethylsulfonyl fluoride, EDTA-free protease inhibitor (Thermo Scientific, A32953), 1.2 mg/ml lysozyme (Sigma–Aldrich, L6876) and Benzonase^®^ Nuclease (Merck, E1014-5KU)) for 45 min at 4°C. Cells were additionally lysed by sonication (Qsonica Q700) and clarified by centrifugation at 12 000 rpm for 60 min at 4°C (Eppendorf, 5810R). The resulting cell lysate containing 6xHis-SUMO-CbAgo was then applied onto an Ni-NTA column (HisTrap HP, 5 ml, GE Healthcare), and affinity chromatography was performed using ÄKTA Pure (GE Healthcare). The sample was washed with buffer A (50 mM Tris–HCl, pH 7.5, 500 mM NaCl, 20 mM imidazole and 1 mM TCEP) to remove unbound proteins; recombinant 6xHis-SUMO-CbAgo was eluted using buffer B (50 mM Tris–HCl, pH 7.5, 500 mM NaCl, 300 mM imidazole and 1 mM TCEP). The 6xHis-SUMO tag was removed by SUMO protease digestion overnight in dialysis buffer [50 mM Tris–HCl, pH 7.5, 100 mM NaCl and 5% (v/v) glycerol]; the resulting solution was applied onto an Ni-NTA column for purification. Tag-free protein was collected and exchanged against low-salt buffer (50 mM Tris–HCl, pH 7.5, 100 mM NaCl and 1 mM TCEP) and applied to a cation-exchange column (HiTrap^®^ SP HP, 5 ml, GE Healthcare), washed with low-salt buffer and subsequently eluted with high-salt buffer (50 mM Tris–HCl, pH 7.5, 2 M NaCl and 1 mM TCEP). Protein was collected and exchanged into size exclusion chromatography buffer [25 mM Tris–HCl, pH 7.5, 100 mM NaCl, 1 mM TCEP and 10% (v/v) glycerol] and additionally purified by gel filtration on an S200 column (GE Healthcare). Fractions containing the protein were pooled, concentrated, snap-frozen in liquid nitrogen and stored at −80°C for downstream applications in *in vitro* experiments. All protein sequences are listed in Supplementary Table S1.

#### KmAgo purification

Recombinant KmAgo was purified from cells harboring the pET28a-6xHis-HRV3C-KmAgo plasmid (gift from Prof. Lixin Ma at Hubei University, Wuhan, China) as previously reported by Liu *et al.* (Supplementary File S1B; Supplementary Table S1) (14).

### Design and synthesis of different γPNA and γtcPNA molecules

γPNA and γ-substituted tail-clamp PNA (γtcPNA) molecules were designed based on previous reports (23,29) and custom synthesized by PANAGENE Inc. based on general PNA synthesis guidelines (https://www.pnabio.com/support/PNA_Tool.htm). All γPNAs and γtcPNAs were γ-modified with alanine molecules and three lysine moieties to facilitate solubility and invasion efficiency (Supplementary Table S2).

### Cloning of γPNA invading target regions into pMRS and pUC19 plasmids

γPNA invading target inserts were purchased as forward and reverse oligonucleotides from Integrated DNA Technologies Inc., with overhangs corresponding to BamHI and EcoRI restriction sites for cloning in pMRS and pUC19 vectors (Supplementary File S2A and B). All target sequences are listed in Supplementary Table S3. Oligonucleotides were independently phosphorylated using T4 PNK (Promega) before annealing. Plasmids were digested using BamHI-HF and EcoRI-HF in 1× CutSmart buffer (NEB) for 8 h. Phosphorylated dsDNA inserts were mixed with 10 ng of digested plasmid purified from agarose gel in 3:1 molar ratio and incubated with T4 DNA Ligase (Promega) in 1× T4 DNA Ligase Reaction Buffer (Promega; catalog # M1801) for 3 h at 23°C, followed by 2 h at 16 and 4°C until transformation of *E. coli* competent cells (Thermo Scientific, One Shot™ TOP10 Chemically Competent *E. coli*, C404010) with 2 μl of the ligation reaction using a heat shock method. Transformed cells were plated on agar plates containing 50 μg/ml kanamycin (pMRS) or 100 μg/ml ampicillin (pUC19). Plasmids were isolated from bacterial liquid cultures using a QIAprep Spin Miniprep Kit (Qiagen, 27106) and adjusted to 100 ng/μl for downstream applications. All clones were confirmed by Sanger sequencing using the primers listed in Supplementary Table S4.

### PNA invasion and mobility shift assay

PNA invasion tests were performed in 10 μl reaction volume consisting of 1× MOPS buffer (20 mM MOPS, pH 7.0, 5 mM CH_3_COONa and 1 mM EDTA), 50 nM linear dsDNA target (∼350 bp) and 2 μM PNA. Target dsDNA was generated by PCR amplification from pMRS plasmid using primers flanking the PNA target regions (1442 and 1557, Supplementary Table S4). Reactions were incubated overnight at 37°C, mixed with 5× Novex࣪ Hi-Density TBE Sample Buffer (Invitrogen, LC6678) and run on a Novex™ 6% native TBE gel at 180 V for 1 h. Gel was stained with 1× SYBR Gold (Invitrogen, S11494) for <10 min and visualized using a Molecular Imager Gel Doc XR+ System (Bio-Rad).

### Argonaute *in vitro* cleavage assay of circular and linear DNAs

All circular plasmid DNA targets were linearized by restriction digest with the desired restriction enzyme; the resulting linear products were purified with a QIAquick^®^ Gel Extraction Kit (28706) as per the manufacturer’s guidelines. Prior to pAgo protein cleavage, DNA targets were incubated with the two PNA molecules for 45 min (for circular DNA) or overnight (for linear DNA) at 37°C. PNA invasion reactions were conducted in 10 μl reaction volume containing 200 ng of DNA template (pMRS target; ∼5.3 kb, corresponding to approximately 6 nM), 1× MOPS buffer, PNA1 (final concentration of 100 nM) and PNA2 (final concentration of 100 nM). Further, pAgo-mediated cleavage was achieved in two steps: In the first step, pAgo was loaded with two different guides independently in half reaction 1 and half reaction 2 at 37°C for 15 min. All guide sequences used in this study are listed in Supplementary Table S5. Each half reaction contained 1× final pAgo reaction buffer [1× composition: 10 mM HEPES–NaOH, pH 7.0, 100 mM NaCl, 1 mM MnCl_2_ and 5% (v/v) glycerol], 1 μM guide and 1 μM recombinant pAgo in the total volume of 8 μl, unless stated otherwise. In the second step after guide loading, the two half reactions (8 μl each) were combined in one tube, to which 4 μl of PNA-invaded or non-invaded plasmid DNA was added (80 ng target DNA final, corresponding to approximately 1.2 nM). The total volume of the cleavage reaction was 20 μl. Each cleavage reaction was incubated at 37°C for 60 min, with the lid temperature set to 39°C. In the case of circular plasmid DNA, following the pAgo cleavage reactions, 2 μl of 10× CutSmart and 1 μl of the appropriate restriction enzyme (10 units/1 μl) were added to the reaction and incubated at 37°C for 30 min. Next, 1 μl of proteinase K (Invitrogen; catalog # 25530049) was added, followed by incubation at 37°C for 30 min. Four microliters of 6× Gel Loading Dye Purple (NEB; catalog # B7024S) was added to each sample, which was then loaded onto a 0.9% (w/v) agarose gel with GelRed^®^ and electrophoresed for 1 h and 30 min at 145 V. Finally, the gel was visualized using a FluorChemQ Gel Doc system. All reactions were set up at room temperature. The table with detailed protocol can be found in Supplementary Data.

### pAgo cleavage site identification

For the determination of the cleavage site, γPNA5 + γPNA6 binding target region was cloned into the pMRS plasmid at BamHI and EcoRI restriction sites such that the regions invaded by γPNA5 + γPNA6 were located on the opposite strand of the primer binding site, since γPNAs are known to inhibit strand elongation. Cleavage of targets γPNA5 + γPNA6 in pMRS linearized by BsrGI restriction digest was performed as described above; the products were separated from 1% (w/v) agarose gels. The bands corresponding to cleavage products were excised and purified from the agarose gel using a QIAquick^®^ Gel Extraction Kit (28706) and subsequently sequenced by Sanger sequencing using specific primers (Supplementary Table S4). Cleavage sites were determined by identifying read termination sites.

## RESULTS

### Overview and working principle of the PNP editors

**Figure 1.**
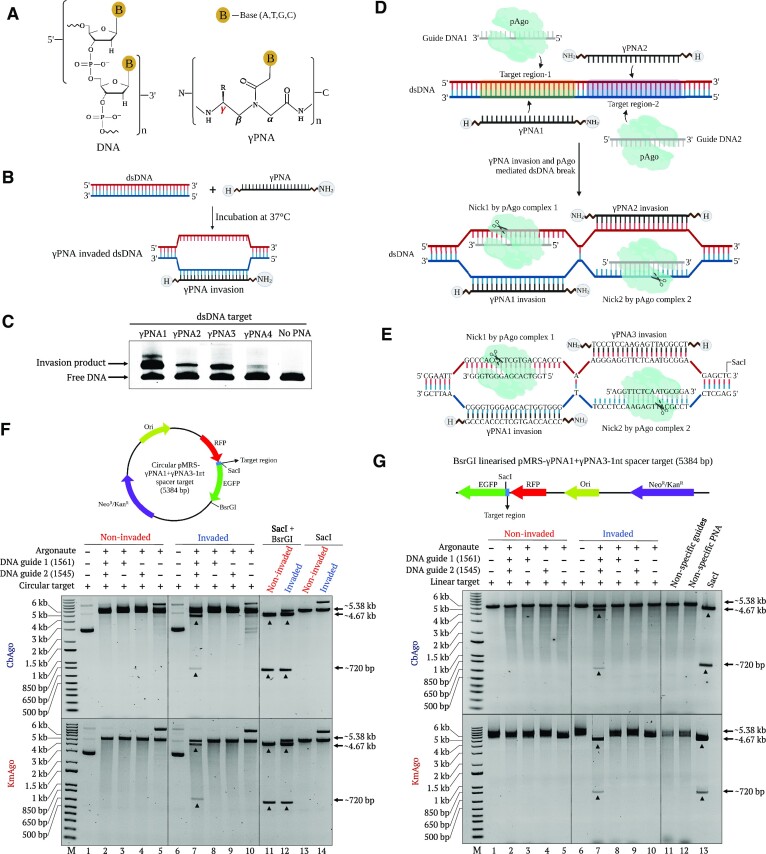
PNA invasion and pAgo-mediated cleavage of dsDNA molecules. (**A**) Structure comparison of DNA and PNA molecules. PNA modifications were added at γ position. (**B**) Schematic illustration of γPNA invasion of dsDNA. (**C**) Invasions of different γPNA molecules (γPNA1, γPNA2, γPNA3 and γPNA4) into a specific dsDNA target shown by a mobility shift assay. PCR amplicons (50 nM final) containing the target region specific for each γPNA were invaded overnight with 2 μM γPNA and resolved on native 6% TBE polyacrylamide gel. (**D**) Overview of the PNP concept. The ability of γPNA to specifically invade target dsDNA can be exploited to facilitate cleavage mediated by gDNA-loaded pAgo of any DNA by cleaving the displaced strand opposite from the γPNA invasion site to generate DSBs. (**E**) Schematic diagram of pAgo-mediated cleavage of the pMRS-γPNA1 + γPNA3 plasmid invaded by γPNA1 and γPNA3. (**F**) Gel images showing the pAgo-mediated cleavage of circular dsDNA invaded by γPNA1 and γPNA3. Upper and lower gels represent CbAgo and KmAgo cleavage, respectively. γPNA1 and γPNA3 were used to invade high-GC-content target regions (75% and 55%, respectively) cloned in the pMRS-γPNA1 + γPNA3 plasmid in close proximity to a SacI site. The bands corresponding to anticipated cleavage products were observed only in the reaction containing the invaded plasmid and the two gDNAs following the BsrGI digest (lane 7). (**G**) Gel images showing the pAgo-mediated cleavage of the target region in BsrGI-linearized dsDNA invaded by γPNA1 and γPNA3. Upper and lower gels represent CbAgo and KmAgo cleavage, respectively. γPNA1 and γPNA3 were used to invade high-GC-content target regions (75% and 55%, respectively) in pMRS-γPNA1 + γPNA3 plasmid linearized with BsrGI, in close proximity to a SacI site. The bands corresponding to expected cleavage products were observed in the reaction containing invaded linear plasmid and the two pAgo–gDNA complexes (lane 7). Lane M represents the 1-kb plus DNA ladder.

pAgo proteins lack intrinsic helicase activity and cannot unwind dsDNA targets to cleave them, limiting their adoption for biotechnological applications such as genome editing. The ability of different PNA molecules to invade dsDNA in a highly specific manner prompted us to investigate whether this property might be exploited to assist mesophilic pAgos in performing site-specific DSBs (32). Addition of functional groups to the γ-position of PNAs pre-organizes the helix, facilitating invasion of the canonical B-DNA form. We therefore tested the invasion efficiency of different γPNAs and confirmed that these molecules can invade DNA substrates, albeit with different efficiency (Figure 1A–C).

Encouraged by this result, we designed our concept based on the simultaneous use of two γPNA molecules capable of invading DNA substrates, in close proximity and on opposing DNA strands. The resulting invasion into the DNA helix would make a segment of displaced ssDNA available for pAgo enzymatic activity (Figure 1D). Simultaneous nicking on opposing DNA strands by the two pAgo–guide complexes should generate DSBs that could be harnessed for diverse *in vitro* and *in vivo* genome engineering applications (Figure 1D).

### pAgos generate site-specific DSBs on DNA substrates independently of GC content and DNA form

To test the above concept, we designed two γPNA molecules (γPNA1 and γPNA3) to invade specific DNA sequences cloned in the pMRS plasmid on opposite strands, separated by a single-nucleotide spacer (Figure 1E). We produced and purified recombinant CbAgo and KmAgo from *E. coli*. We then loaded these proteins with corresponding 5′-phosphorylated gDNAs (5′ P-gDNAs) and incubated the resulting pAgo complexes with intact plasmid substrate or a plasmid substrate that had been invaded by γPNA1 and γPNA3. We determined that non-invaded circular plasmid is nicked by CbAgo and KmAgo in a guide-independent manner without generating DSBs (Figure 1F). In contrast, we detected the generation of guide-dependent DSBs on PNA-invaded plasmid substrates, indicative of double-nicking activities that resulted in the release of a DNA fragment of the expected size following restriction digestion (Figure 1F).

We also tested the double-nicking activity and generation of specific DSBs on linear DNA substrates; to this end, we linearized the pMRS plasmid by BsrGI restriction digest and used the resulting linear DNA molecule as substrate (non-invaded or invaded with γPNA1 and γPNA3) for the same pAgo cleavage assay as above. We observed that CbAgo and KmAgo can specifically bind to linear DNA substrates that had been invaded with the two γPNAs to generate DSBs, as evidenced by the release of the band of the expected size (Figure 1G). Non-invaded DNA substrates did not show any band release, suggesting that γPNA invasion is a necessary prerequisite for pAgo binding and cleavage (Figure 1G).

**Figure 2.**
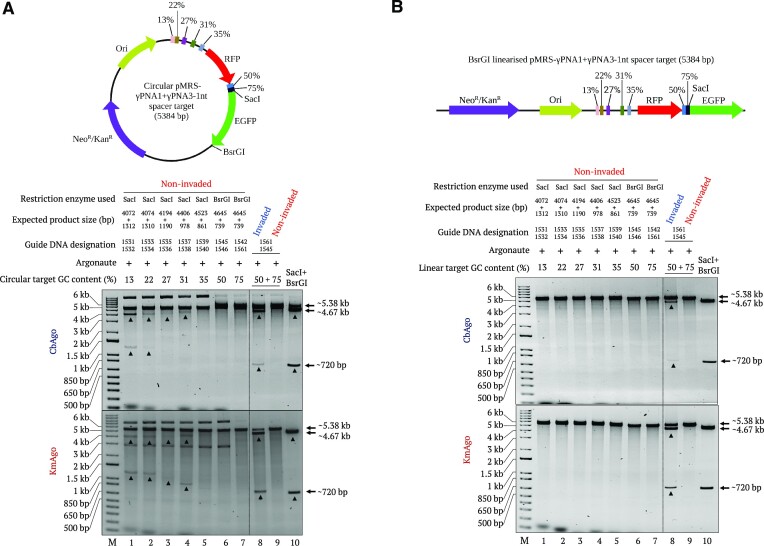
Effect of GC content on cleavage of circular and linear dsDNA. (**A**) Effect of GC content on cleavage of a circular plasmid. Upper and lower gels represent CbAgo- and KmAgo-mediated cleavage, respectively. Non-invaded pMRS-γPNA1 + γPNA3 circular plasmid was incubated with pairs of pAgo–gDNA complexes targeting regions with GC contents of 13%, 22%, 27%, 31%, 35%, 50% or 75% (lanes 1–7) at 37°C for 1 h. The same plasmid was then invaded by γPNA1 and γPNA3 at regions with GC contents of 55% and 75%, respectively, and incubated with their respective pAgo–gDNA complexes at 37°C for 1 h (lane 8). Following pAgo cleavage reaction, plasmids were digested with SacI or BsrGI depending on the position of the target region relative to the restriction enzyme site. Non-invaded pAgo cleavage (lane 9) and SacI + BsrGI-digested (lane 10) samples are included as control reactions. (**B**) Effect of GC content on cleavage of a linearized plasmid. Upper and lower gels represent CbAgo- and KmAgo-mediated cleavage, respectively. Linearized, non-invaded pMRS plasmid was incubated with pairs of pAgo–gDNA complexes targeting regions with GC content of 13%, 22%, 27%, 31%, 35%, 50% and 75% (lanes 1–7) at 37°C for 1 h. Linearized plasmid was then invaded by γPNA1 and γPNA3 at regions with 55% and 75% GC content, respectively, and incubated with the respective pAgo targeting complexes at 37°C for 1 h (lane 8). Non-invaded pAgo-mediated cleavage (lane 9) and SacI-digested (lane 10) samples were used as control reactions. Expected cleavage product sizes are listed on top of each gel lane. Lane M represents the 1-kb plus DNA ladder.

CbAgo and KmAgo nuclease activity on supercoiled DNA substrates *in vitro* is limited to regions of low GC content at physiological temperatures, and decreases inversely proportionally with increasing GC content, indicating that strand unwinding is one of the limiting factors for harnessing the power of pAgo for biotechnological applications (13,14,33). We asked whether the barrier to DNA unwinding presented by high GC content in the target sequence might be overcome by employing our concept. Accordingly, we assessed the induction of DSB formation in non-invaded circular and linear DNA targets at regions with increasing GC content ranging from 12% to 75%. In agreement with previous studies, we detected some catalytic activity for CbAgo and KmAgo on circular plasmids with target sequences of low GC content. Specifically, we established that CbAgo exhibits catalytic activity on DNA sequences with GC contents of 13% and 22%, whereas KmAgo exhibited catalytic activities on DNA sequences with GC contents of 31% and below (Figure 2A). However, neither CbAgo nor KmAgo showed any catalytic activity on linear DNA substrates, even over low-GC-content regions (Figure 2B). Notably, CbAgo and KmAgo demonstrated robust catalytic activities on DNA sequences with GC contents of 50% and 75% if DNA had been previously invaded by the corresponding PNAs, indicating that GC content and DNA form are not limiting factors for our PNP editors, thus unlocking the activities of pAgos on all DNA substrates at arbitrary sequences.

### Effect of different γPNA combinations, γPNA length and different types of PNAs on PNP editor activity

To test the versatility and programmability of the PNP editor concept, we designed two additional γPNA molecules, cloned their respective targets in various combinations (γPNA1 + γPNA2, γPNA3 + γPNA4, γPNA2 + γPNA4 and γPNA1 + γPNA3) in the pUC19 plasmid and assessed the formation of DSBs (Supplementary Figure S1A). We established that all γPNA combinations result in pAgo-mediated site-specific DNA cleavage, albeit with variable efficiencies that are likely due to varying invasion efficiencies of different γPNA molecules (Supplementary Figure S1B). In particular, the γPNA1 + γPNA3 and γPNA3 + γPNA4 combinations displayed the highest efficiency for generating DSBs. Notably, the efficiency of DSB generation by CbAgo and KmAgo followed the same pattern, indicating that γPNA invasion efficiency is the limiting step in the reaction.

We also investigated whether a specific PNA length is required for proper function of PNP editors. For this purpose, we synthesized a range of truncated γPNA1 and γPNA3 molecules ranging from 10 to 20 nt in length. We invaded the linearized pMRS vector containing the cloned target regions with these shorter γPNA molecules. We also preloaded pAgo with a 16-nt-long gDNA and performed cleavage reactions (Supplementary Figure S2A and B). We detected cleavage products only when γPNA1 and γPNA3 were 20 nt in length, suggesting that both CbAgo and KmAgo require the stretch of PNA-displaced ssDNA to be longer than the corresponding guide for optimal activity. To confirm this observation, we employed different γPNAs and guides of equal length to cleave the linearized pMRS plasmid. We observed weak activity only when employing 20-nt-long PNAs and corresponding guides of the same length and no activity from shorter pairs of PNAs and guides (Supplementary Figure S2C and D). We speculate that this activity might be attributed to a larger segment of exposed ssDNA available for pAgo activity generated specifically by the longest γPNA employed.

We set out to expand the PNP concept by using γtcPNA for DNA invasion. γtcPNA forms a triplex structure with target DNA via both Watson–Crick and Hoogsteen base pairing. Owing to this property, γtcPNA forms exceptionally stable invasion products that can be harnessed for use with PNP editors. We designed two γtcPNA molecules and constructed target plasmids containing their complementary DNA sequences. The mobility shift assay showed that γtcPNA can efficiently invade dsDNA templates (Supplementary Figure S3A). Cleavage assays also revealed efficient activity of a linear dsDNA target invaded by γtcPNA1 and γtcPNA2 with both CbAgo and KmAgo, whereas the non-invaded target exhibited no cleavage (Supplementary Figure S3B and C). These results demonstrate that DNA unwinding can be achieved by triplex-forming PNAs, without steric hinderance affecting pAgo binding and cleavage.

### Multiplexed, site-specific generation of DSBs using PNP editors on plasmid DNA

**Figure 3.**
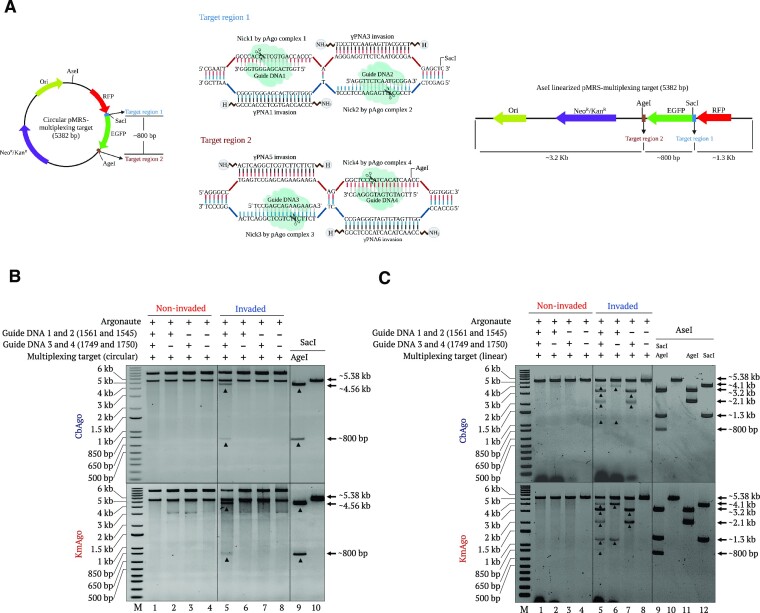
Multiplexed cleavage of circular plasmid containing two target sites with two pairs of γPNAs and pAgo–guide complexes. (**A**) Schematic diagram of the two target regions in the circular and AseI-linearized pMRS multiplexing plasmid. Target region 1 contains the γPNA1 + γPNA3 binding sequences; target region 2 contains the γPNA5 + γPNA6 binding sequences. The two target regions are separated by 820 bp (EGFP sequence). (**B**) Representative gel images showing the multiplex cleavage of circular plasmid with two pairs of pAgo–gDNA complexes. Upper and lower gels represent CbAgo- and KmAgo-mediated cleavage, respectively. The plasmid was initially invaded with two pairs of γPNA molecules (γPNA1 + γPNA3 and γPNA5 + γPNA6) and incubated with pAgo loaded with gDNA for 1 h at 37°C. Non-invaded and invaded plasmids were incubated with the two pairs of pAgo–gDNA complexes (lanes 1 and 5, respectively), one gDNA pair (lanes 2–3 and 6–7) or no gDNA (lanes 4 and 8). SacI + AgeI-digested (non-invaded) (lane 9) and SacI-linearized (non-invaded) (lane 10) samples were used as controls. Lane M represents the 1-kb plus DNA ladder. (**C**) Multiplex cleavage of linear plasmid with two pairs of pAgo–gDNA complexes. Upper and lower gels represent CbAgo- and KmAgo-mediated cleavage, respectively. The AseI-linearized plasmid was invaded with two pairs of γPNA molecules (γPNA1 + γPNA3 and γPNA5 + γPNA6) overnight and incubated with a pair of pAgos (1.5 μM final) loaded with gDNA for 2.5 h at 37°C. Non-invaded and invaded plasmids were incubated with the two pairs of pAgo–gDNA complexes (lanes 1 and 5, respectively), one gDNA pair (lanes 2–3 and 6–7) or no gDNA (lanes 4 and 8). AseI-, SacI- and AgeI-digested (non-invaded) (lanes 9–12) samples were used as controls to visualize positions of resulting bands. Lane M represents the 1-kb plus DNA ladder.

To demonstrate that PNP editors can simultaneously induce multiple DSBs, we constructed a pMRS plasmid containing two regions each targeted by a different pair of PNP editors (Figure 3A). We performed the assay using CbAgo and KmAgo following simultaneous γPNA invasion at two different regions on circular and linear plasmid DNA. We observed the release of a 820-bp fragment and all expected bands in both cases, indicating that all four γPNAs invaded the DNA substrate, which facilitated pAgo binding and cleavage. In contrast, targeting the PNP editors to a non-invaded DNA template failed to release any fragment (Figure 3B and C).

### Effect of spacer length and guide orientation on PNP editor activity

**Figure 4.**
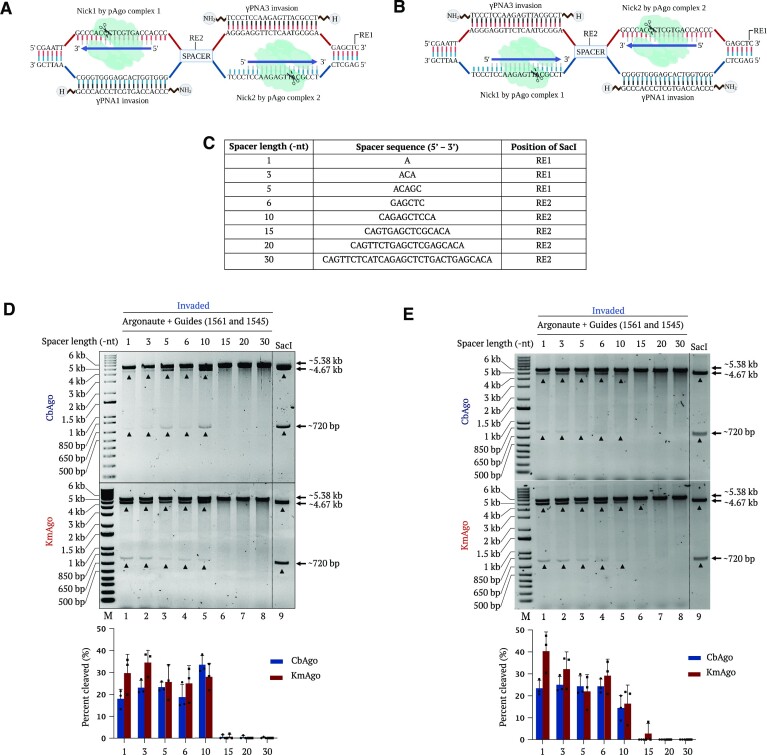
Testing the effect of spacer length between γPNA1 and γPNA3 target regions in pMRS plasmid on cleavage efficiency. (**A**) Schematic diagram showing the γPNA1 and γPNA3 binding regions with guides facing outward. (**B**) Schematic diagram showing the γPNA1 and γPNA3 binding regions with guides facing inward. RE1 and RE2 indicate the positions of the SacI restriction sites in different target plasmids. (**C**) Table summarizing the different spacer sequences, lengths (1, 3, 5, 6, 10, 15, 20 and 30 bp) and the position of SacI on the target plasmid. (**D**) Representative gel images showing pAgo-mediated (upper, CbAgo; lower, KmAgo) cleavage of DNA targets invaded by γPNA1 and γPNA3 (guides facing outward) containing varying spacer lengths between the two PNA invasion sites. All plasmids were linearized using BsrGI digestion and invaded overnight with γPNA1 and γPNA3 before being cleaved with a pair of pAgo–gDNA complexes (lanes 1–8). SacI + BsrGI-restricted (non-invaded) (lane 9) sample was used as size control. (**E**) Representative gel images of pAgo-mediated (upper, CbAgo; lower, KmAgo) cleavage of DNA targets invaded by γPNA1 and γPNA3 (guides facing inward) containing different spacer lengths between the two PNA invasion sites. All plasmids were linearized using BsrGI digestion and invaded overnight with γPNA1 and γPNA3 before being cleaved with a pair of pAgo–gDNA complexes (lanes 1–8). A SacI + BsrGI-digested (non-invaded) (lane 9) sample was used as size control. Lane M represents the 1-kb plus DNA ladder. Quantification values are shown as mean ± standard deviation (SD) (*n* = 3).

Our concept depends on PNA invasion of sequences at opposite DNA strands, leading to the displacement of short stretches of ssDNA that serve as substrates for pAgo recognition and cleavage (Figure 4A and B). Therefore, spacer length between the two γPNA-invaded strands and the orientation of the two guides may be key parameters for the design of efficient PNP editors (Figure 4C). We tested PNP editor efficiency on targets with different spacer lengths (1, 3, 5, 6, 10, 15, 20 or 30 nt) guided by pAgos with gDNAs in two distinct orientations (inward and outward) (Figure 4D and E). CbAgo activity increased with longer spacers, reaching a peak on targets with 10-nt spacers when using outward-facing guides. In contrast, we noticed an overall lower activity for CbAgo with inward-facing guides, as we detected slightly higher activity on targets with shorter spacers (Figure 4E). KmAgo showed higher cleavage efficiency with shorter spacers, peaking at 1-nt spacer length (Figure 4D and E). Unlike CbAgo, KmAgo activity was independent of guide orientation, possibly reflecting structural and steric differences in target DNA binding. Finally, in all cases, target plasmids with spacer lengths of at least 15 nt did not show any cleavage (Figure 4D and E).

### pAgo guide requirements for efficient generation of DSBs

**Figure 5.**
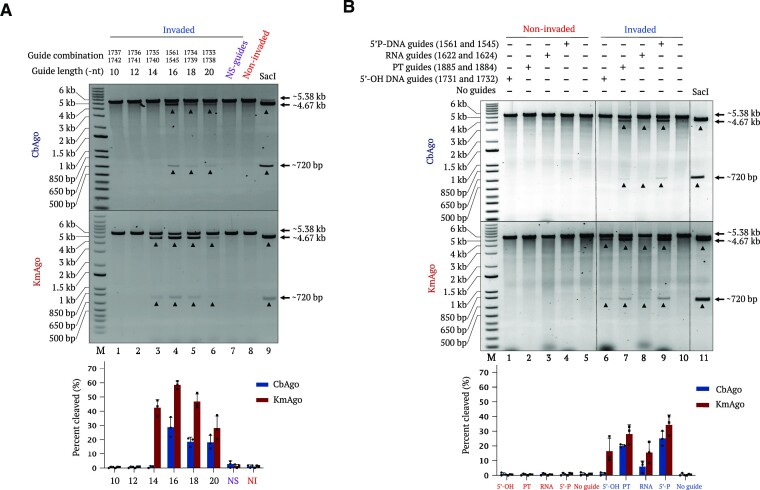
Testing the effect of guide type and length on pAgo-mediated cleavage efficiency on linear DNA. (**A**) Representative gel images showing pAgo-mediated cleavage (upper gel, CbAgo; lower gel, KmAgo) of a linear DNA target invaded by γPNA1 and γPNA3 employing different types of guides. Linearized pMRS-γPNA1 + γPNA3 plasmid, either non-invaded or invaded by γPNA1 + γPNA3, was incubated with pAgo loaded with 5′-hydroxylated DNA guides (5′-OH DNA guides) (lanes 1 and 6, respectively), phosphorothioated guides (PT-guides; PT bond at every position) (lanes 2 and 7, respectively), 5′-phosphorylated RNA guides (RNA guides) (lanes 3 and 8, respectively), 5′-phosphorylated DNA guides (5′ P-DNA guides) (lanes 4 and 9, respectively) or no guides (lanes 5 and 10, respectively). SacI-digested plasmid DNA (non-invaded) (lane 11) was used as size control. (**B**) Representative gel images showing the effect of guide length on cleavage efficiency of plasmid DNA linearized by BsrGI digestion and invaded by γPNA1 and γPNA3. Plasmid DNA invaded by γPNA1 and γPNA3 was incubated with pAgo (upper gel, CbAgo; lower gel, KmAgo) preloaded with guide DNAs of 10, 12, 14, 16, 18 or 20 nt in length, or with nonspecific guides (lanes 1–7). Non-invaded plasmid with 16-nt guides and SacI-digested DNA (non-invaded) (lane 11) were included as control reactions. Lane M represents the 1-kb plus DNA ladder. Quantification values are shown as mean ± SD (*n* = 3).

gDNA length is a key factor for robust pAgo catalytic activity. Therefore, we determined the best-suited length for gDNA to achieve optimal performance of CbAgo and KmAgo on dsDNA templates invaded by γPNAs of 20 nt in length. To this end, we used 5′ P-gDNAs of different lengths and performed catalytic activity assays on linearized pMRS plasmid containing γPNA1 and γPNA3 target regions. We determined that a guide length of 16 nt is optimal for CbAgo and KmAgo catalytic activity, resulting in the generation of DSBs evidenced by the release of a band of the expected size (Figure 5A). This finding is consistent with reported optimal guide length requirements for CbAgo and KmAgo activity on ssDNA (12,33). In conclusion, both pAgos perform optimally with 16-nt-long guides, but KmAgo demonstrated higher flexibility by employing guides with a broader range of lengths than CbAgo.

pAgos were shown to mostly use 5′-phosphorylated short DNA molecules to direct their activity *in vitro* and *in vivo*. We asked whether the 5′ modification of gDNA molecules would affect pAgo activity on their PNA-invaded dsDNA substrates. Moreover, we wanted to explore whether CbAgo and KmAgo might bind to PT-guides for potential application in living organisms to prevent guide degradation by nucleases. First, we preloaded CbAgo and KmAgo with guides harboring phosphorothioate groups at different positions and tested their activity on ssDNA targets. We observed good activity in all cases; however, in the case of both Agos, we observed a modest decrease in activity as the number of PT modifications in the guide increased, with the most marked reduction when using fully phosphorothioated guides. Introduction of small number of PT modifications at the 5′ and 3′ guide ends had little effect on CbAgo, but led to a more pronounced decrease in activity of KmAgo when 5′ modified guides were used (Supplementary Figure S4A–C). We also performed cleavage assay on invaded and non-invaded linearized plasmid DNA substrates using different types of guide molecules. We loaded pAgos with 5′-OH DNA guides, 5′-phosphorylated PT-guides, RNA guides and 5′ P-DNA guides. Consistent with the data on ssDNA, we determined that different guide modifications significantly affect cleavage activity of CbAgo and KmAgo. Both pAgos preferred 5′ P-DNA guides, as expected (Figure 5B). Our data indicated that both pAgos can employ PT-guides with only a modest reduction in cleavage efficiency. Moreover, only KmAgo could employ 5′-OH DNA guides, but with markedly lower efficiency compared to 5′-phosphorylated guides. As previously reported, CbAgo and KmAgo could bind to 5′ P-RNA guides to target DNA, although with substantially lower activity (Figure 5B). These results demonstrate versatile modes to guide PNP editors. Nevertheless, our data confirm that 5′ P-DNA guides are preferred for optimal activity, with phosphorothioate bonds in the backbone offering a potentially more viable strategy to guide PNP editors in the context of cellular environments.

### PNP editor efficiency at dsDNA flanking regions

We speculated that each γPNA might expose a wider region of ssDNA next to the invasion site and render it targetable by PNP editors. We also wondered whether targeting the non-invaded region flanking the invaded strand with only one γPNA would result in noticeable cleavage of dsDNA in a circular plasmid. We thus designed guides targeting both flanks of invaded pUC19 plasmid as well as guides targeting invaded strands (Supplementary Figure S5A). We observed that pAgos directed against the double-stranded regions flanking the γPNA invasion sites as well as invaded DNA strand fail to generate cleavage products. As previous experiments showed, CbAgo and KmAgo mediated cleavage only when directed to free ssDNA strands exposed by γPNA invasion (Supplementary Figure S5B).

To expand the range of usable gDNAs and elucidate the effect of neighboring dsDNA sequences, we designed guides that can target regions spanning the unwound regions in both inward and outward orientations and tested them on linearized pMRS plasmid. We defined inward orientation as 5′ ends of guides facing each other and outward orientation as 5′ ends facing away (Supplementary Figures S6A and S7A). When using outward-facing guides, we detected cleavage with guides that contained up to 11 nt in the dsDNA sequence flanking the γPNA invasion (Supplementary Figure S6B). We obtained similar results with inward-facing guides, where the flanking region was located between two invaded regions. In this case, we observed cleavage bands with guides up to 10 nt (with CbAgo) or up to 8 nt (with KmAgo) in the dsDNA region (Supplementary Figure S7B). This experiment revealed that pAgos exhibit catalytic activity even when guides target partially unwound DNA, likely due to the limited ability of gDNA to displace neighboring dsDNA regions. However, a pre-existing ssDNA region corresponding to roughly half the length of the gDNA must be present to result in activity.

### Effect of temperature on γPNA invasion and pAgo activity

**Figure 6.**
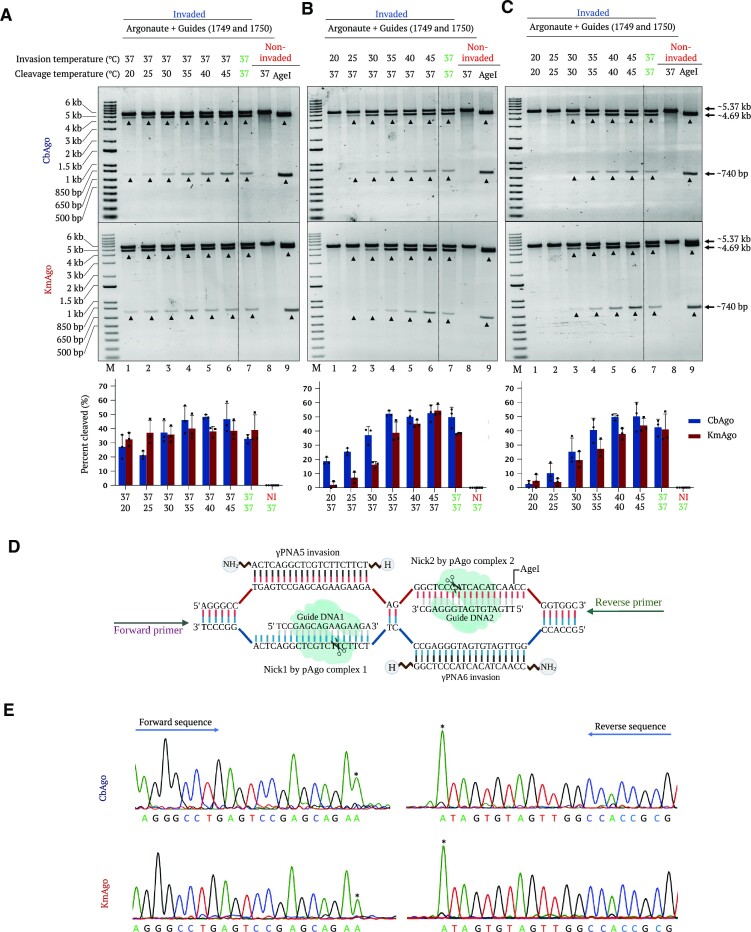
Testing the effect of temperature on PNA invasion, pAgo cleavage and identification of pAgo cleavage sites. (**A**) Representative gel images showing pAgo activity at different temperatures. To test the ability of pAgos to cleave the invaded target at a range of temperatures, linearized plasmid containing γPNA5 + γPNA6 binding regions was invaded with γPNA5 and γPNA6 at 37°C overnight. The target was then incubated with a pair of pAgo complexes (upper gel, CbAgo; lower gel, KmAgo) targeting the displaced ssDNA region at 20, 25, 30, 27, 40 or 45°C for 1 h (lanes 1–6). (**B**) Representative gel images showing invasion by γPNA5 and γPNA6 at different temperatures. Linearized plasmid containing γPNA5 and γPNA6 binding regions was invaded at 20, 25, 30, 27, 40 or 45°C overnight, followed by cleavage by pAgo at 37°C for 1 h (lanes 1–6). (**C**) Representative gel images showing invasion by γPNA5 and γPNA6 and pAgo activity at different temperatures. Both the invasion step and pAgo cleavage were performed under the same varying temperature (20, 25, 30, 27, 40 or 45°C) conditions. The invasion step was performed overnight, followed by pAgo (upper gel, CbAgo; lower gel, KmAgo) cleavage assay for 1 h (lanes 1–6). In all gels (A, B and C), samples invaded with γPNA5 and γPNA6, non-invaded templates cleaved with pAgo (upper gel, CbAgo; lower gel, KmAgo) at 37°C (lanes 7 and 8, respectively) and AgeI-digested DNA (non-invaded) (lane 9) were included as control reactions. Lane M represents the 1-kb plus DNA ladder. Quantification values are shown as mean ± SD (*n* = 3). (**D**) Schematic diagram showing invasion by γPNA5 and γPNA6, pAgo–guide complex binding sites and primer orientation for Sanger sequencing. (**E**) Determination of pAgo cleavage site on a dsDNA template linearized by BsrGI digestion and invaded by γPNA5 and γPNA6. Confirmation of pAgo (upper gel, CbAgo; lower gel, KmAgo) cleavage site was performed on invaded pMRS-γPNA5 + γPNA6 that had been linearized by BsrGI digestion. Following the cleavage assay, the bands corresponding to cleavage products were purified from the agarose gel and subjected to Sanger sequencing. Asterisks denote sequencing artifacts arising from AmpliTaq adding a non-templated 3′ A upon reaching the end of a linear template.

Robust γPNA invasion and catalytic activity of the pAgo proteins at physiological temperatures are necessary to harness the power of PNP editors for potential genome-editing applications in various organisms. To elucidate the optimal temperature conditions required for γPNA invasion and pAgo activity, we used the pMRS plasmid linearized by BsrGI digest and invaded with γPNA5 and γPNA6 at different temperatures. To determine the performance of pAgos at different temperatures, we performed γPNA invasion at 37°C and incubated the target with CbAgo or KmAgo at temperatures ranging from 20 to 45°C. Both proteins mediated robust catalytic activities at temperatures as low as 20°C (Figure 6A). Both proteins demonstrated cleavage at all temperature conditions, even displaying increasing activity at elevated temperatures (Figure 6A). γPNA invasion is critical to expose the ssDNA strand for pAgo activity; hence, it is important to determine whether it occurs at physiologically relevant temperatures. Therefore, we performed an overnight invasion of linearized pMRS plasmid with γPNA5 and γPNA6 at temperatures ranging from 20 to 45°C and conducted pAgo cleavage at 37°C to ascertain strand invasion. We observed that γPNA invasion occurs more efficiently at higher temperatures, effectively starting at 25°C (Figure 6B). Finally, we tested both γPNA5 and γPNA6 invasion and pAgo catalytic activity at the same temperature, which we varied from 20 to 45°C. When both steps were performed at lower temperatures, we detected a cumulative decrease in overall activity. Nevertheless, γPNA invasion and pAgo catalytic activity remained efficient at 30°C and improved when performed at elevated temperatures (Figure 6C).

### Determining the pAgo cleavage site in γPNA-invaded dsDNA

Previous studies have shown that most pAgos cleave DNA targets between nucleotides 10 and 11 from the 5′ end of the guide, with some notable exceptions (33,34). Because the precise site of cleavage is an important consideration in genome engineering and related applications, we determined whether the cleavage site is conserved in the PNP editor concept. We thus identified the cleavage site catalyzed by CbAgo and KmAgo in DNA substrates (13,14). Accordingly, we performed a cleavage assay on BsrGI-linearized plasmid that had been invaded with γPNA5 and γPNA6, followed by gel purification of the released bands, which we subjected to Sanger sequencing (Figure 6D). We established that both proteins cut the displaced DNA strand of the PNA-invaded DNA substrates at their canonical site located between nucleotides 10 and 11 from the 5′ end of the gDNA. We also observed that PNA invasion does not affect the position of pAgo cleavage, making PNP editors amenable to generating site-specific staggered end DSBs (Figure 6E).

### Effect of gDNA mismatches on the activity of PNP editors

**Figure 7.**
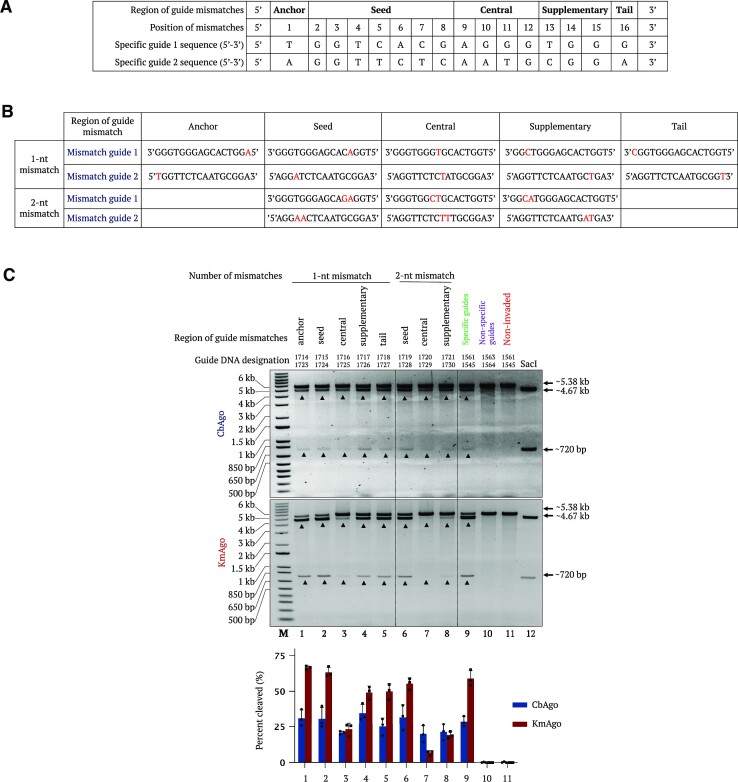
Effect of guide mismatches at different positions on pAgo cleavage efficiency of BsrGI-linearized plasmid DNA. (**A**) Table summarizing the different regions in the gDNA molecule. (**B**) Table summarizing the mismatched guides 1 and 2, highlighting the region, type and number of mismatched nucleotides. (**C**) Representative gel images showing the pAgo cleavage of pMRS-γPNA1 + γPNA3 plasmid linearized by BsrGI digestion and invaded by γPNA1 and γPNA3 using different mismatched guides. Invaded plasmid was incubated with CbAgo (upper gel) or KmAgo (lower gel) preloaded with different gDNAs containing 1- or 2-nt mismatches at different positions in the pAgo guide architecture for 1 h at 37°C (lanes 1–8). Reactions of samples invaded by γPNA1 and γPNA3 with specific guides and nonspecific guides (lanes 9 and 10, respectively) were included. Non-invaded samples but incubated with specific guides (lane 11) and SacI-digested samples (non-invaded) (lane 12) were included as control reactions. Lane M represents the 1-kb plus DNA ladder. Quantification values are shown as mean ± SD (*n* = 3).

gDNA architecture can be divided into several segments depending on their position from the 5′ end (13). In most studied pAgos, the first nucleotide at the 5′ end is the anchoring nucleotide and binds to the MID domain binding pocket. The seed region is composed of 8 nt from the 5′ end of the guide, mediates target recognition and is stabilized by the C-terminal lobe. The central part and the 3′ end bind to the PAZ domain (3). Several reports have shown that gDNA mismatches can be tolerated depending on their number and position. We tested the extent to which mismatches in different regions would compromise the PNP editor activity. We first introduced single- and double-nucleotide mutations in different parts of the guide molecule and in the anchor, seed, central, supplementary and tail regions (Figure 7A and B). Single-nucleotide mismatches were largely tolerated across the entire gDNA sequence. However, double-nucleotide mismatches had a larger effect and compromised pAgo activity most severely when located in the central and supplementary regions. Notably, KmAgo activity was completely abrogated with double-nucleotide mutations in the central guide region (Figure 7C). To determine the number of guide mismatches required to completely abolish pAgo activity, we tested up to four mismatches in the gDNA (Supplementary Figure S8A and B). Our results consistently showed that the cleavage efficiency of CbAgo and KmAgo gradually decreases with more mismatches and that mismatches in the central and supplementary regions close to the 3′ end of the guide have a greater effect on activity than those closer to the 5′ end (Supplementary Figure S8B). We observed complete loss of activity with CbAgo when the gDNA contained four mismatches regardless of their position. In contrast, KmAgo tolerated mismatches at the 5′ end of the guide, even with four mismatches.

### Effect of γPNA mismatches on the activity of PNP editors

The PNP concept relies on a PNA that specifically invades two opposing DNA strands to allow pAgo activity. Since cleavage specificity is paramount for applications in genome editing, we explored the effects of γPNA mismatches on the activity of PNP editors. We introduced mismatches on DNA substrates corresponding to the 3′, 5′, central and nonconsecutive regions of the γPNA sequence and tested their influence on pAgo activity. PNP editors in principle possess two layers of specificity resulting from specific PNA invasion and specific gDNA target recognition. Therefore, we tested specificity using guides that were perfectly complementary to the region with mismatched γPNAs and separately with mismatched guides and mismatched invading γPNA (Supplementary Figures S9A and S10A). We introduced four types of mismatches, positioned at the 5′ end and 3′ ends consisting of 1-, 2-, 3-, 4-, 5-, 7-, 9- or 11-nt mismatches. Additionally, we introduced up to four consecutive mismatches or two, four, six or eight nonconsecutive mismatches in the central region of the target DNA. We determined that the position and the number of mismatches between the target and γPNA have a decisive influence on PNP editor cleavage efficiency. We observed that γPNAs containing mismatches at their 5′ and 3′ termini still retain sufficient ability to invade target DNA, as evidenced by the band release seen on agarose gels, even when up to five mismatched nucleotides at the 3′ end and seven mismatched nucleotides at the 5′ end were introduced. In sharp contrast, introducing mismatches in the central region of the target resulted in a marked drop of pAgo activity, especially when using CbAgo. With both pAgos, 4-nt mismatches in the center of the target resulted in complete loss of cleavage activity, indicating the inability of γPNA to invade the target region. We detected the most pronounced effect, however, when γPNA was directed to invade regions containing nonconsecutive mismatches in the center of the target. We only observed a weak band in samples with KmAgo targeted to a target containing two mismatched nucleotides (Supplementary Figure S9). In the second set of experiments, we performed cleavage assays using gDNAs that contained the same number of mismatches and at the same positions relative to the γPNA. We observed that mismatches in the PNA regions and corresponding gDNA synergistically compromise the activity of both proteins. We detected pAgo activity only with one and two γPNA and gDNA nucleotide mismatches at the 3′ end, and one mismatched nucleotide at the 5′ end when using either pAgo. Only KmAgo showed activity when targeted to central mismatched regions and only with one mismatched nucleotide. Additionally, all nonconsecutive mismatches in the central region completely abrogated activity in all cases (Supplementary Figure S10).

Finally, we performed time course assay to determine the time required for PNA to invade different forms of dsDNA. We invaded circular and linearized plasmid DNA at 37°C for increasing periods of time, followed by Argonaute cleavage for 1.5 h. Our results indicated that circular and linear dsDNA can be efficiently invaded in as little as 10 min or 2 h, respectively (Supplementary Figure S11). Interestingly, in the case of circular plasmid, we observed that extended invasion time only resulted in a modest increase in PNP editor-mediated cleavage efficiency, regardless of whether CbAgo or KmAgo was employed. Conversely, for linear dsDNA, we noted that cleavage efficiency reached its peak after 8 h of PNA incubation but substantially declined after 16 h.

## DISCUSSION

The genome editing field has benefited from technologies harnessing natural molecular mechanisms combined with innovations in bioengineering. Zinc finger nucleases, transcription activator-like effector nucleases (TALEN) and clustered regularly interspaced short palindromic repeats (CRISPR) have been used for genome-editing applications in diverse eukaryotic species (35). These technologies have their advantages and limitations, the latter especially in the translational potential of these genome engineering platforms due to challenges in cargo delivery, specificity, toxicity and immunogenicity. Here, we introduced PNP editors, a novel platform that combines the targeted DNA strand invasion ability of PNA molecules with the DNA-guided programmability of pAgos to enable programmable generation of site-specific DSBs. The inability of pAgos to mediate DSBs on linear DNA of arbitrary GC content and physiological temperatures is attributed to their lack of intrinsic helicase activity. The application of various pAgos has so far been limited to manipulation of ssDNA, circular (supercoiled) dsDNA with low GC content, cleavage of circular dsDNA at elevated nonphysiological temperatures or enhancement of *in vivo* recombination in bacterial cells (9,14). We aimed to unlock the potential of pAgos for *in vivo* applications, including genome editing. pAgo activity at physiological temperatures is a prerequisite for their use in genome editing and other *in vivo* applications. Several studies have identified different pAgos from mesophilic bacteria with activity at a broad range of temperatures from 20 to 60°C. For example, CbAgo, KmAgo and LrAgo proteins have been identified and characterized (12–15,33). Although mesophilic pAgos such as KmAgo, CbAgo and LrAgo are known to be programmable with DNA guides to cut dsDNA sequences, such directed activity was very low against GC-rich sequences at 37°C, with efficient cleavage only shown at 55°C and at AT-rich regions. The activity of pAgos at physiological temperatures on linear dsDNA sequences of arbitrary GC content would unlock the potential of these proteins for *in vitro* and *in vivo* genome-editing applications. Although combining CbAgo with a RecBC helicase from *E. coli* was recently shown to mediate DSB generation in linear dsDNA, this strategy is yet to be explored for *in vivo* applications in eukaryotic cells (15). We addressed the absence of intrinsic helicase activity characteristic of pAgos by employing PNA molecules for targeted, site-specific invasion of any DNA substrate.

PNA molecules were initially introduced due to their ability to invade DNA and form anomalous PNA–DNA structures that trigger the cellular repair machinery to resolve this structure as a recombination event. Although this concept has been applied for over three decades, the efficacy of PNA-mediated genome modification remains low, rendering it impractical for clinical applications in gene therapy (24,30,36). Targeted PNA invasion of DNA nevertheless offers clear advantages, including simplicity, resistance to cellular nucleases and potential to unwind B-DNA helices at specific sites for recruitment of various DNA modifying enzymes. Furthermore, various synthetic DNA analogs that can invade DNA substrates may be used for gene editing and be incorporated into the PNP editor scheme. Examples include locked nucleic acid and Zorro oligonucleotides that can bind to both strands of the DNA simultaneously, which could facilitate *in vitro* and *in vivo* genome editing (37).

In this work, we coupled site-specific PNA invasion into target DNA to generate a displaced ssDNA strand that can be used as a substrate for guided pAgo nucleases. We showed that both pAgos tested here were capable of targeting DNA substrates irrespective of their GC content and DNA form at physiological temperatures, thus overcoming a major limitation in harnessing the power of pAgos in genome editing. We demonstrated that a DSB was generated only when pAgos were directed to displaced ssDNA regions and not when they targeted invaded strands or when the pAgo target region was flanking invasion sites.

The length and type of PNA is an important consideration when designing PNP editors. Here, we showed that PNA length should be greater than the length of the guide for optimal pAgo activity. Guide length of 16 nt in combination with 20-nt-long PNA led to the most efficient cleavage. Moreover, both γPNA and γtcPNA molecules efficiently invaded target DNA and showed similar effectiveness in facilitating pAgo cleavage. This observation indicated that any PNA molecule that can efficiently unwind dsDNA would likely facilitate robust pAgo cleavage. We also showed that spacer length between 1 and 10 nt between two PNA invasion sites was optimal to generate a DSB *in vitro*, thus informing the optimal PNP editor design for *in vivo* use. We established that protein orientation, as defined by guide orientation, did not influence KmAgo activity and that the protein showed better performance with shorter spacers. In contrast, CbAgo displayed overall lower activity and opposite spacer length preference with guides in the inward orientation and optimal activity with 10-nt spacers in the outward orientation. Another major limitation is the activity of pAgos at physiological temperatures. Importantly, both PNA molecules and pAgos performed well at, and below, physiological temperatures, indicating the potential for their use *in vivo* in various eukaryotic organisms such as zebrafish (*Danio rerio*), the nematode *Caenorhabditis elegans*, etc.

The canonical pAgo cleavage site was reported to shift under certain conditions, such as when using guides of shorter length (33). We confirmed that the position of nicking sites on PNA-invaded DNA was not altered due to the presence of a PNA and was located between nucleotides 10 and 11 from the 5′ end of the guide. Further characterization of gDNA requirements revealed that 1-nt mismatches in the gDNA were well tolerated at any position within the guide. However, introducing two or more mismatched nucleotides into the gDNA revealed that central and supplementary regions close to the 3′ end were especially sensitive to mismatches, leading to eventual complete abrogation of activity with four mismatched nucleotides. Our results are in agreement with previous reports, where Argonautes are directed to ssDNA regions (12–14,33).

The specificity of PNP editors depends on two events: site-specific PNA invasion into DNA and DNA-guided cleavage of the displaced ssDNA strand by pAgo. Our *in vitro* PNA specificity assays demonstrated that PNA–target mismatches at the 3′ and 5′ ends were well tolerated up to 5 and 7 nt, respectively. This result indicated that PNA molecules can invade targets of partial complementarity with reasonable efficiency, as long as they are located at the PNA terminus. However, invasion efficiency was impaired if the mismatched region was located in the center of the PNA–DNA duplex and further decreased for nonconsecutive mismatches. DNA guides employed by pAgos ensure a secondary layer of specificity in our concept. The activity of PNP editors dropped substantially when we used gDNAs that were mismatched at the precise positions corresponding to the PNA invasion site, demonstrating that any activity at nonspecific sites in the genome would be extremely inefficient. In addition, DSB generation *in vivo* requires PNA invasion and pAgo cleavage to happen within a narrow window, further reducing the chances of off-target activity.

This work provides an additional alternative to other genome-editing technologies, including CRISPR/Cas-mediated genome modification, and may help overcome key challenges for CRISPR/Cas-based clinical applications. The diversity of the pAgo protein family and the practicality of PNA synthesis open up myriad applications for gene therapy and for targeted genome editing in diverse species. Moreover, there are several advantages of using PNP editors for genome editing. For instance, unlike Cas proteins with PAM, PNP editors do not require any sequence motif as a prerequisite for binding and cleavage. PNP editors employ the pAgo enzyme, which possesses one RNase-H-like fold in the PIWI domain and can generate only a single nick. Therefore, the generation of DSBs requires two PNA binding events and the positioning of two guided pAgo complexes in close proximity, minimizing the chances for any off-target activity. PNP editors employ PNA oligonucleotide analogs and pAgo proteins of smaller size compared to Cas9; therefore, packaging and delivery into target cells is expected to be more efficient compared to the CRISPR/Cas systems. At present, organellar DNA can be modified only using TALENs and base editors fused to TALEs due to inefficient transport of crRNA (CRISPR RNA) through organellar membranes. A PNP editor programmed with short DNA guides that carry a much smaller charge than crRNA and PNAs decorated with mitochondrial targeting signal could potentially be used to edit organellar genomes.

Although these advantages make PNP editors a powerful genome-editing technology, key issues warrant future research and focus. These include the specificity of PNA invasion, solubility and activity at high ionic strength and high salt concentrations present in the context of cellular environments. Delivery of PNA into cells and the localization of PNA to the cell nucleus pose a challenge, although significant progress has been made by utilizing miniPEG modification and nanoparticles (29). Furthermore, detailed analysis of immunogenicity and cellular toxicity is expected to provide detailed design guidelines for the use of PNAs for gene editing *in vivo* in a clinical context (29,38–40). A couple of features of pAgo proteins may complicate their use in genome editing: (i) the nonspecific guide-independent cleavage and chopping activity required for guide acquisition in bacterial hosts; and (2) nonspecific loading of pAgo proteins with endogenous microRNA or degraded DNA fragments within the cells and short half-life of externally provided gDNA molecules. These issues may be overcome by using a guide-preloaded pAgo or by providing PT-modified gDNAs, followed by delivery of preformed pAgo–gDNA complexes by lipofection or nucleofection. The chromatin state may help protect the genome from chopping activity of guide-free pAgo proteins due to the presence of histones.

We hope that our work may inspire the development of next-generation PNA molecules that exhibit robust invasion and entry into the cell nucleus and maintain high specificity and high on-target invasion efficiency at low concentrations. Due to the versatility of PNP editors, we envision that current work will expand the use of other pAgo proteins capable of programmable DNA binding for genome-editing applications. Finally, we envision the therapeutic value of this PNP editor technology, as the system is highly specific and the size of the complexes is much smaller than that of CRISPR cargoes, with potentially improved specificity and immunogenicity and lower cellular toxicity.

## Supplementary Material

gkad655_Supplemental_File

## Data Availability

The data underlying this article are available in the article and in its online supplementary material.

## Notes

Present address: Ahmed Mahas, Department of Genetics, Harvard University, Boston, MA 02115, USA.
