# Supplementary material for: Programmable site-specific DNA double-strand breaks via PNA-assisted prokaryotic Argonautes
Source: Nucleic Acids Res. 2023 Aug 10;51(17):9491–506. doi: 10.1093/nar/gkad655 (PMC10516665; doi:10.1093/nar/gkad655)
Supplement: gkad655_Supplemental_File [file gkad655_supplemental_file.pdf]

SUPPLEMENTARY INFORMATION

Programmable Site-Specific DNA Double-Strand Breaks via PNA-assisted Prokaryotic Argonautes

Tin Marsic[1], Gundra Sivakrishna Rao[1], Qiaochu Wang[1], Rashid Aman[1], Ahmed Mahas[1,2], and Magdy Mahfouz[1] *

[1]Laboratory for Genome Engineering and Synthetic Biology, Division of Biological Sciences, 4700 King Abdullah University of Science and Technology, Thuwal 23955-6900, Saudi Arabia. [2]Current address: Department of Genetics, Harvard University, Boston, MA 02115 USA

Correspondence: Magdy M. Mahfouz (magdy.mahfouz@kaust.edu.sa)

Supplementary figures

A

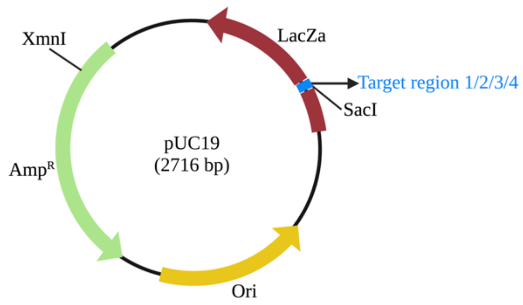

Circular pUC19 target plasmid containing different target regions	Target region specific for
1	γPNA1 / γPNA2
2	γPNA3 / γPNA4
3	γPNA2 / γPNA4
4	γPNA1 / γPNA3

B

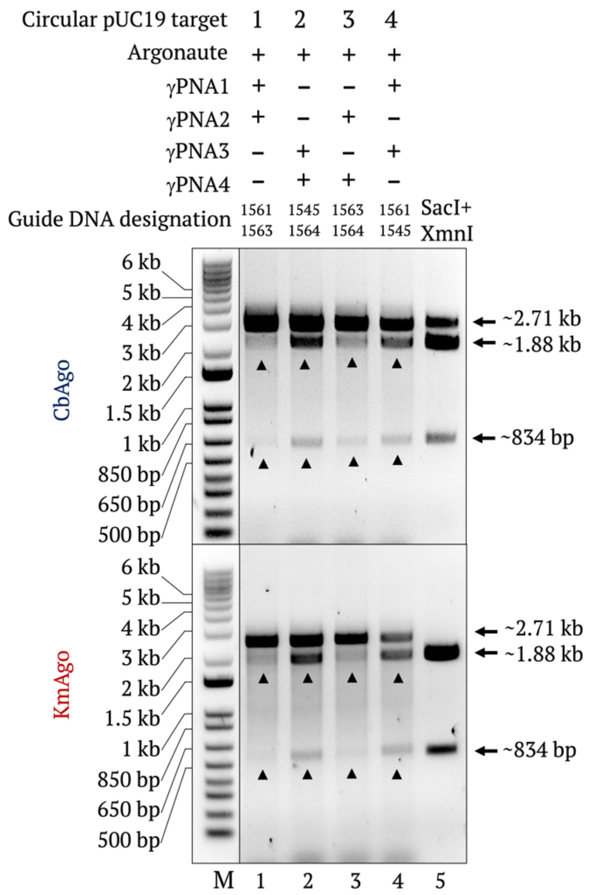

Supplementary Figure S1. Efficiency of different combinations of γPNA invasion and pAgo cleavage on circular plasmid. (**A**) Map of the pUC19 plasmid containing the different target regions. Target regions for binding of γPNA1 and γPNA2, γPNA3 and γPNA4, γPNA2 and γPNA4, or γPNA1 and γPNA3 were individually cloned into pUC19 vector at the EcoRI and BamHI restriction sites. These plasmids were used for testing the efficiencies of different combinations of γPNAs for invasion and pAgo cleavage. (**B**) Representative gel image showing the pAgo (upper

gel, CbAgo; lower gel, KmAgo) cleavage of pUC19 target plasmids invaded with γPNA1 and γPNA2 (Lane 1), γPNA3 and γPNA4 (Lane 2), γPNA2 and γPNA4 (Lane 3), or γPNA1and γPNA3 (Lane 4) using the corresponding guide combinations. All invaded samples were incubated with pAgo proteins for 1 h at 37°C and treated with XmnI restriction enzyme for 30 min at 37°C followed by proteinase K treatment and separation on a 0.9% (w/v) agarose gel. A SacI/XmnI digested (non-invaded) (Lane 5) sample was used as size control. Lane M represents the 1-kb plus DNA ladder. This experiment was run together with the experiment shown in Supplementary Figure S4; gels were later cropped but the same DNA ladder was retained.

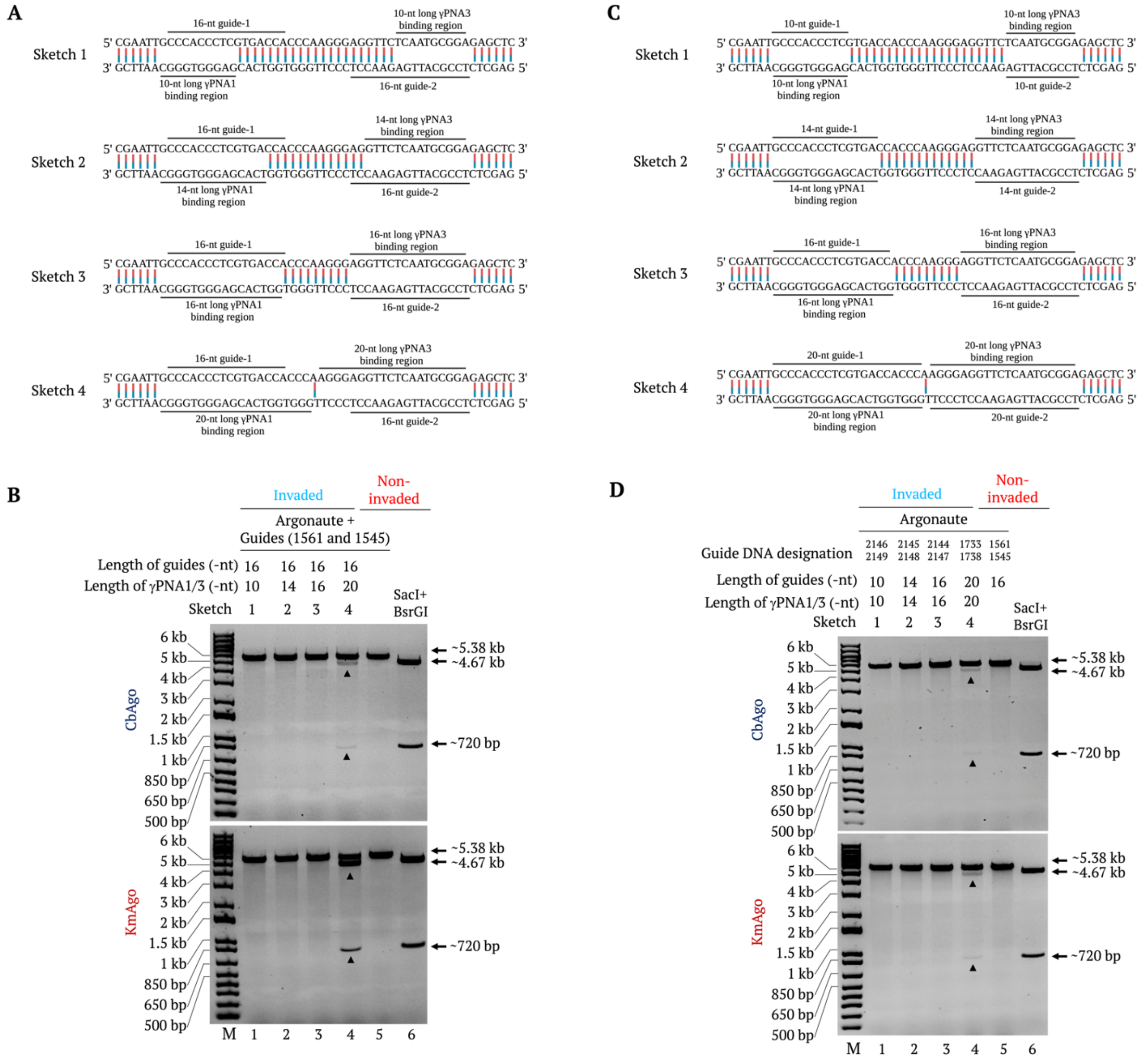

Supplementary Figure S2. Cleavage of BsrGI-linearized pMRS target invaded with γPNA1 and γPNA3 sequences of different lengths. (**A**) Schematic diagram showing the invasion of γPNA1 and γPNA3 sequences of different lengths with binding of the 16-nt guide DNA. Sketches 1–4 illustrate target DNA invaded with 10-, 14-, 16-, or 20-nt-long γPNA1 and γPNA3 molecules, respectively, with a 16-nt-long guide DNA binding to the target DNA. (**B**) Representative gel images showing the cleavage of the target DNA invaded by γPNA1 and γPNA3 of 10 nt (Lane 1), 14 nt (Lane 2), 16 nt (Lane 3), or 20 nt (Lane 4) in the presence of pAgo loaded with the 16-nt guide DNA (upper gel, CbAgo; lower gel, KmAgo). Non-invaded target cleavage with pAgo (Lane 5) and SacI-digested (non-invaded) (Lane 6) samples were included as controls. (**C**) Schematic diagram showing the invasion of γPNA1 and γPNA3 of different lengths and the binding of a PNA guide DNA molecule of the same length. Sketches 1–4 illustrate target DNA invaded with 10-, 14-, 16-, or 20-nt-long γPNA1 and γPNA3 molecules, respectively, with the corresponding 10-, 14-, 16-, or 20-nt-long guide DNA. (**D**) Representative gel images showing the cleavage of the target DNA invaded by γPNA1 and γPNA3 of 10, 14, 16, or 20 nt in length using pAgos (upper gel, CbAgo; lower gel, KmAgo) loaded with 10-nt (Lane 1), 14-nt (Lane 2), 16-nt (Lane 3), or 20-nt (Lane 4) guide DNA molecules, respectively. Non-invaded target cleavage with pAgo (Lane 5) and SacI-digested (non-invaded) (Lane 6) samples were included as controls. Lane M represents the 1-kb plus DNA ladder.

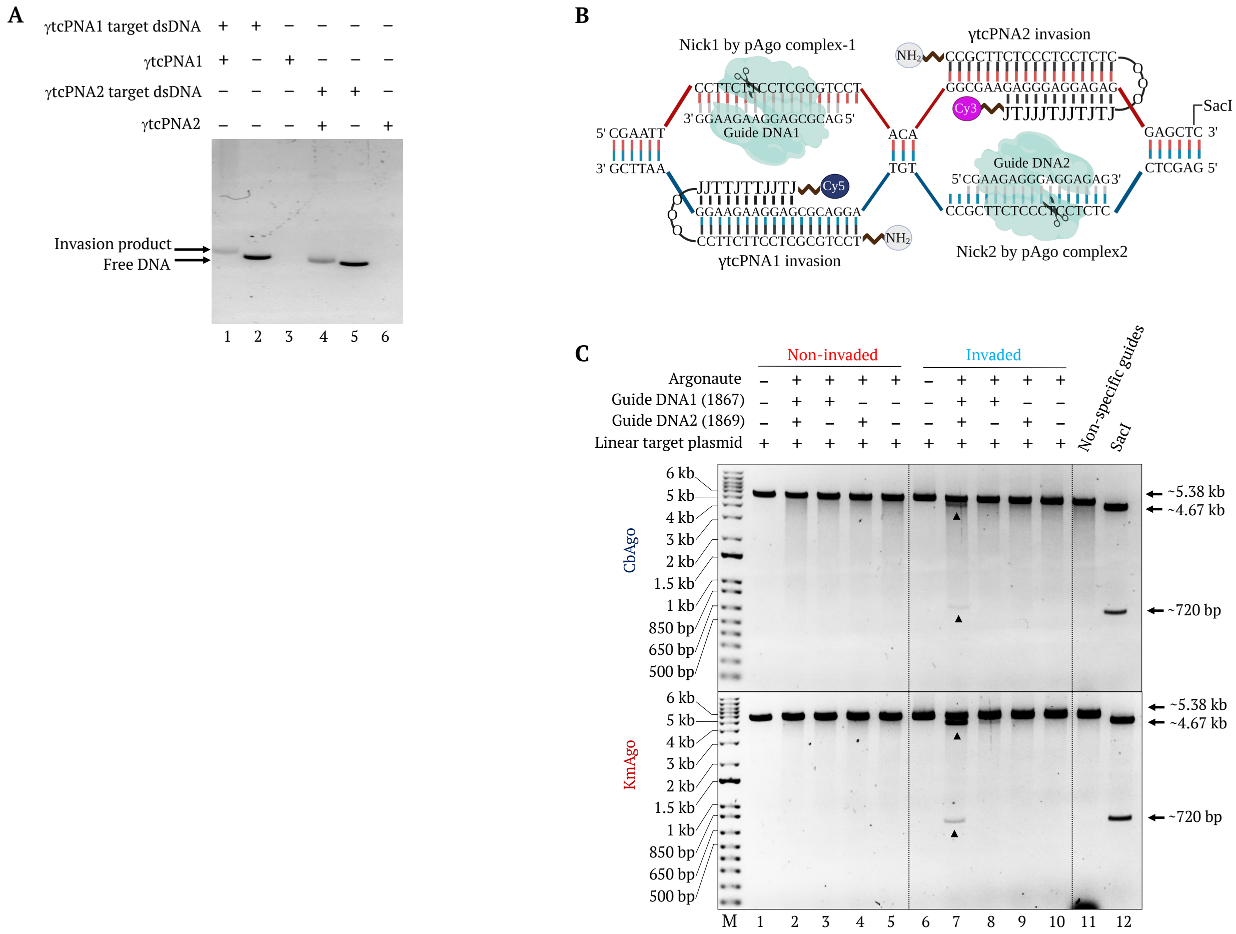

Supplementary Figure S3. pAgo-mediated cleavage of linear dsDNA invaded with γtcPNA. (**A**) Mobility shift assay of DNA invaded by γtcPNA1 and γtcPNA2 based on 2% (w/v) agarose gel electrophoresis. Lanes 1 and 4 are the DNA template invaded with γtcPNA1- or γtcPNA2-invaded DNA, respectively; Lanes 2 and 5 are non-invaded DNA; Lanes 3 and 4 are γtcPNA1 and γtcPNA2 only, respectively. (**B**) Schematic diagram of pAgo-mediated cleavage of the target region in the pMRS-γtcPNA+γtcPNA2 plasmid invaded by γtcPNA1 and γtcPNA2. γtcPNA1 and γtcPNA2 are respectively labeled with Cy5 and Cy3, although the labels are not relevant to the current manuscript. (**C**) Representative gel images showing pAgo-mediated (upper gel, CbAgo; lower gel, KmAgo) cleavage of a target linearized by BsrGI digest and invaded with γtcPNA1 and γtcPNA2. Lane 7, two pAgo-guide complexes; Lanes 8 and 9, single pAgo-guide complex; Lane 10, pAgo without guide; Lanes 1–5 show cleavage of the non-invaded target with different pAgo/guide combinations. pAgo cleavage with non-specific guides (Lane 11), and SacI-digested (non-invaded) (Lane 12) samples were included as controls. Lane M represents the 1-kb plus DNA ladder.

A

	52-nt	43-nt
ssDNA target :	5'-AATTAGCACTGCACGCCGTAGGTCTGGGTGGTCACGAGGGTGGGCATCCGCATTGAGAACCTCCCTAAGTCACAAAATCTGGATATAGTGAGCTC-3'	

B

Guide DNA designation	5'PT and 5'P guide sequences	Guide DNA designation	3'PT guide sequences
1776	/5Phos/A*GGTTCTCAATGCGGA	1781	/5Phos/AGGTTCTCAATGCGG*A
1777	/5Phos/A*G*GTTCTCAATGCGGA	1782	/5Phos/AGGTTCTCAATGCG*G*A
1778	/5Phos/A*G*G*TTCTCAATGCGGA	1783	/5Phos/AGGTTCTCAATGC*G*G*A
1779	/5Phos/A*G*G*TTCTCAATGCGGA	1784	/5Phos/AGGTTCTCAATG*C*G*G*A
1780	/5Phos/A*G*G*T*TCTCAATGCGGA	1785	/5Phos/AGGTTCTCAAT*G*C*G*G*A
1884	/5Phos/A*G*G*T*T*C*T*C*A*A*T*G*C*G*G*A		
1545	/5Phos/AGGTTCTCAATGCGGA		

C

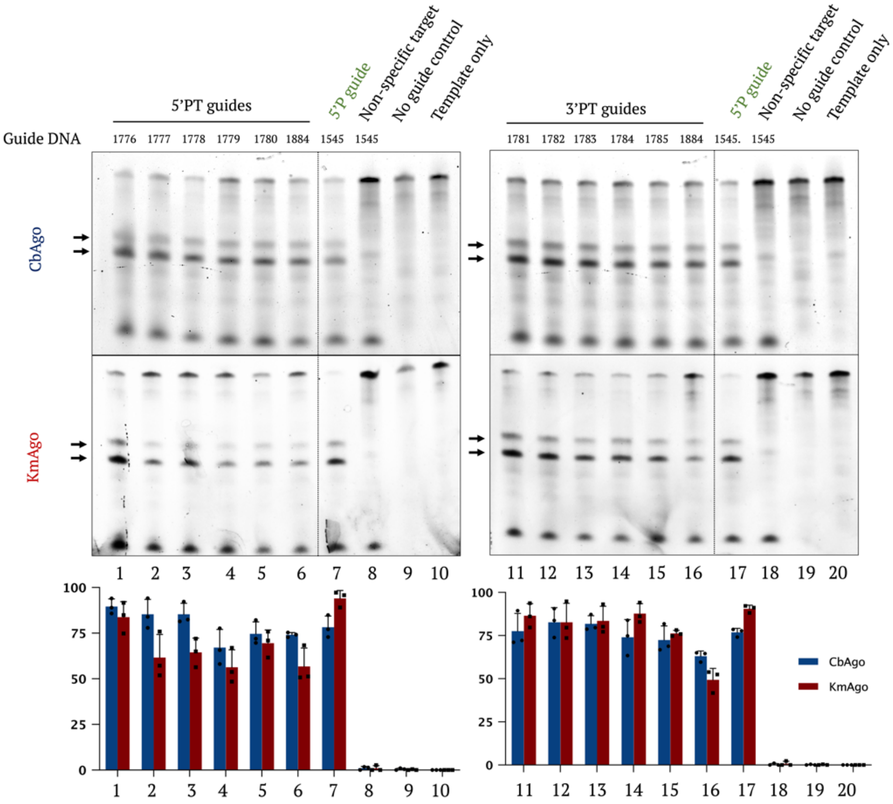

Supplementary Figure S4. pAgo-mediated cleavage of a ssDNA target using gDNAs with phosphorothioate bonds at various positions. (**A**) Sequence of the ssDNA target; the arrow indicates the pAgo cleavage position on the ssDNA molecule. (**B**) List of 5′- and 3′-phosphorothioated guides. Nucleobases with asterisks and highlighted in red indicate phosphorothioation. (**C**) ssDNA target (100 nM final concentration) was cleaved by pAgo complexes (upper panel, CbAgo; lower panel, KmAgo) (500 nM final concentration) pre-loaded with phosphorothioated gDNAs (200 nM final concentration) at 37°C for 1 h in 1X pAgo reaction buffer. Reaction products were resolved on 10% denaturing TBU gels and visualized by SYBR Gold staining. Lanes 1–5 are for 5′ PT guides; Lanes 11–15 are for 3′ PT guides; Lanes 6 and 16

used full PT-guides. Specific target with 5′ P guide without any PT (Lanes 7 and 17), non-specific target with 5′ P guide without any PT (Lanes 8 and 18), no guide control (Lanes 9 and 19), and template only (Lanes 10 and 20) were included as controls. Arrows indicate pAgo-cleaved products (upper panel, CbAgo; lower panel, KmAgo). Quantification values are shown as mean ± SD (n = 3).

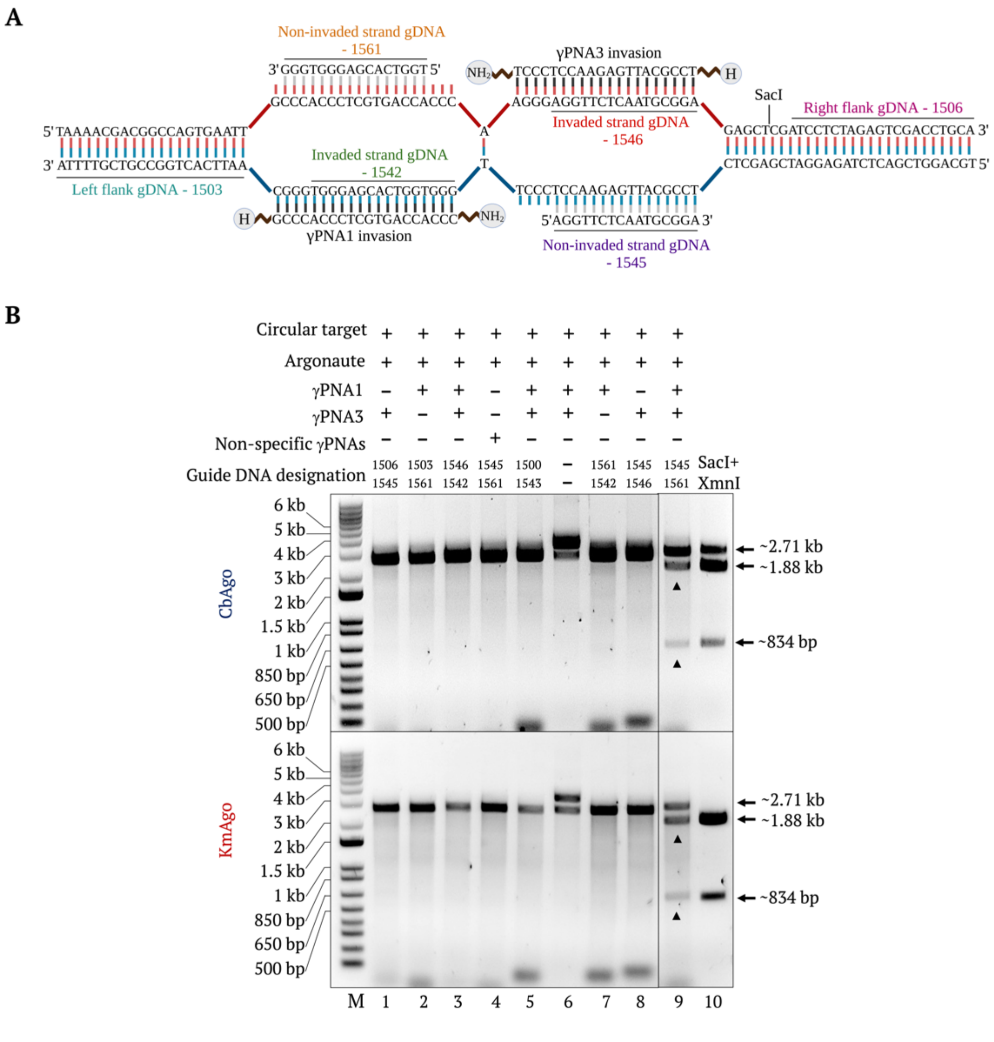

Supplementary Figure S5. pAgo-mediated targeting of flanking regions and invaded strands on PNA-invaded circular pUC19 plasmid. (**A**) Schematic diagram illustrating the invasion of the pUC19 target region by γPNA1 and γPNA3. Different guide binding regions are indicated in different colors. (**B**) Representative gel images showing the targeting of DNA targets invaded by one or two PNAs and the targeting of pAgo by different pairs of guides. Lane 1, plasmid invaded

by γPNA3 and incubated with a pair of pAgo-guide DNAs targeting the γPNA3-displaced ssDNA region and a guide targeting the right flanking, non-invaded region in immediate proximity to the invaded region. Lane 2, plasmid invaded by γPNA1 and incubated with a pair of pAgo-guide DNAs targeting the γPNA1-displaced ssDNA region and a guide targeting the left flanking, non-invaded region in immediate proximity to the invaded region. Lane 3, plasmid invaded by γPNA1 and γPNA3 and incubated with a pair of pAgo-guide DNAs targeting the γPNA-invaded strands. Lane 4, plasmid invaded by non-specific γPNAs targeted with pAgos loaded with specific guide DNAs. Lane 5, plasmid invaded by γPNA1 and γPNA3 targeted with pAgos loaded with non-specific guide DNA molecules. Lane 6, plasmid invaded by γPNA1 and γPNA3 targeted with pAgo lacking guide DNA. Lane 7, plasmid invaded by γPNA1 incubated with a pair of pAgo-guide complexes targeting the invaded strand and non-invaded strand opposite of each other. Lane 8, plasmid invaded by γPNA3 treated with a pair of pAgo-guide DNA complexes targeting the invaded strand and non-invaded strand opposite of each other. Lane 9, plasmid invaded by γPNA1 and γPNA3 incubated with a pair of pAgo-guide DNA complexes targeting the displaced ssDNA molecules. A SacI/XmnI-digested (non-invaded) (Lane 10) sample was used as size control. Lane M represents the 1-kb plus DNA ladder. This experiment was run together with Supplementary Figure S1; gels were later cropped with the same DNA ladder.

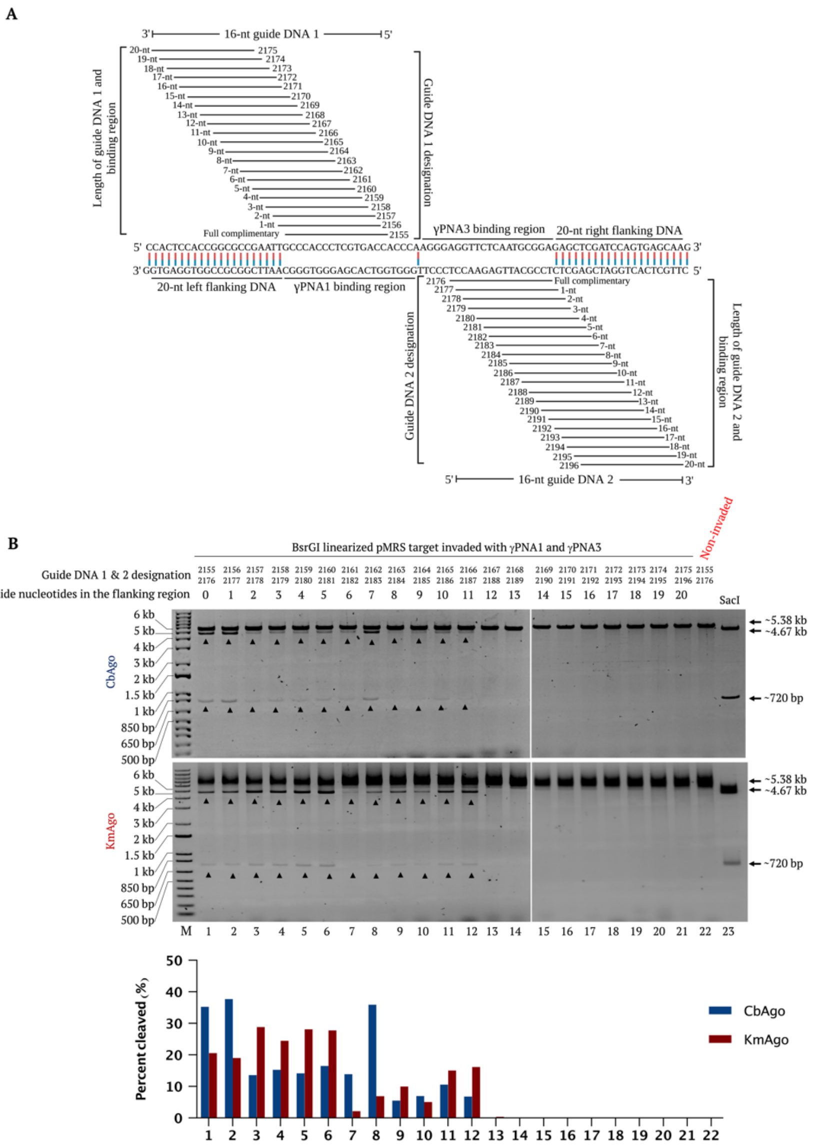

Supplementary Figure S6.

Targeting neighbouring dsDNA regions using the outward flanking guides. (**A**) Schematic diagram showing the binding sites of inward flanking guides 1 and 2 and the region of pMRS plasmid with 10-nt spacer invaded by γPNA1 and γPNA3. Flanking lengths ranged from 0 nt (full complementarity with the PNA-unwound ssDNA) to 10 nt, based on the number of nucleotides in the guide that target dsDNA regions. (**B**) Representative gel images showing pAgo-mediated (upper gel, CbAgo; lower gel, KmAgo) cleavage of the DNA target invaded by γPNA1 and γPNA3 using the different combinations of outward flanking guides (Lanes 1–11). Non-invaded target cleavage with pAgo (Lane 12) and SacI-digested (non-invaded) (Lane 13) samples were included as controls. Lane M represents the 1-kb plus DNA ladder.

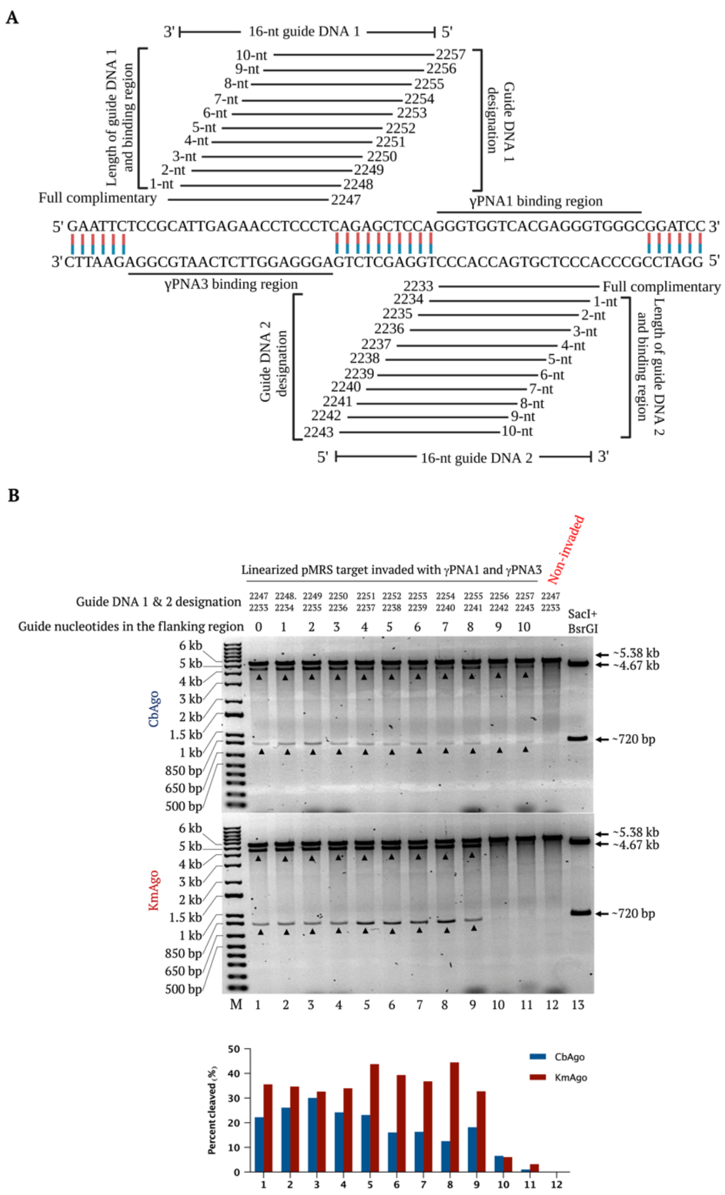

Supplementary Figure S7.

Targeting neighbouring dsDNA regions using the inward flanking guides. (**A**) Schematic diagram of the binding sites for outward flanking guides 1 and 2 and the regions invaded by γPNA1 and γPNA3 in the pMRS plasmid with 1-nt spacer. Flanking lengths range from 0 nt (full complementarity with the PNA-unwound ssDNA) to 20 nt (guide is targeting fully dsDNA region), based on the number of nucleotides in the guide that target dsDNA regions. (**B**) Representative gel images showing pAgo-mediated cleavage (upper gel, CbAgo; lower gel, KmAgo) of the DNA target invaded by γPNA1 and γPNA3 using the different combinations of inward flanking guides (Lanes 1–21). Non-invaded target cleavage using pAgo (Lane 22) and SacI-digested (non-invaded) (Lane 23) samples were included as controls. Lane M represents the 1-kb plus DNA ladder. All samples from this experiment were incubated at the same time. Different gels were used to run all the samples. Later, gel images were assembled together based on the DNA ladder.

A

	Guide mismatch regions	Anchor	Seed	Central	Supplementary	Tail
2-nt	Mismatch guide 1	3'GGGTGGGAGCACTGCA5'	3'GGGTGGGAGCAGAGGT5'	3'GGGTGGCTGCACTGGT5'	3'GGCATGGGAGCACTGGT5'	3'CCGTGGGAGCACTGGT5'
	Mismatch guide 2	5'TTGTTCTCAATGCGGA3'	5'AGGAACTCAATGCGGA3'	5'AGGTTCTCTTTGCGGA3'	5'AGGTTCTCAATGATGA3'	5'AGGTTCTCAATGCGTT3'
3-nt	Mismatch guide 1	3'GGGTGGGAGCACTCCA5'	3'GGGTGGGAGCTGAGGT5'	3'GGGTGGCTCCACTGGT5'	3'GGGACCGAGCACTGGT5'	3'CCCTGGGAGCACTGGT5'
	Mismatch guide 2	5'TCCTTCTCAATGCGGA3'	5'AGGAAGTCAATGCGGA3'	5'AGGTTCAGTATGCGGA3'	5'AGGTTCTCAAACGGGA3'	5'AGGTTCTCAATGCCCT3'
4-nt	Mismatch guide 1	3'GGGTGGGAGCACACCA5'	3'GGGTGGGAGGTGAGGT5'	3'GGGTGCCTCCACTGGT5'	3'GGGACCCAGCACTGGT5'	3'CCCAGGGAGCACTGGT5'
	Mismatch guide 2	5'TCCATCTCAATGCGGA3'	5'AGGAAGACAATGCGGA3'	5'AGGTTCAGTTTGCGGA3'	5'AGGTTCTCATACGGGA3'	5'AGGTTCTCAATGGCCT3'

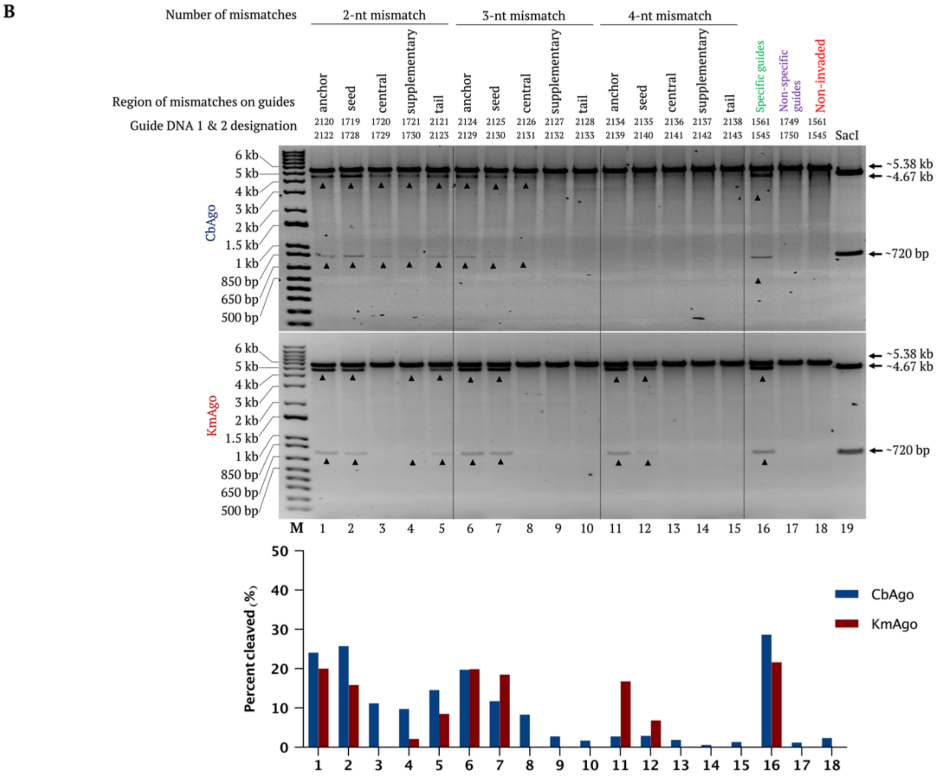

Supplementary Figure S8. Effect of number and position of mismatches in the guide DNA on pAgo cleavage efficiency. (**A**) Table summarizing the different mismatched guides 1 and 2, highlighting the region and number of mismatched nucleotides. (**B**) Representative gel images showing the pAgo-mediated cleavage of pMRS plasmid linearized by BsrGI digest and invaded by γPNA1 and γPNA3 using different mismatched guides. Cleavage of invaded plasmid with CbAgo (upper gel) and KmAgo (lower gel) pre-loaded with different guide DNA containing 2-nt mismatches (Lanes 1–5), 3-nt mismatches (Lanes 6–10), and 4-nt mismatches (Lanes 11–15) at different positions are shown. Samples invaded by γPNA1 and γPNA3 with specific guides and non-specific guides (Lanes 16 and 17, respectively) are included. Non-invaded but incubated with specific guides (Lane 18) and SacI-digested (non-invaded) (Lanes 19) samples are included as control reactions. Lane M represents the 1-kb plus DNA ladder.

A

γPNA1 sequence (5'-3')	5'GCCCACCCTCGTGACCACCC3'	γPNA3 sequence (3'-5')	3'TCCCTCCAAGAGTTACGCCT5'
γPNA1 complimentary template	3'CGGGTGGGAGCACTGGTGGG5'	γPNA3 complimentary template	5'AGGGAGGTTCTCAATGCGGA3'

No. of mismatches	Target mismatch at 3' end of γPNA1	Target mismatch at 3' end of γPNA3
1	3'CGGGTGGGAGCACTGGTGGC5'	5'TGGGAGGTTCTCAATGCGGA3'
2	3'CGGGTGGGAGCACTGGTGCC5'	5'TCGGAGGTTCTCAATGCGGA3'
3	3'CGGGTGGGAGCACTGGTCCC5'	5'TCCGAGGTTCTCAATGCGGA3'
4	3'CGGGTGGGAGCACTGGACCC5'	5'TCCCAGGTTCTCAATGCGGA3'
5	3'CGGGTGGGAGCACTGCACCC5'	5'TCCCTGGTTCTCAATGCGGA3'
7	3'CGGGTGGGAGCACACCACCC5'	5'TCCCTCCTTCTCAATGCGGA3'
9	3'CGGGTGGGAGCTGACCACCC5'	5'TCCCTCCAACTCAATGCGGA3'
11	3'CGGGTGGGACGTGACCACCC5'	5'TCCCTCCAAGACAATGCGGA3'

No. of mismatches	Target mismatch at central region	Target mismatch at central region
1	3'CGGGTGGTAGCACTGGTGGG5'	5'AGGGAGGTTATCAATGCGGA3'
2	3'CGGGTGGTTGCACTGGTGGG5'	5'AGGGAGGTTAACAATGCGGA3'
3	3'CGGGTGGTTCCACTGGTGGG5'	5'AGGGAGGTTAAGAATGCGGA3'
4	3'CGGGTGGTTCGACTGGTGGG5'	5'AGGGAGGTTAAGTATGCGGA3'

No. of mismatches	No-consecutive target mismatches	No-consecutive Target mismatches
2	3'CGGGTGGCACCACTGGTGGG5'	5'AGGGAGGTACACAATGCGGA3'
4	3'CGGGTCGCACCTCTGGTGGG5'	5'AGGGAGCTACACTATGCGGA3'
6	3'CGGCTCGCACCTCAGGTGGG5'	5'AGGGTGCTACACTAAGCGGA3'
8	3'CCGCTCGCACCTCAGCTGGG5'	5'AGCGTGCTACACTAAGGGGA3'

No. of mismatches	Target mismatch at 5' end of γPNA1	Target mismatch at 5' end of γPNA3
1	3'CCGGTGGGAGCACTGGTGGG5'	5'AGGGAGGTTCTCAATGCGGT3'
2	3'CCCGTGGGAGCACTGGTGGG5'	5'AGGGAGGTTCTCAATGCGCT3'
3	3'CCCCTGGGAGCACTGGTGGG5'	5'AGGGAGGTTCTCAATGCCCT3'
4	3'CCCCAGGGAGCACTGGTGGG5'	5'AGGGAGGTTCTCAATGGCCT3'
5	3'CCCCACGGAGCACTGGTGGG5'	5'AGGGAGGTTCTCAATCGCCT3'
7	3'CCCCACCCAGCACTGGTGGG5'	5'AGGGAGGTTCTCATACGCCT3'
9	3'CCCCACCCTCCACTGGTGGG5'	5'AGGGAGGTTCTGTTACGCCT3'
11	3'CCCCACCCTCGTCTGGTGGG5'	5'AGGGAGGTTGAGTTACGCCT3'

B

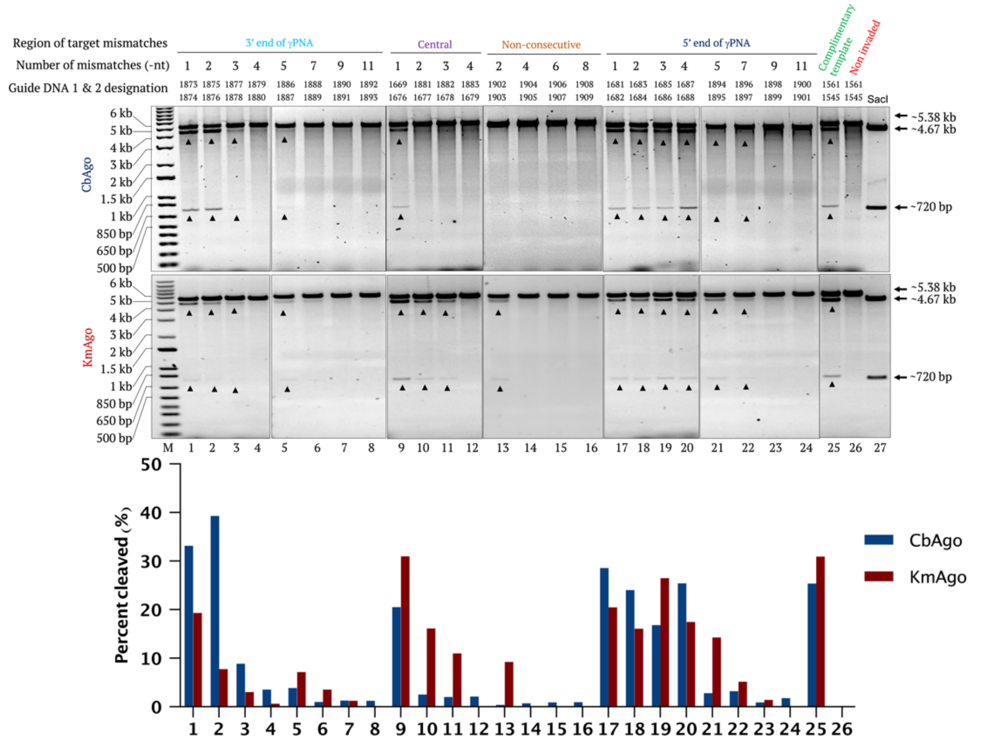

Supplementary Figure S9. Evaluating pAgo cleavage efficiency using the mismatch-specific guides on DNA templates targeted with mismatched γPNA molecules. **(A)** Table summarizing the γPNA1 and γPNA3 sequences and their mismatched target sequences. DNA target mismatches are represented as 3′ end mismatches to γPNA1 and γPNA3 (1- to 11-nt mutations), central mismatches to γPNA1 and γPNA3 (1- to 4-nt mutations), non-consecutive mismatches (2- to 8-nt mutations), and 5′ end mismatches to γPNA1 and γPNA3 (1- to 11-nt mutations). **(B)** Representative gel images showing the cleavage mismatched linear targets invaded by γPNA1 and γPNA3 using pAgo complexes (upper gel, CbAgo; lower gel, KmAgo) pre-loaded with mismatch-specific guides. Lanes 1–8 show the cleavage products of 3′ end mismatched targets, Lanes 9–12 show the cleavage of central mismatched targets, Lanes 13–16 show the cleavage of non-consecutive mismatched targets, and Lanes 17–24 show the cleavage of 5′ end mismatched targets using the mismatch-specific guides. Lane 25 is the correct target DNA invaded with γPNA1 and γPNA3 and cleavage with pAgo loaded with specific guide DNA molecules. Non-invaded samples incubated with specific guides and pAgo (Lane 26), and SacI-digested (non-invaded) (Lane 27) samples were used as control reactions. Lane M represents the 1-kb plus DNA ladder. All samples from this experiment were incubated at the same time. Different gels were used to run all the samples. Later, gel images were assembled together based on the DNA ladder.

A

γPNA1 sequence (5'-3')	5'GCCCACCCTCGTGACCACCC3'	γPNA3 sequence (3'-5')	3'TCCCTCCAAGAGTTACGCCT5'
γPNA1 complimentary template	3'CGGGTGGGAGCACTGGTGGG5'	γPNA3 complimentary template	5'AGGGAGGTTCTCAATGCGGA3'

No. of mismatches	Target mismatch at 3' end of γPNA1	Target mismatch at 3' end of γPNA3
1	3'CGGGTGGGAGCACTGGTGGC5'	5'TGGGAGGTTCTCAATGCGGA3'
2	3'CGGGTGGGAGCACTGGTGCC5'	5'TCGGAGGTTCTCAATGCGGA3'
3	3'CGGGTGGGAGCACTGGTCCC5'	5'TCCGAGGTTCTCAATGCGGA3'
4	3'CGGGTGGGAGCACTGGACCC5'	5'TCCCAGGTTCTCAATGCGGA3'
5	3'CGGGTGGGAGCACTGCACCC5'	5'TCCCTGGTTCTCAATGCGGA3'
7	3'CGGGTGGGAGCACACCACCC5'	5'TCCCTCCTTCTCAATGCGGA3'
9	3'CGGGTGGGAGCTGACCACCC5'	5'TCCCTCCAACTCAATGCGGA3'
11	3'CGGGTGGGACGTGACCACCC5'	5'TCCCTCCAAGACAATGCGGA3'

No. of mismatches	Target mismatch at central region	Target mismatch at central region
1	3'CGGGTGGTAGCACTGGTGGG5'	5'AGGGAGGTTATCAATGCGGA3'
2	3'CGGGTGGTTGCACTGGTGGG5'	5'AGGGAGGTTAACAATGCGGA3'
3	3'CGGGTGGTTCCACTGGTGGG5'	5'AGGGAGGTTAAGAATGCGGA3'
4	3'CGGGTGGTTCGACTGGTGGG5'	5'AGGGAGGTTAAGTATGCGGA3'

No. of mismatches	No-consecutive target mismatches	No-consecutive Target mismatches
2	3'CGGGTGGCACCACTGGTGGG5'	5'AGGGAGGTACACAATGCGGA3'
4	3'CGGGTCGCACCTCTGGTGGG5'	5'AGGGAGCTACACTATGCGGA3'
6	3'CGGCTCGCACCTCAGGTGGG5'	5'AGGGTGCTACACTAAGCGGA3'
8	3'CCGCTCGCACCTCAGCTGGG5'	5'AGCGTGCTACACTAAGGGGA3'

No. of mismatches	Target mismatch at 5' end of γPNA1	Target mismatch at 5' end of γPNA3
1	3'CCGGTGGGAGCACTGGTGGG5'	5'AGGGAGGTTCTCAATGCGGT3'
2	3'CCCGTGGGAGCACTGGTGGG5'	5'AGGGAGGTTCTCAATGCGCT3'
3	3'CCCCTGGGAGCACTGGTGGG5'	5'AGGGAGGTTCTCAATGCCCT3'
4	3'CCCCAGGGAGCACTGGTGGG5'	5'AGGGAGGTTCTCAATGGCCT3'
5	3'CCCCACGGAGCACTGGTGGG5'	5'AGGGAGGTTCTCAATCGCCT3'
7	3'CCCCACCCAGCACTGGTGGG5'	5'AGGGAGGTTCTCATACGCCT3'
9	3'CCCCACCCTCCACTGGTGGG5'	5'AGGGAGGTTCTGTTACGCCT3'
11	3'CCCCACCCTCGTCTGGTGGG5'	5'AGGGAGGTTGAGTTACGCCT3'

B

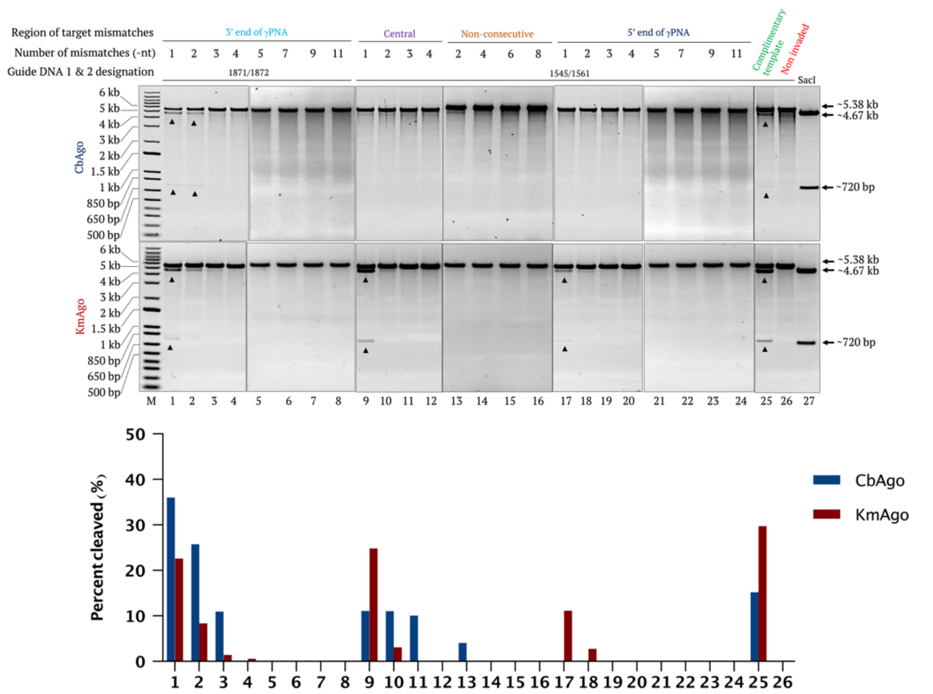

Supplementary Figure S10. Evaluating pAgo cleavage efficiency using the mismatched guides on DNA templates targeted with mismatched γPNA molecules. **(A)** Table summarizing the sequences of γPNA1 and γPNA3 and corresponding mismatched targets. DNA target mismatches are represented as 3′ end mismatches to γPNA1 and γPNA3 (1- to 11-nt mutations), central mismatches to γPNA1 and γPNA3 (1- to 4-nt mutations), non-consecutive mismatches (2- to 8-nt mutations), and 5′ end mismatches to γPNA1 and γPNA3 (1- to 11-nt mutations). **(B)** Representative gel images showing pAgo-mediated cleavage (upper gel, CbAgo; lower gel, KmAgo) of mismatched linear targets invaded by γPNA1 and γPNA3 with partial specific guides. Lanes 1–8, cleavage products of 3′ end mismatched targets; Lanes 9–12, cleavage products of central mismatched targets; Lanes 13–16, cleavage products of non-consecutive mismatched targets; and Lanes 17–24, cleavage products of 5′ end mismatched targets using partial specific guides. Lane 25 is the correct target DNA invaded with γPNA1 and γPNA3 showing its cleavage products with pAgo complexes loaded with specific guide DNA molecules. Non-invaded samples incubated with specific guides and pAgo (Lane 26), and SacI-digested (non-invaded) (Lane 27) samples were used as control reactions. Lane M represents the 1-kb plus DNA ladder. All samples

from this experiment were incubated at the same time. Different gels were used to run all the samples. Later, gel images were assembled together based on the DNA ladder.

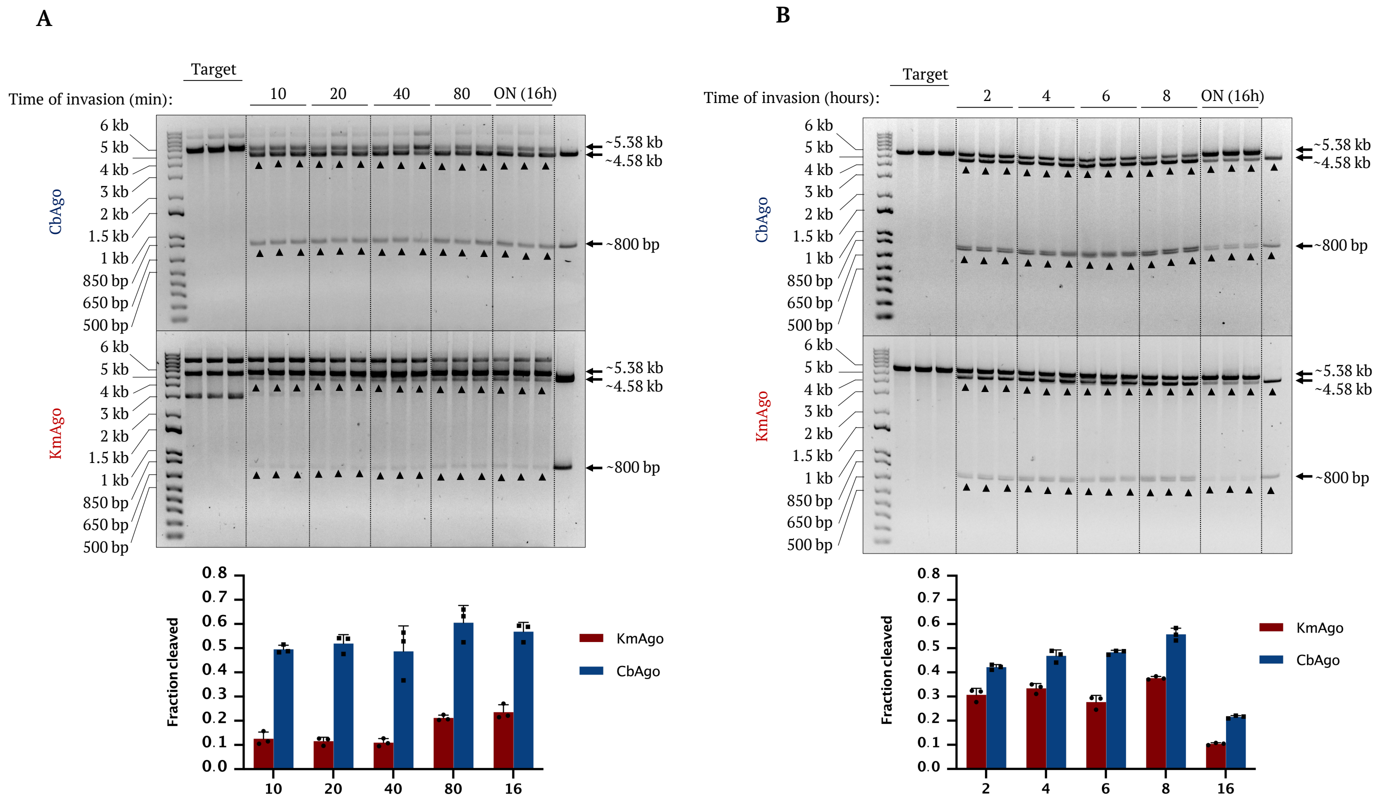

Supplementary Figure S11. Evaluating γPNA invasion time on PNP editor efficiency. **(A)** γPNA invasion time course performed on target circular plasmid (pMRS multiplexing plasmid). Circular pMRS plasmid (20 ng/μL final) was invaded with 100 nM (final) γPNA5 and γPNA6 for 10, 20, 40, 80 minutes and 16h over night at 37°C in total volume of 35 μL. Invaded plasmid (80 ng total) was cleaved with CbAgo and KmAgo for 1h and 30 minutes at 37°C and further restricted with AgeI for 30 minutes at 37°C. Quantification values are shown as mean ± SD (n = 3). **(B)** γPNA invasion time course performed on SacI linearized pMRS plasmid (pMRS multiplexing plasmid). Linearized pMRS plasmid (20 ng/μL final) was invaded with 100 nM (final) γPNA5 and γPNA6 for 2, 4, 6, 8 and 16h at 37°C. Invaded linear plasmid (80 ng total) was cleaved with CbAgo and KmAgo for 1h and 30 minutes at 37°C. Quantification values are shown as mean ± SD (n = 3).

Supplementary files

1) Expression vectors for pAgo purification used in this study

A. CbAgo-Twin-Strep-SUMO

tttcctgtttggtcactgatgcctccgtgtaagggggatttctgttcatgggggtaatgataccgatgaa
acgagagaggatgctcacgatacgggttactgatgatgaacatgcccggttactggaacgttgtgagggt
aaacaactggcggtatggatgcggcgggaccagagaaaaatcactcagggtcaatgccagcgcttcgtta
atacagatgtaggtgttccacagggtagccagcagcatcctgcgatgcagatccggaacataatggtgca
gggcgctgacttccgcgtttccagactttacgaaacacggaaaccgaagaccattcatgttgttgctcag
gtcgcagacgttttgcagcagcagtcgcttcacgttcgctcgcgtatcggtgattcattctgctaaccag
taaggcaaccccgccagcctagccgggtcctcaacgacaggagcacgatcatgcgcacccgtggccagga
cccaacgctgcccgagatgcgccgcgtgcggctgctggagatggcggacgcgatggatatgttctgccaa
gggttggtttgcgcattcacagttctccgcaagaattgattggctccaattcttggagtggtgaatccgt
tagcgaggtgccgccggcttccattcaggtcgaggtggcccggctccatgcaccgcgacgcaacgcgggg
aggcagacaaggtatagggcggcgcctacaatccatgccaacccgttccatgtgctcgccgaggcggcat
aaatcgccgtgacgatcagcggtccaatgatcgaagttaggctggtaagagccgcgagcgatccttgaag
ctgtccctgatggtcgtcatctacctgcctggacagcatggcctgcaacgcgggcatcccgatgccgccg
gaagcgagaagaatcataatggggaaggccatccagcctcgcgtcgcgaacgccagcaagacgtagccca
gcgcgtcggccgccatgccggcgataatggcctgcttctcgccgaaacgtttggtggcgggaccagtgac
gaaggcttgagcgagggcgtgcaagattccgaataccgcaagcgacaggccgatcatcgtcgcgctccag
cgaaagcggtcctcgccgaaaatgacccagagcgctgccggcacctgtcctacgagttgcatgataaaga
agacagtcataagtgcggcgacgatagtcatgccccgcgcccaccggaaggagctgactgggttgaaggc
tctcaagggcatcggtcgagatcccggtgcctaatgagtgagctaacttacattaattgcgttgcgctca
ctgcccgctttccagtcgggaaacctgtcgtgccagctgcattaatgaatcggccaacgcgcggggagag
gcggtttgcgtattgggcgccagggtggtttttcttttcaccagtgagacgggcaacagctgattgccct
tcaccgcctggccctgagagagttgcagcaagcggtccacgctggtttgccccagcaggcgaaaatcctg
tttgatggtggttaacggcgggatataacatgagctgtcttcggtatcgtcgtatcccactaccgagata
tccgcaccaacgcgcagcccggactcggtaatggcgcgcattgcgcccagcgccatctgatcgttggcaa
ccagcatcgcagtgggaacgatgccctcattcagcatttgcatggtttgttgaaaaccggacatggcact
ccagtcgccttcccgttccgctatcggctgaatttgattgcgagtgagatatttatgccagccagccaga
cgcagacgcgccgagacagaacttaatgggcccgctaacagcgcgatttgctggtgacccaatgcgacca
gatgctccacgcccagtcgcgtaccgtcttcatgggagaaaataatactgttgatgggtgtctggtcaga
gacatcaagaaataacgccggaacattagtgcaggcagcttccacagcaatggcatcctggtcatccagc
ggatagttaatgatcagcccactgacgcgttgcgcgagaagattgtgcaccgccgctttacaggcttcga
cgccgcttcgttctaccatcgacaccaccacgctggcacccagttgatcggcgcgagatttaatcgccgc
gacaatttgcgacggcgcgtgcagggccagactggaggtggcaacgccaatcagcaacgactgtttgccc
gccagttgttgtgccacgcggttgggaatgtaattcagctccgccatcgccgcttccactttttcccgcg
ttttcgcagaaacgtggctggcctggttcaccacgcgggaaacggtctgataagagacaccggcatactc
tgcgacatcgtataacgttactggtttcacattcaccaccctgaattgactctcttccgggcgctatcat
gccataccgcgaaaggttttgcgccattcgatggtgtccgggatctcgacgctctcccttatgcgactcc
tgcattaggaagcagcccagtagtaggttgaggccgttgagcaccgccgccgcaaggaatggtgcatgca
aggagatggcgcccaacagtcccccggccacggggcctgccaccatacccacgccgaaacaagcgctcat
gagcccgaagtggcgagcccgatcttccccatcggtgatgtcggcgatataggcgccagcaaccgcacct
gtggcgccggtgatgccggccacgatgcgtccggcgtagaggatcgagatctcgatcccgcgaaattaat
acgactcactataggggaattgtgagcggataacaattcccctctagaaataattttgtttaactttaag
aaggagatataccatgggcagcagccatcatcatcatcatcacagcagcggcctggtgccgcgcggcagc
catatggctagctggagccatccgcagtttgaaaaaggtggtggtagcggtggtggttcaggtggtagtg
catggtcacaccctcagtttgagaaaatgtcggactcagaagtcaatcaagaagctaagccagaggtcaa
gccagaagtcaagcctgagactcacatcaatttaaaggtgtccgatggatcttcagagatcttcttcaag
atcaaaaagaccactcctttaagaaggctgatggaagcgttcgctaaaagacagggtaaggaaatggact
ccttaagattcttgtacgacggtattagaatccaagctgatcagacccctgaagatttggacatggagga
taacgatattattgaggctcacagagaacagattggtggatccATGAATAACCTGACTTTTGAAGCATTT
GAAGGTATCGGTCAATTAAATGAATTAAACTTCTATAAATACCGCTTGATTGGCAAAGGGCAAATTGACA
ATGTACACCAAGCAATTTGGTCCGTCAAATATAAACTGCAAGCTAACAATTTCTTCAAGCCAGTTTTTGT
CAAAGGTGAAATCCTGTATAGCCTGGACGAACTTAAGGTAATTCCCGAATTTGAAAACGTTGAGGTGATC
CTTGATGGCAATATCATCCTTAGCATTTCTGAGAACACCGATATTTACAAAGATGTAATCGTTTTTACA

TTAACAACGCACTGAAAAACATTAAAGACATCACTAACTACCGCAAATACATCACTAAAAATACCGACGA
AATCATCTGCAAGAGCATTTTGACCACCAATTTAAAATATCAATATATGAAAAGTGAGAAAGGTTTTAAA
CTGCAGCGTAAATTTAAAATCTCTCCAGTTGTCTTTCGTAATGGAAAGGTTATTCTGTATTTGAATTGTA
GTTCCGATTTTAGTACCGACAAAAGTATTTATGAAATGCTGAACAATGGCCTGGACGTAGTCGGTCTTCA
AGTAAAAAACCGCTGGACAAACTCAAACGGCAACATTTTTATCGAAGAAGTGTTAGACAAGAGCATTAGC
GAACCAGGAACGAGTGGAAAACTGGGCCAATCTCTCATTGACTACTACATTAATGGAAACCAAAAATATC
GTGTGGAGAAATTTACTGATGAAGATAAAAAAGCCAAAGTGATCAAAGCTAAAATTAAAAACAAAACCTA
TAACTACATCCCGCAGGCCCTTACTCCCGTCATCACCCGTGAATACTTGTCACACACCGATAAAAAGTTT
AGCAAACAGATTGAAAACGTTATCAAAATGGACATGAACTATCGTTACCAGACCCTGAAGTCGTTTGTCG
AAGATATTGGGGTCATCAAAGAACTTAACAACTTACACTTCAAAAACCAATACTACACGAATTTTGATTT
TATGGGTTTTGAAAGCGGAATCTTAGAGGAACCTGTTCTGATGGGAGCAAACGGTAAAATTAAAGACAAA
AAACAGATTTTTATTAACGGTTTTTTCAAGAACCCAAAAGAAAACGTCAAATTTGGTGTGCTCTATCCCG
AAGGATGTATGGAAAACGCCCAATCAATTGCGCGTAGCATTCTTGATTTTGCAACTGCTGGTAAATACAA
TAAACAGGAAAATAAATACATTTCCAAAAACCTTATGAACATCGGCTTCAAACCATCTGAATGTATCTTT
GAATCTTACAAGCTGGGTGACATTACTGAGTACAAAGCGACTGCACGTAAACTGAAAGAACACGAAAAGG
TTGGGTTTGTGATTGCAGTTATCCCAGATATGAATGAATCCGAAGTCGAAAATCCATACAATCCCTTTAA
GAAAGTCTGGGCCAAACTTAACATCCCGAGCCAGATGATTACGCTTAAGACAACCGAAAAATTTAAAAAC
ATCGTGGACAAAAGTGGACTTTACTATTTGCATAATATTGCTTTGAATATCCTGGGTAAAATTGGAGGTA
TCCCGTGGATTATTAAAGACATGCCAGGTAATATCGACTGTTTCATTGGCTTAGATGTCGGCACCCGCGA
AAAAGGCATTCATTTCCCTGCGTGCTCTGTCCTGTTCGATAAATACGGCAAACTGATTAATTACTATAAA
CCCACAATTCCCCAATCTGGCGAAAAGATTGCAGAAACCATTCTTCAAGAAATCTTTGATAACGTCCTGA
TTTCATATAAAGAGGAAAATGGCGAGTACCCGAAGAATATTGTAATTCACCGTGATGGATTCAGCCGTGA
AAACATTGACTGGTATAAAGAGTATTTCGACAAGAAAGGTATCAAATTCAACATCATCGAAGTCAAAAAA
AATATCCCTGTTAAAATCGCTAAAGTTGTCGGCTCTAACATTTGCAATCCAATTAAAGGCTCCTATGTCC
TGAAAAACGATAAAGCATTTATTGTAACCACCGATATTAAAGACGGCGTGGCCAGCCCTAACCCACTTAA
AATTGAAAAAACGTACGGTGACGTGGAAATGAAATCCATCCTTGAACAAATCTACTCCCTGAGCCAGATT
CACGTCGGTAGCACCAAAAGTCTCCGCTTACCCATTACCACTGGCTATGCTGATAAAATTTGCAAAGCAA
TCGAATATATTCCCCAAGGCGTTGTGGATAACCGCCTCTTCTTTCTGtgaGCggccgcactcgaggcccg
aaaggaagctgagttggctgctgccaccgctgagcaataactagcataaccccttggggcctctaaacgg
gtcttgaggggttttttgctgaaaggaggaactatatccggatatcccgcaagaggcccggcagtaccgg
cataaccaagcctatgcctacagcatccagggtgacggtgccgaggatgacgatgagcgcattgttagat
ttcatacacggtgcctgactgcgttagcaatttaactgtgataaactaccgcattaaagcttatcgatga
taagctgtcaaacatgagaattcttgaagacgaaagggcctcgtgatacgcctatttttataggttaatg
tcatgataataatggtttcttagacgtcaggtggcacttttcggggaaatgtgcgcggaacccctatttg
tttatttttctaaatacattcaaatatgtatccgctcatgagacaataaccctgataaatgcttcaataa
tattgaaaaaggaagagtatgagtattcaacatttccgtgtcgcccttattcccttttttgcggcatttt
gccttcctgtttttgctcacccagaaacgctggtgaaagtaaaagatgctgaagatcagttgggtgcacg
agtgggttacatcgaactggatctcaacagcggtaagatccttgagagttttcgccccgaagaacgtttt
ccaatgatgagcacttttaaagttctgctatgtggcgcggtattatcccgtgttgacgccgggcaagagc
aactcggtcgccgcatacactattctcagaatgacttggttgagtactcaccagtcacagaaaagcatct
tacggatggcatgacagtaagagaattatgcagtgctgccataaccatgagtgataacactgcggccaac
ttacttctgacaacgatcggaggaccgaaggagctaaccgcttttttgcacaacatgggggatcatgtaa
ctcgccttgatcgttgggaaccggagctgaatgaagccataccaaacgacgagcgtgacaccacgatgcc
tgcagcaatggcaacaacgttgcgcaaactattaactggcgaactacttactctagcttcccggcaacaa
ttaatagactggatggaggcggataaagttgcaggaccacttctgcgctcggcccttccggctggctggt
ttattgctgataaatctggagccggtgagcgtgggtctcgcggtatcattgcagcactggggccagatgg
taagccctcccgtatcgtagttatctacacgacggggagtcaggcaactatggatgaacgaaatagacag
atcgctgagataggtgcctcactgattaagcattggtaactgtcagaccaagtttactcatatatacttt
agattgatttaaaacttcatttttaatttaaaaggatctaggtgaagatcctttttgataatctcatgac
caaaatcccttaacgtgagttttcgttccactgagcgtcagaccccgtagaaaagatcaaaggatcttct
tgagatcctttttttctgcgcgtaatctgctgcttgcaaacaaaaaaaccaccgctaccagcggtggttt
gtttgccggatcaagagctaccaactctttttccgaaggtaactggcttcagcagagcgcagataccaaa

tactgtccttctagtgtagccgtagttaggccaccacttcaagaactctgtagcaccgcctacatacctc
gctctgctaatcctgttaccagtggctgctgccagtggcgataagtcgtgtcttaccgggttggactcaa
gacgatagttaccggataaggcgcagcggtcgggctgaacggggggttcgtgcacacagcccagcttgga
gcgaacgacctacaccgaactgagatacctacagcgtgagctatgagaaagcgccacgcttcccgaaggg
agaaaggcggacaggtatccggtaagcggcagggtcggaacaggagagcgcacgagggagcttccagggg
gaaacgcctggtatctttatagtcctgtcgggtttcgccacctctgacttgagcgtcgatttttgtgatg
ctcgtcaggggggcggagcctatggaaaaacgccagcaacgcggcctttttacggttcctggccttttgc
tggccttttgctcacatgttctttcctgcgttatcccctgattctgtggataaccgtattaccgcctttg
agtgagctgataccgctcgccgcagccgaacgaccgagcgcagcgagtcagtgagcgaggaagcggaaga
gcgcctgatgcggtattttctccttacgcatctgtgcggtatttcacaccgcaatggtgcactctcagta
caatctgctctgatgccgcatagttaagccagtatacactccgctatcgctacgtgactgggtcatggct
gcgccccgacacccgccaacacccgctgacgcgccctgacgggcttgtctgctcccggcatccgcttaca
gacaagctgtgaccgtctccgggagctgcatgtgtcagaggttttcaccgtcatcaccgaaacgcgcgag
gcagctgcggtaaagctcatcagcgtggtcgtgaagcgattcacagatgtctgcctgttcatccgcgtcc
agctcgttgagtttctccagaagcgttaatgtctggcttctgataaagcgggccatgttaagggcggttt

Uppercase, underlined sequence represents CbAgo

B. pET28a-6Xhis-HRV3C-KmAgo

tggcgaatgggacgcgccctgtagcggcgcattaagcgcggcgggtgtggtggttacgcgcagcgtgacc
gctacacttgccagcgccctagcgcccgctcctttcgctttcttcccttcctttctcgccacgttcgccg
gctttccccgtcaagctctaaatcgggggctccctttagggttccgatttagtgctttacggcacctcga
ccccaaaaaacttgattagggtgatggttcacgtagtgggccatcgccctgatagacggtttttcgccct
ttgacgttggagtccacgttctttaatagtggactcttgttccaaactggaacaacactcaaccctatct
cggtctattcttttgatttataagggattttgccgatttcggcctattggttaaaaaatgagctgattta
acaaaaatttaacgcgaattttaacaaaatattaacgtttacaatttcaggtggcacttttcggggaaat
gtgcgcggaacccctatttgtttatttttctaaatacattcaaatatgtatccgctcatgaattaattct
tagaaaaactcatcgagcatcaaatgaaactgcaatttattcatatcaggattatcaataccatattttt
gaaaaagccgtttctgtaatgaaggagaaaactcaccgaggcagttccataggatggcaagatcctggta
tcggtctgcgattccgactcgtccaacatcaatacaacctattaatttcccctcgtcaaaaataaggtta
tcaagtgagaaatcaccatgagtgacgactgaatccggtgagaatggcaaaagtttatgcatttctttcc
agacttgttcaacaggccagccattacgctcgtcatcaaaatcactcgcatcaaccaaaccgttattcat
tcgtgattgcgcctgagcgagacgaaatacgcgatcgctgttaaaaggacaattacaaacaggaatcgaa
tgcaaccggcgcaggaacactgccagcgcatcaacaatattttcacctgaatcaggatattcttctaata
cctggaatgctgttttcccggggatcgcagtggtgagtaaccatgcatcatcaggagtacggataaaatg
cttgatggtcggaagaggcataaattccgtcagccagtttagtctgaccatctcatctgtaacatcattg
gcaacgctacctttgccatgtttcagaaacaactctggcgcatcgggcttcccatacaatcgatagattg
tcgcacctgattgcccgacattatcgcgagcccatttatacccatataaatcagcatccatgttggaatt
taatcgcggcctagagcaagacgtttcccgttgaatatggctcataacaccccttgtattactgtttatg
taagcagacagttttattgttcatgaccaaaatcccttaacgtgagttttcgttccactgagcgtcagac
cccgtagaaaagatcaaaggatcttcttgagatcctttttttctgcgcgtaatctgctgcttgcaaacaa
aaaaaccaccgctaccagcggtggtttgtttgccggatcaagagctaccaactctttttccgaaggtaac
tggcttcagcagagcgcagataccaaatactgtccttctagtgtagccgtagttaggccaccacttcaag
aactctgtagcaccgcctacatacctcgctctgctaatcctgttaccagtggctgctgccagtggcgata
agtcgtgtcttaccgggttggactcaagacgatagttaccggataaggcgcagcggtcgggctgaacggg
gggttcgtgcacacagcccagcttggagcgaacgacctacaccgaactgagatacctacagcgtgagcta
tgagaaagcgccacgcttcccgaagggagaaaggcggacaggtatccggtaagcggcagggtcggaacag
gagagcgcacgagggagcttccaggggg aaacgcctggtatctttatagtcctgtcgggtttcgccacct
ctgacttgagcgtcgattttttgtgatgctcgtcaggggggcggagcctatggaaaaacgccagcaacgcg

gccttttacggttcctggccttttgctggccttttgctcacatgttctttcctgcgttatcccctgatt
ctgtggataaccgtattaccgcctttgagtgagctgataccgctcgccgcagccgaacgaccgagcgcag
cgagtcagtgagcgaggaagcggaagagcgcctgatgcggtattttctccttacgcatctgtgcggtatt
tcacaccgcatatatggtgcactctcagtacaatctgctctgatgccgcatagttaagccagtatacact
ccgctatcgctacgtgactgggtcatggctgcgccccgacacccgccaacacccgctgacgcgccctgac
gggcttgtctgctcccggcatccgcttacagacaagctgtgaccgtctccgggagctgcatgtgtcagag
gttttcaccgtcatcaccgaaacgcgcgaggcagctgcggtaaagctcatcagcgtggtcgtgaagcgat
tcacagatgtctgcctgttcatccgcgtccagctcgttgagtttctccagaagcgttaatgtctggcttc
tgataaagcgggccatgttaagggcggttttttcctgtttggtcactgatgcctccgtgtaagggggatt
tctgttcatgggggtaatgataccgatgaaacgagagaggatgctcacgatacgggttactgatgatgaa
catgcccggttactggaacgttgtgagggtaaacaactggcggtatggatgcggcgggaccagagaaaaa
tcactcagggtcaatgccagcgcttcgttaatacagatgtaggtgttccacagggtagccagcagcatcc
tgcgatgcagatccggaacataatggtgcagggcgctgacttccgcgtttccagactttacgaaacacgg
aaaccgaagaccattcatgttgttgctcaggtcgcagacgttttgcagcagcagtcgcttcacgttcgct
cgcgtatcggtgattcattctgctaaccagtaaggcaaccccgccagcctagccgggtcctcaacgacag
gagcacgatcatgcgcacccgtggggccgccatgccggcgataatggcctgcttctcgccgaaacgtttg
gtggcgggaccagtgacgaaggcttgagcgagggcgtgcaagattccgaataccgcaagcgacaggccga
tcatcgtcgcgctccagcgaaagcggtcctcgccgaaaatgacccagagcgctgccggcacctgtcctac
gagttgcatgataaagaagacagtcataagtgcggcgacgatagtcatgccccgcgcccaccggaaggag
ctgactgggttgaaggctctcaagggcatcggtcgagatcccggtgcctaatgagtgagctaacttacat
taattgcgttgcgctcactgcccgctttccagtcgggaaacctgtcgtgccagctgcattaatgaatcgg
ccaacgcgcggggagaggcggtttgcgtattgggcgccagggtggtttttcttttcaccagtgagacggg
caacagctgattgcccttcaccgcctggccctgagagagttgcagcaagcggtccacgctggtttgcccc
agcaggcgaaaatcctgtttgatggtggttaacggcgggatataacatgagctgtcttcggtatcgtcgt
atcccactaccgagatatccgcaccaacgcgcagcccggactcggtaatggcgcgcattgcgcccagcgc
catctgatcgttggcaaccagcatcgcagtgggaacgatgccctcattcagcatttgcatggtttgttga
aaaccggacatggcactccagtcgccttcccgttccgctatcggctgaatttgattgcgagtgagatatt
tatgccagccagccagacgcagacgcgccgagacagaacttaatgggcccgctaacagcgcgatttgctg
gtgacccaatgcgaccagatgctccacgcccagtcgcgtaccgtcttcatgggagaaaataatactgttg
atgggtgtctggtcagagacatcaagaaataacgccggaacattagtgcaggcagcttccacagcaatgg
catcctggtcatccagcggatagttaatgatcagcccactgacgcgttgcgcgagaagattgtgcaccgc
cgctttacaggcttcgacgccgcttcgttctaccatcgacaccaccacgctggcacccagttgatcggcg
cgagatttaatcgccgcgacaatttgcgacggcgcgtgcagggccagactggaggtggcaacgccaatca
gcaacgactgtttgcccgccagttgttgtgccacgcggttgggaatgtaattcagctccgccatcgccgc
ttccactttttcccgcgttttcgcagaaacgtggctggcctggttcaccacgcgggaaacggtctgataa
gagacaccggcatactctgcgacatcgtataacgttactggtttcacattcaccaccctgaattgactct
cttccgggcgctatcatgccataccgcgaaaggttttgcgccattcgatggtgtccgggatctcgacgct
ctcccttatgcgactcctgcattaggaagcagcccagtagtaggttgaggccgttgagcaccgccgccgc
aaggaatggtgcatgcaaggagatggcgcccaacagtcccccggccacggggcctgccaccatacccacg
ccgaaacaagcgctcatgagcccgaagtggcgagcccgatcttccccatcggtgatgtcggcgatatagg
cgccagcaaccgcacctgtggcgccggtgatgccggccacgatgcgtccggcgtagaggatcgagatctc
gatcccgcgaaattaatacgactcactataggggaattgtgagcggataacaattcccctctagaaataa
ttttgtttaactttaagaaggagatataccATGCATCACCATCACCATCACACTAGTGGCTCTTTGGAGG
TTTTGTTCCAGGGTCCAGGTTCAGGCAGTGGATCCCGCATCAGCGAATTCATGGAAGCTTACATCACCGA
GATGGTGTCCAGAGAAAGAGCTAACGAGTTGGAGGTTTACGTCTACGTGTTCCCAAGAAAGCAGTCCGAC
AACAACTACGAGGGTGTCTACCACATTATGAGAGCTTGGCAAAGAGCCAACGACTTGCCATTGGCTTACA
ACCAGCACACCATCATGGCTTTCTCACCAGTTAGACACATGTGCGGTTACACCCCAATGGAAACTCAGAA
GAGACACATCAACATCGACTCCCCATTCGAGAGAGCTTTGTTGGAGAGACTGATCAAGAACTCCTTGATC
TTCACTGCCGAGAGACACTTGCATGCTAAGAGAGTTGGTCACGCCTTGAGATTGAACCAGGTTCAGCAAA
TCAGGCAGGTCATCATCTACGAGGCTATCGAGTTGTACGTCAACATCATCGAGAACAGGATCTCCATCGG
TTTCCACTTGACTCACCAATTCGAGTACGTCTACACCCTGCAGTCCATGATTGAGCAGGGTAAGACTATC
AGACCAGGTATGAGAGTTGTCCACTCCAACGGTAGACAGCACTACACTTACACCGTTGAGAACGTTGCTA

CCTACGGTGTTACTGACAGATGTCCTTTGTTGCAGACCTCCATCTACCAGTACTACGTTGAGAAGGGTGC
TCAGCACATCTTGAGAACTTTCACCAGATCCACCAGAGTCATCCACGTTAGGACCAAAGAGCAGAGATTG
TCCTACGCTGCTACCTTGTTGAAGCCATTGTGTACCTTCGAGACTATGCAGCCACAGGACGTTTTGAACG
TTTCCAAGTGCATCAAGTTGTCCGCCTCCAAGAGAATGAAGTGCACCTACAGATGGATTCAGCAGTTGAG
AGCCCAGTACAGACACTTGACTTTCGCCCCAAATCCATTCACTATCGCCCAGAACGGTTACAAGTTGGAC
CAGTTGTCTACCCCAAAGGTCCACTTCCATAGAGACTACGCTACTGTTGTCTCCGGTATGAAGACCGGTA
AGTTGTACAAAGGTGGTAACATCAAGATCTCCGTCCTGTTCGATGAGGACTTCTACTTGAAGCACCACAT
CACCAAGAAGGATATCTACCAATTCATTGCCGTCCTGCAGAAGATCGCTATTGCTCAGGGTGTTAACATG
ACCATCTCCACCTCCACTAAGTCCATCACTGGTAAGTTCACCGACGATTTCTTCCACCACTTCACCGAAG
AGGTTGAAGCCTTGCAACCTATCTTCGCTCAGACTACTGTTCTGGCCTTCATCACTTCTACCCACCTGTC
CAACAAGAAAACCAGGTCCTACCAATTGCTGAAGCAGTACTTTGGTGGTAAGTGGGACATTGCTTCCCAG
GTTATCACCGAAAAGACTATCGAGGCCTTCCAAAAGATCCTGCACAAGCACGGTCTGAAGAACTTTTACC
CAAACGACGAGCAGCACTGCTTGAGAGTTATTGACGTCTTGAAGAACGAGTCCTTCTACTACACCGTCAT
GAACATCCTGCTGGGTGTTTACGTTAAGTCCGGTATTCAGCCATGGATCTTGGCTAACACTACTCACTCC
GACTGCTTCATCGGTATTGACGTTTCTCACGAGAACGGTAACTCTGCTGCTGGTATGATGAACGTTATTG
GTTCCCAGGGTCACTTGATCCAACAGGCTCCATTGAACGGTATTTTGGCCGGTGAAAAGATCGACGACAC
CTTGTTGGCCAATCTGTTGAAGCAGATGATCAAGGCCTACCACACTCAGTTCCAGAGATTCCCAAAGCAC
ATCACTATCCACCGTGACGGTTTTTGGAGAGAACACACTGCTTTGGTCGAGAAGATCATGTCTCACTACG
AGATCACCTACGACATCGTCGAGATCATCAAAAAGCCAAACAGAAGGATGGCCTTCTTCAACTCCGTTGA
CAACACTTTCTCCACCAGACAGGGTACTGTTTACCAGAGAGGTAACGAGGCTTTCCTGTGTGCTACAAAC
CCACAGCAAAAGGTTGGTATGGCTCAGCCAATCAAGATTCACCAGGTTACCAAGACCTTGCCATTCTCTC
ACATTATCGAGGACGTGTACAACCTGTCCTTCTTGCACATTCACGCCATGAACAAGATGAGATTGCCAGC
CACTATTCACTACGCTGACTTGTCTGCTACTGCTTACCAACGTGGTCAGGTTATGCCTAGATCTGGTAAC
CAGACCAACCTGCCATTCGTTtaagtcgacctcgagcaccaccaccaccaccactgagatccggctgcta
acaaagcccgaaaggaagctgagttggctgctgccaccgctgagcaataactagcataaccccttggggc
ctctaaacgggtcttgaggggttttttgctgaaaggaggaactatatccggat

Uppercase, underlined sequence represents KmAgo

2) Target plasmids used in this study

A. pMRS plasmid used to clone all γPNA target regions between the EcoRI and BamHI restriction sites (underlined and highlighted) (5,418 bp)

tagttattaatagtaatcaattacggggtcattagttcatagcccatatatggagttccgcgttacataa
cttacggtaaatggcccgcctggctgaccgcccaacgacccccgcccattgacgtcaataatgacgtatg
ttcccatagtaacgccaatagggactttccattgacgtcaatgggtggagtatttacggtaaactgccca
cttggcagtacatcaagtgtatcatatgccaagtacgccccctattgacgtcaatgacggtaaatggccc
gcctggcattatgcccagtacatgaccttatgggactttcctacttggcagtacatctacgtattagtca
tcgctattaccatggtgatgcggttttggcagtacatcaatgggcgtggatagcggtttgactcacgggg
atttccaagtctccaccccattgacgtcaatgggagtttgttttggcaccaaaatcaacgggactttcca
aaatgtcgtaacaactccgccccattgacgcaaatgggcggtaggcgtgtacggtgggaggtctatataa
gcagagctggtttagtgaaccgtcagatccgcttgccaccatggcctcctccgaggacgtcatcaaggag
ttcatgcgcttcaaggtgcgcatggagggctccgtgaacggccacgagttcgagatcgagggcgagggcg
agggccgcccctacgagggcacccagaccgccaagctgaaggtgaccaagggcggccccctgcccttcgc
ctgggacatcctgtcccctcagttccagtacggctccaaggcctacgtgaagcaccccgccgacatcccc
gactacttgaagctgtccttccccgagggcttcaagtgggagcgcgtgatgaacttcgaggacggcggcg
tggtgaccgtgacccaggactcctccctgcaggacggcgagttcatctacaaggtgaagctgcgcggcac
caacttcccctccgacggccccgtaatgcagaagaagaccatgggctgggaggcctccaccgagcggatg
taccccgaggacggcgccctgaagggcgagatcaagatgaggctgaagctgaaggacggcggccactacg

acgccgaggtcaagaccacctacatggccaagaagcccgtgcagctgcccggcgcctacaagaccgacat
caagctggacatcacctcccacaacgaggactacaccatcgtggaacagtacgagcgcgccgagggccgc
cactccaccggcgccgaattcCCGCTAGTGCGATGGCCTCATGGAAGCTTGATATCCAGCCAGGACAATT
TACCgatCCATGAGGCCATCGCACTAGGGGggatccagtgagcaagggcgaggagctgttcaccggggtg
gtgcccatcctggtcgagctggacggcgacgtaaacggccacaagttcagcgtgtccggcgagggcgagg
gcgatgccacctacggcaagctgaccctgaagttcatctgcaccaccggcaagctgcccgtgccctggcc
TacActGgtTacTacATtAacAtaTggAgtCcaAtgTttTagccgctaccccgaccacatgaagcagcac
gacttcttcaagtccgccatgcccgaaggctacgtccaggagcgcaccatcttcttcaaggacgacggca
actacaagacccgcgccgaggtgaagttcgagggcgacaccctggtgaaccgcatcgagctgaagggcat
cgacttcaaggaggacggcaacatcctggggcacaagctggagtacaactacaacagccacaacgtctat
atcatggccgacaagcagaagaacggcatcaaggtgaacttcaagatccgccacaacatcgaggacggca
gcgtgcagctcgccgaccactaccagcagaacacccccatcggcgacggccccgtgctgctgcccgacaa
ccactacctgagcacccagtccgccctgagcaaagaccccaacgagaagcgcgatcacatggtcctgctg
gagttcgtgaccgccgccgggatcactctcggcatggacgagctgtacaagtaagcggccgcgactctag
atcataatcagccataccacatttgtagaggttttacttgctttaaaaaacctcccacacctccccctga
acctgaaacataaaatgaatgcaattgttgttgttaacttgtttattgcagcttataatggttacaaata
aagcaatagcatcacaaatttcacaaataaagcatttttttcactgcattctagttgtggtttgtccaaa
ctcatcaatgtatcttaaggcgtaaattgtaagcgttaatattttgttaaaattcgcgttaaatttttgt
taaatcagctcattttttaaccaataggccgaaatcggcaaaatcccttataaatcaaaagaatagaccg
agatagggttgagtgttgttccagtttggaacaagagtccactattaaagaacgtggactccaacgtcaa
agggcgaaaaaccgtctatcagggcgatggcccactacgtgaaccatcaccctaatcaagttttttgggg
tcgaggtgccgtaaagcactaaatcggaaccctaaagggagcccccgatttagagcttgacggggaaagc
cggcgaacgtggcgagaaaggaagggaagaaagcgaaaggagcgggcgctagggcgctggcaagtgtagc
ggtcacgctgcgcgtaaccaccacacccgccgcgcttaatgcgccgctacagggcgcgtcaggtggcact
tttcggggaaatgtgcgcggaacccctatttgtttatttttctaaatacattcaaatatgtatccgctca
tgagacaataaccctgataaatgcttcaataatattgaaaaaggaagagtcctgaggcggaaagaaccag
ctgtggaatgtgtgtcagttagggtgtggaaagtccccaggctccccagcaggcagaagtatgcaaagca
tgcatctcaattagtcagcaaccaggtgtggaaagtccccaggctccccagcaggcagaagtatgcaaag
catgcatctcaattagtcagcaaccatagtcccgcccctaactccgcccatcccgcccctaactccgccc
agttccgcccattctccgccccatggctgactaattttttttatttatgcagaggccgaggccgcctcgg
cctctgagctattccagaagtagtgaggaggcttttttggaggcctaggcttttgcaaagatcgatcaag
agacaggatgaggatcgtttcgcatgattgaacaagatggattgcacgcaggttctccggccgcttgggt
ggagaggctattcggctatgactgggcacaacagacaatcggctgctctgatgccgccgtgttccggctg
tcagcgcaggggcgcccggttctttttgtcaagaccgacctgtccggtgccctgaatgaactgcaagacg
aggcagcgcggctatcgtggctggccacgacgggcgttccttgcgcagctgtgctcgacgttgtcactga
agcgggaagggactggctgctattgggcgaagtgccggggcaggatctcctgtcatctcaccttgctcct
gccgagaaagtatccatcatggctgatgcaatgcggcggctgcatacgcttgatccggctacctgcccat
tcgaccaccaagcgaaacatcgcatcgagcgagcacgtactcggatggaagccggtcttgtcgatcagga
tgatctggacgaagagcatcaggggctcgcgccagccgaactgttcgccaggctcaaggcgagcatgccc
gacggcgaggatctcgtcgtgacccatggcgatgcctgcttgccgaatatcatggtggaaaatggccgct
tttctggattcatcgactgtggccggctgggtgtggcggaccgctatcaggacatagcgttggctacccg
tgatattgctgaagagcttggcggcgaatgggctgaccgcttcctcgtgctttacggtatcgccgctccc
gattcgcagcgcatcgccttctatcgccttcttgacgagttcttctgagcgggactctggggttcgaaat
gaccgaccaagcgacgcccaacctgccatcacgagatttcgattccaccgccgccttctatgaaaggttg
ggcttcggaatcgttttccgggacgccggctggatgatcctccagcgcggggatctcatgctggagttct
tcgcccaccctagggggaggctaactgaaacacggaaggagacaataccggaaggaacccgcgctatgac
ggcaataaaaagacagaataaaacgcacggtgttgggtcgtttgttcataaacgcggggttcggtcccag
ggctggcactctgtcgataccccaccgagaccccattggggccaatacgcccgcgtttcttccttttccc
caccccaccccccaagttcgggtgaaggcccagggctcgcagccaacgtcggggcggcaggccctgccat
agcctcaggttactcatatatactttagattgatttaaaacttcatttttaatttaaaaggatctaggtg
aagatcctttttgataatctcatgaccaaaatcccttaacgtgagttttcgttccactgagcgtcagacc
ccgtagaaaagatcaaaggatcttcttgagatcctttttttctgcgcgtaatctgctgcttgcaaacaaa

aaaaccaccgctaccagcggtggtttgtttgccggatcaagagctaccaactctttttccgaaggtaact
ggcttcagcagagcgcagataccaaatactgtccttctagtgtagccgtagttaggccaccacttcaaga
actctgtagcaccgcctacatacctcgctctgctaatcctgttaccagtggctgctgccagtggcgataa
gtcgtgtcttaccgggttggactcaagacgatagttaccggataaggcgcagcggtcgggctgaacgggg
ggttcgtgcacacagcccagcttggagcgaacgacctacaccgaactgagatacctacagcgtgagctat
gagaaagcgccacgcttcccgaagggagaaaggcggacaggtatccggtaagcggcagggtcggaacagg
agagcgcacgagggagcttccagggggaaacgcctggtatctttatagtcctgtcgggtttcgccacctc
tgacttgagcgtcgatttttgtgatgctcgtcaggggggcggagcctatggaaaaacgccagcaacgcgg
cctttttacggttcctggccttttgctggccttttgctcacatgttctttcctgcgttatcccctgattc
tgtggataaccgtattaccgccatgcat

B. pUC19 plasmid used to clone all γPNA target regions between EcoRI and BamHI restriction sites (underlined and highlighted) (2,686 bp)

TCGCGCGTTTCGGTGATGACGGTGAAAACCTCTGACACATGCAGCTCCCGGAGACGGTCACAGCTTGTCT
GTAAGCGGATGCCGGGAGCAGACAAGCCCGTCAGGGCGCGTCAGCGGGTGTTGGCGGGTGTCGGGGCTGG
CTTAACTATGCGGCATCAGAGCAGATTGTACTGAGAGTGCACCATATGCGGTGTGAAATACCGCACAGAT
GCGTAAGGAGAAAATACCGCATCAGGCGCCATTCGCCATTCAGGCTGCGCAACTGTTGGGAAGGGCGATC
GGTGCGGGCCTCTTCGCTATTACGCCAGCTGGCGAAAGGGGGATGTGCTGCAAGGCGATTAAGTTGGGTA
ACGCCAGGGTTTTCCCAGTCACGACGTTGTAAAACGACGGCCAGTGAATTCGAGCTCGGTACCCGGGGAT
CCTCTAGAGTCGACCTGCAGGCATGCAAGCTTGGCGTAATCATGGTCATAGCTGTTTCCTGTGTGAAATT
GTTATCCGCTCACAATTCCACACAACATACGAGCCGGAAGCATAAAGTGTAAAGCCTGGGGTGCCTAATG
AGTGAGCTAACTCACATTAATTGCGTTGCGCTCACTGCCCGCTTTCCAGTCGGGAAACCTGTCGTGCCAG
CTGCATTAATGAATCGGCCAACGCGCGGGGAGAGGCGGTTTGCGTATTGGGCGCTCTTCCGCTTCCTCGC
TCACTGACTCGCTGCGCTCGGTCGTTCGGCTGCGGCGAGCGGTATCAGCTCACTCAAAGGCGGTAATACG
GTTATCCACAGAATCAGGGGATAACGCAGGAAAGAACATGTGAGCAAAAGGCCAGCAAAAGGCCAGGAAC
CGTAAAAAGGCCGCGTTGCTGGCGTTTTTCCATAGGCTCCGCCCCCCTGACGAGCATCACAAAAATCGAC
GCTCAAGTCAGAGGTGGCGAAACCCGACAGGACTATAAAGATACCAGGCGTTTCCCCCTGGAAGCTCCCT
CGTGCGCTCTCCTGTTCCGACCCTGCCGCTTACCGGATACCTGTCCGCCTTTCTCCCTTCGGGAAGCGTG
GCGCTTTCTCATAGCTCACGCTGTAGGTATCTCAGTTCGGTGTAGGTCGTTCGCTCCAAGCTGGGCTGTG
TGCACGAACCCCCCGTTCAGCCCGACCGCTGCGCCTTATCCGGTAACTATCGTCTTGAGTCCAACCCGGT
AAGACACGACTTATCGCCACTGGCAGCAGCCACTGGTAACAGGATTAGCAGAGCGAGGTATGTAGGCGGT
GCTACAGAGTTCTTGAAGTGGTGGCCTAACTACGGCTACACTAGAAGAACAGTATTTGGTATCTGCGCTC
TGCTGAAGCCAGTTACCTTCGGAAAAAGAGTTGGTAGCTCTTGATCCGGCAAACAAACCACCGCTGGTAG
CGGTGGTTTTTTTGTTTGCAAGCAGCAGATTACGCGCAGAAAAAAAGGATCTCAAGAAGATCCTTTGATC
TTTTCTACGGGGTCTGACGCTCAGTGGAACGAAAACTCACGTTAAGGGATTTTGGTCATGAGATTATCAA
AAAGGATCTTCACCTAGATCCTTTTAAATTAAAAATGAAGTTTTAAATCAATCTAAAGTATATATGAGTA
AACTTGGTCTGACAGTTACCAATGCTTAATCAGTGAGGCACCTATCTCAGCGATCTGTCTATTTCGTTCA
TCCATAGTTGCCTGACTCCCCGTCGTGTAGATAACTACGATACGGGAGGGCTTACCATCTGGCCCCAGTG
CTGCAATGATACCGCGAGACCCACGCTCACCGGCTCCAGATTTATCAGCAATAAACCAGCCAGCCGGAAG
GGCCGAGCGCAGAAGTGGTCCTGCAACTTTATCCGCCTCCATCCAGTCTATTAATTGTTGCCGGGAAGCT
AGAGTAAGTAGTTCGCCAGTTAATAGTTTGCGCAACGTTGTTGCCATTGCTACAGGCATCGTGGTGTCAC
GCTCGTCGTTTGGTATGGCTTCATTCAGCTCCGGTTCCCAACGATCAAGGCGAGTTACATGATCCCCCAT
GTTGTGCAAAAAAGCGGTTAGCTCCTTCGGTCCTCCGATCGTTGTCAGAAGTAAGTTGGCCGCAGTGTTA
TCACTCATGGTTATGGCAGCACTGCATAATTCTCTTACTGTCATGCCATCCGTAAGATGCTTTTCTGTGA
CTGGTGAGTACTCAACCAAGTCATTCTGAGAATAGTGTATGCGGCGACCGAGTTGCTCTTGCCCGGCGTC
AATACGGGATAATACCGCGCCACATAGCAGAACTTTAAAAGTGCTCATCATTGGAAAACGTTCTTCGGGG
CGAAAACTCTCAAGGATCTTACCGCTGTTGAGATCCAGTTCGATGTAACCCACTCGTGCACCCAACTGAT
CTTCAGCATCTTTTACTTTCACCAGCGTTTCTGGGTGAGCAAAAACAGGAAGGCAAAATGCCGCAAAAAA
GGGAATAAGGGCGACACGGAAATGTTGAATACTCATACTCTTCCTTTTTCAATATTATTGAAGCATTTAT

CAGGGTTATTGTCTCATGAGCGGATACATATTTGAATGTATTTAGAAAAATAAACAAATAGGGGTTCCGC
GCACATTTCCCCGAAAAGTGCCACCTGACGTCTAAGAAACCATTATTATCATGACATTAACCTATAAAAA
TAGGCGTATCACGAGGCCCTTTCGTC

C. pMRS multiplexing plasmid

TAGTTATTAATAGTAATCAATTACGGGGTCATTAGTTCATAGCCCATATATGGAGTTCCGCGTTACATAA
CTTACGGTAAATGGCCCGCCTGGCTGACCGCCCAACGACCCCCGCCCATTGACGTCAATAATGACGTATG
TTCCCATAGTAACGCCAATAGGGACTTTCCATTGACGTCAATGGGTGGAGTATTTACGGTAAACTGCCCA
CTTGGCAGTACATCAAGTGTATCATATGCCAAGTACGCCCCCTATTGACGTCAATGACGGTAAATGGCCC
GCCTGGCATTATGCCCAGTACATGACCTTATGGGACTTTCCTACTTGGCAGTACATCTACGTATTAGTCA
TCGCTATTACCATGGTGATGCGGTTTTGGCAGTACATCAATGGGCGTGGATAGCGGTTTGACTCACGGGG
ATTTCCAAGTCTCCACCCCATTGACGTCAATGGGAGTTTGTTTTGGCACCAAAATCAACGGGACTTTCCA
AAATGTCGTAACAACTCCGCCCCATTGACGCAAATGGGCGGTAGGCGTGTACGGTGGGAGGTCTATATAA
GCAGAGCTGGTTTAGTGAACCGTCAGATCCGCTTGCCACCATGGCCTCCTCCGAGGACGTCATCAAGGAG
TTCATGCGCTTCAAGGTGCGCATGGAGGGCTCCGTGAACGGCCACGAGTTCGAGATCGAGGGCGAGGGCG
AGGGCCGCCCCTACGAGGGCACCCAGACCGCCAAGCTGAAGGTGACCAAGGGCGGCCCCCTGCCCTTCGC
CTGGGACATCCTGTCCCCTCAGTTCCAGTACGGCTCCAAGGCCTACGTGAAGCACCCCGCCGACATCCCC
GACTACTTGAAGCTGTCCTTCCCCGAGGGCTTCAAGTGGGAGCGCGTGATGAACTTCGAGGACGGCGGCG
TGGTGACCGTGACCCAGGACTCCTCCCTGCAGGACGGCGAGTTCATCTACAAGGTGAAGCTGCGCGGCAC
CAACTTCCCCTCCGACGGCCCCGTAATGCAGAAGAAGACCATGGGCTGGGAGGCCTCCACCGAGCGGATG
TACCCCGAGGACGGCGCCCTGAAGGGCGAGATCAAGATGAGGCTGAAGCTGAAGGACGGCGGCCACTACG
ACGCCGAGGTCAAGACCACCTACATGGCCAAGAAGCCCGTGCAGCTGCCCGGCGCCTACAAGACCGACAT
CAAGCTGGACATCACCTCCCACAACGAGGACTACACCATCGTGGAACAGTACGAGCGCGCCGAGGGCCGC
CACTCCACCGGCGCCGAATTGCCCACCCTCGTGACCACCCAAGGGAGGTTCTCAATGCGGAGAGCTCGAT
CCAGTGAGCAAGGGCGAGGAGCTGTTCACCGGGGTGGTGCCCATCCTGGTCGAGCTGGACGGCGACGTAA
ACGGCCACAAGTTCAGCGTGTCCGGCGAGGGCGAGGGCGATGCCACCTACGGCAAGCTGACCCTGAAGTT
CATCTGCACCACCGGCAAGCTGCCCGTGCCCTGGCCTACACTGGTTACTACATTAACATATGGAGTCCAA
TGTTTTAGCCGCTACCCCGACCACATGAAGCAGCACGACTTCTTCAAGTCCGCCATGCCCGAAGGCTACG
TCCAGGAGCGCACCATCTTCTTCAAGGACGACGGCAACTACAAGACCCGCGCCGAGGTGAAGTTCGAGGG
CGACACCCTGGTGAACCGCATCGAGCTGAAGGGCATCGACTTCAAGGAGGACGGCAACATCCTGGGGCAC
AAGCTGGAGTACAACTACAACAGCCACAACGTCTATATCATGGCCGACAAGCAGAAGAACGGCATCAAGG
TGAACTTCAAGATCCGCCACAACATCGAGGACGGCAGCGTGCAGCTCGCCGACCACTACCAGCAGAACAC
CCCCATCGGCGACGGCCCCGTGCTGCTGCCCGACAACCACTACCTGAGCACCCAGTCCGCCCTGAGCAAA
GACCCCAACGAGAAGCGCGATCACATGGTCCTGCTGGAGTTCGTGACCGCCGCCGGGATCACTCTCGGCA
TGGACGAGCTGTACAAGTAAGCGGCCTACAAACGGCAGAAGCTGGAGGAGGAAGGGCCTGAGTCCGAGCA
GAAGAAGAAGGGCTCCCATCACATCAACCGGTGGCGCATTGCCACGAAGCAGGCCAAATTGTTGTTGTTA
ACTTGTTTATTGCAGCTTATAATGGTTACAAATAAAGCAATAGCATCACAAATTTCACAAATAAAGCATT
TTTTTCACTGCATTCTAGTTGTGGTTTGTCCAAACTCATCAATGTATCTTAAGGCGTAAATTGTAAGCGT
TAATATTTTGTTAAAATTCGCGTTAAATTTTTGTTAAATCAGCTCATTTTTTAACCAATAGGCCGAAATC
GGCAAAATCCCTTATAAATCAAAAGAATAGACCGAGATAGGGTTGAGTGTTGTTCCAGTTTGGAACAAGA
GTCCACTATTAAAGAACGTGGACTCCAACGTCAAAGGGCGAAAAACCGTCTATCAGGGCGATGGCCCACT
ACGTGAACCATCACCCTAATCAAGTTTTTTGGGGTCGAGGTGCCGTAAAGCACTAAATCGGAACCCTAAA
GGGAGCCCCCGATTTAGAGCTTGACGGGGAAAGCCGGCGAACGTGGCGAGAAAGGAAGGGAAGAAAGCGA
AAGGAGCGGGCGCTAGGGCGCTGGCAAGTGTAGCGGTCACGCTGCGCGTAACCACCACACCCGCCGCGCT
TAATGCGCCGCTACAGGGCGCGTCAGGTGGCACTTTTCGGGGAAATGTGCGCGGAACCCCTATTTGTTTA
TTTTTCTAAATACATTCAAATATGTATCCGCTCATGAGACAATAACCCTGATAAATGCTTCAATAATATT

GAAAAAGGAAGAGTCCTGAGGCGGAAAGAACCAGCTGTGGAATGTGTGTCAGTTAGGGTGTGGAAAGTCC
CCAGGCTCCCCAGCAGGCAGAAGTATGCAAAGCATGCATCTCAATTAGTCAGCAACCAGGTGTGGAAAGT
CCCCAGGCTCCCCAGCAGGCAGAAGTATGCAAAGCATGCATCTCAATTAGTCAGCAACCATAGTCCCGCC
CCTAACTCCGCCCATCCCGCCCCTAACTCCGCCCAGTTCCGCCCATTCTCCGCCCCATGGCTGACTAATT
TTTTTTATTTATGCAGAGGCCGAGGCCGCCTCGGCCTCTGAGCTATTCCAGAAGTAGTGAGGAGGCTTTT
TTGGAGGCCTAGGCTTTTGCAAAGATCGATCAAGAGACAGGATGAGGATCGTTTCGCATGATTGAACAAG
ATGGATTGCACGCAGGTTCTCCGGCCGCTTGGGTGGAGAGGCTATTCGGCTATGACTGGGCACAACAGAC
AATCGGCTGCTCTGATGCCGCCGTGTTCCGGCTGTCAGCGCAGGGGCGCCCGGTTCTTTTTGTCAAGACC
GACCTGTCCGGTGCCCTGAATGAACTGCAAGACGAGGCAGCGCGGCTATCGTGGCTGGCCACGACGGGCG
TTCCTTGCGCAGCTGTGCTCGACGTTGTCACTGAAGCGGGAAGGGACTGGCTGCTATTGGGCGAAGTGCC
GGGGCAGGATCTCCTGTCATCTCACCTTGCTCCTGCCGAGAAAGTATCCATCATGGCTGATGCAATGCGG
CGGCTGCATACGCTTGATCCGGCTACCTGCCCATTCGACCACCAAGCGAAACATCGCATCGAGCGAGCAC
GTACTCGGATGGAAGCCGGTCTTGTCGATCAGGATGATCTGGACGAAGAGCATCAGGGGCTCGCGCCAGC
CGAACTGTTCGCCAGGCTCAAGGCGAGCATGCCCGACGGCGAGGATCTCGTCGTGACCCATGGCGATGCC
TGCTTGCCGAATATCATGGTGGAAAATGGCCGCTTTTCTGGATTCATCGACTGTGGCCGGCTGGGTGTGG
CGGACCGCTATCAGGACATAGCGTTGGCTACCCGTGATATTGCTGAAGAGCTTGGCGGCGAATGGGCTGA
CCGCTTCCTCGTGCTTTACGGTATCGCCGCTCCCGATTCGCAGCGCATCGCCTTCTATCGCCTTCTTGAC
GAGTTCTTCTGAGCGGGACTCTGGGGTTCGAAATGACCGACCAAGCGACGCCCAACCTGCCATCACGAGA
TTTCGATTCCACCGCCGCCTTCTATGAAAGGTTGGGCTTCGGAATCGTTTTCCGGGACGCCGGCTGGATG
ATCCTCCAGCGCGGGGATCTCATGCTGGAGTTCTTCGCCCACCCTAGGGGGAGGCTAACTGAAACACGGA
AGGAGACAATACCGGAAGGAACCCGCGCTATGACGGCAATAAAAAGACAGAATAAAACGCACGGTGTTGG
GTCGTTTGTTCATAAACGCGGGGTTCGGTCCCAGGGCTGGCACTCTGTCGATACCCCACCGAGACCCCAT
TGGGGCCAATACGCCCGCGTTTCTTCCTTTTCCCCACCCCACCCCCCAAGTTCGGGTGAAGGCCCAGGGC
TCGCAGCCAACGTCGGGGCGGCAGGCCCTGCCATAGCCTCAGGTTACTCATATATACTTTAGATTGATTT
AAAACTTCATTTTTAATTTAAAAGGATCTAGGTGAAGATCCTTTTTGATAATCTCATGACCAAAATCCCT
TAACGTGAGTTTTCGTTCCACTGAGCGTCAGACCCCGTAGAAAAGATCAAAGGATCTTCTTGAGATCCTT
TTTTTCTGCGCGTAATCTGCTGCTTGCAAACAAAAAAACCACCGCTACCAGCGGTGGTTTGTTTGCCGGA
TCAAGAGCTACCAACTCTTTTTCCGAAGGTAACTGGCTTCAGCAGAGCGCAGATACCAAATACTGTCCTT
CTAGTGTAGCCGTAGTTAGGCCACCACTTCAAGAACTCTGTAGCACCGCCTACATACCTCGCTCTGCTAA
TCCTGTTACCAGTGGCTGCTGCCAGTGGCGATAAGTCGTGTCTTACCGGGTTGGACTCAAGACGATAGTT
ACCGGATAAGGCGCAGCGGTCGGGCTGAACGGGGGGTTCGTGCACACAGCCCAGCTTGGAGCGAACGACC
TACACCGAACTGAGATACCTACAGCGTGAGCTATGAGAAAGCGCCACGCTTCCCGAAGGGAGAAAGGCGG
ACAGGTATCCGGTAAGCGGCAGGGTCGGAACAGGAGAGCGCACGAGGGAGCTTCCAGGGGGAAACGCCTG
GTATCTTTATAGTCCTGTCGGGTTTCGCCACCTCTGACTTGAGCGTCGATTTTTGTGATGCTCGTCAGGG
GGGCGGAGCCTATGGAAAAACGCCAGCAACGCGGCCTTTTTACGGTTCCTGGCCTTTTGCTGGCCTTTTG
CTCACATGTTCTTTCCTGCGTTATCCCCTGATTCTGTGGATAACCGTATTACCGCCATGCAT

Supplementary tables

Supplementary Table S1. Sequences of proteins used in this study

Protein name	Sequence

CbAgo	MNNLTFEAFEGIGQLNELNFYKYRLIGKGQIDNVHQAIWSVKYKLQANNFFKPVFVKGEIL<br>YSLDELKVIPEFENVEVILDGNIILSISENTDIYKDVIVFYINNALKNIKDITNYRKYITK<br>NTDEIICKSILTTNLKYQYMKSEKGFKLQRKFKISPVVFRNGKVILYLNCSSDFSTDKSIY<br>EMLNNGLDVVGLQVKNRWTNSNGNIFIEEVLDKSISEPGTSGKLGQSLIDYYINGNQKYRV<br>EKFTDEDKKAKVIKAKIKNKTYNYIPQALTPVITREYLSHTDKKFSKQIENVIKMDMNYRY<br>QTLKSFVEDIGVIKELNNLHFKNQYYTNFDFMGFESGILEEPVLMGANGKIKDKKQIFING<br>FFKNPKENVKFGVLYPEGCMENAQSIARSILDFATAGKYNKQENKYISKNLMNIGFKPSEC<br>IFESYKLGDITEYKATARKLKEHEKVGFVIAVIPDMNESEVENPYNPFKKVWAKLNIPSQM<br>ITLKTTEKFKNIVDKSGLYYLHNIALNILGKIGGIPWIIKDMPGNIDCFIGLDVGTREKGI<br>HFPACSVLFDKYGKLINYYKPTIPQSGEKIAETILQEIFDNVLISYKEENGEYPKNIVIHR<br>DGFSRENIDWYKEYFDKKGIKFNIIEVKKNIPVKIAKVVGSNICNPIKGSYVLKNDKAFIV<br>TTDIKDGVASPNPLKIEKTYGDVEMKSILEQIYSLSQIHVGSTKSLRLPITTGYADKICKA<br>IEYIPQGVVDNRLFFL
KmAgo	MEAYITEMVSRERANELEVYVYVFPRKQSDNNYEGVYHIMRAWQRANDLPLAYNQHTIMAF<br>SPVRHMCGYTPMETQKRHINIDSPFERALLERLIKNSLIFTAERHLHAKRVGHALRLNQVQ<br>QIRQVIIYEAIELYVNIIENRISIGFHLTHQFEYVYTLQSMIEQGKTIRPGMRVVHSNGRQ<br>HYTYTVENVATYGVTDRCPLLQTSIYQYYVEKGAQHILRTFTRSTRVIHVRTKEQRLSYAA<br>TLLKPLCTFETMQPQDVLNVSKCIKLSASKRMKCTYRWIQQLRAQYRHLTFAPNPFTIAQN<br>GYKLDQLSTPKVHFHRDYATVVSGMKTGKLYKGGNIKISVLFDEDFYLKHHITKKDIYQFI<br>AVLQKIAIAQGVNMTISTSTKSITGKFTDDFFHHFTEEVEALQPIFAQTTVLAFITSTHLS<br>NKKTRSYQLLKQYFGGKWDIASQVITEKTIEAFQKILHKHGLKNFYPNDEQHCLRVIDVLK<br>NESFYYTVMNILLGVYVKSGIQPWILANTTHSDCFIGIDVSHENGNSAAGMMNVIGSQGHL<br>IQQAPLNGILAGEKIDDTLLANLLKQMIKAYHTQFQRFPKHITIHRDGFWREHTALVEKIM<br>SHYEITYDIVEIIKKPNRRMAFFNSVDNTFSTRQGTVYQRGNEAFLCATNPQQKVGMAQPI<br>KIHQVTKTLPFSHIIEDVYNLSFLHIHAMNKMRLPATIHYADLSATAYQRGQVMPRSGNQT<br>NLPFV

Supplementary Table S2. PNAs (peptide nucleic acids) used in this study

Name	Sequence	PNA modifications
γPNA1	H-KKK-GCCCACCCTCGTGACCACCC-KKK-propargylglycine-NH_2	Gamma-alanine at all bases
γPNA2	H-KKK-AGCACTGCACGCCGTAGGTC-KKK-propargylglycine-NH_2	Gamma-alanine at all bases
γPNA3	H-KKK-TCCGCATTGAGAACCTCCCT-KKK-propargylglycine-NH_2	Gamma-alanine at all bases
γPNA4	H-KKK-TATATCCAGATTTTGTGACT-KKK-propargylglycine-NH_2	Gamma-alanine at all bases
γPNA5	H-KKK-TCTTCTTCTGCTCGGACTCA-KKK-propargylglycine-NH_2	Gamma-alanine at all bases
γPNA6	H-KKK-GGCTCCCATCACATCAACC-KKK-propargylglycine-NH_2	Gamma-alanine at all bases
γtcPNA1	Cy5-KKK-JTJJJTJJTJTJ-OOO-CT*CT*CC*TC*CC*TC*TT*CG*CC*-KKK-NH_2	X* indicates bases with gamma-alanine
γtcPNA2	Cy5-KKK-JTJJTTJTTJJ-OOO-CC*TT*CT*TC*CT*CG*CG*TC*CT*-KKK-NH_2	X* indicates bases with gamma-alanine
γPNA1-16nt	H-KKK-GCCCACCCTCGTGACC-KKK-propargylglycine-NH_2	Gamma-alanine at all bases
γPNA1-14nt	H-KKK-GCCCACCCTCGTGA-KKK-propargylglycine-NH_2	Gamma-alanine at all bases
γPNA1-10nt	H-KKK-GCCCACCCTC-KKK-propargylglycine-NH_2	Gamma-alanine at all bases
γPNA3-16nt	H-KKK-TCCGCATTGAGAACCT-KKK-propargylglycine-NH_2	Gamma-alanine at all bases
γPNA3-14nt	H-KKK-TCCGCATTGAGAAC-KKK-propargylglycine-NH_2	Gamma-alanine at all bases
γPNA3-10nt	H-KKK-TCCGCATTGA-KKK-propargylglycine-NH_2	Gamma-alanine at all bases

H, free amine at the N terminus.
NH_2, amide at the C terminus.
J, pseudoisocytosine.
K, lysine.
O, 8-Amino-3,6-dioxaoctanoic acid linkers.

Supplementary Table S3. Oligos used for cloning target regions into pMRS or pUC19

Name of the oligo	Sequence (5′ to 3′)	Figure(s) and supplementary figure(s)
γPNA1_γPNA3 target_1nt_ top	AATTGCCCACCCTCGTGACCACCCAAGGGAGGTTCTCAATGCGGAGAGCTC	1E, 1F, 2A, 2B, 3A, 3B, 4D, 5A, 5B, 7C, S1B, S2B, S2D, S5B, S6B, S8B, S9B, and S10B
γPNA1_γPNA3 target_1nt_ bottom	GATCGAGCTCTCCGCATTGAGAACCTCCCTTGGGTGGTCACGAGGGTGGGC	
γPNA5 top_in vitro target	AATTTACAAACGGCAGAAGCTGGAGGAGGAAGGGCCTGAGTCCGAGCAGAAGAAGAAGGGCTCCCATCACATCAACCGGTGGCGCATTGCCACGAAGCAGGCCA	3B, 6A-C, 6E
γPNA5 bottom_in vitro target	GATCTGGCCTGCTTCGTGGCAATGCGCCACCGGTTGATGTGATGGGAGCCCTTCTTCTTCTGCTCGGACTCAGGCCCTTCCTCCTCCAGCTTCTGCCGTTTGTA	
γPNA1_γPNA3 target_3-nt_ top	AATTGCCCACCCTCGTGACCACCCACAAGGGAGGTTCTCAATGCGGAGAGCTC	4D
γPNA1_γPNA3 target_3-nt_ bottom	GATCGAGCTCTCCGCATTGAGAACCTCCCTTGTGGGTGGTCACGAGGGTGGGC	
γPNA1_γPNA3 target_5-nt_ top	AATTGCCCACCCTCGTGACCACCCGAGCTCAGGGAGGTTCTCAATGCGGA	4D
γPNA1_γPNA3 target_5-nt_ bottom	GATCTCCGCATTGAGAACCTCCCTGAGCTCGGGTGGTCACGAGGGTGGGC	
PNA1/3 target-6 nt-top	GATCCGCCCACCCTCGTGACCACCCGAGCTCAGGGAGGTTCTCAATGCGGAG	4D
PNA1/3 target-6 nt-bottom	AATTCTCCGCATTGAGAACCTCCCTGAGCTCGGGTGGTCACGAGGGTGGGCG	
PNA1/3 target-10 nt-top	GATCCGCCCACCCTCGTGACCACCCTGGAGCTCTGAGGGAGGTTCTCAATGCGGAG	4D and S7B
PNA1/3 target-10 nt-bottom	AATTCTCCGCATTGAGAACCTCCCTCAGAGCTCCAGGGTGGTCACGAGGGTGGGCG	
PNA1/3 target-15 nt-top	GATCCGCCCACCCTCGTGACCACCCTGTGCGAGCTCACTGAGGGAGGTTCTCAATGCGGAG	4D
PNA1/3 target-15 nt-bottom	AATTCTCCGCATTGAGAACCTCCCTCAGTGAGCTCGCACAGGGTGGTCACGAGGGTGGGCG	
PNA1/3 target-20 nt-top	GATCCGCCCACCCTCGTGACCACCCTGTGCTCGAGCTCAGAACTGAGGGAGGTTCTCAATGCGGAG	4D
PNA1/3 target-20 nt-bottom	AATTCTCCGCATTGAGAACCTCCCTCAGTTCTGAGCTCGAGCACAGGGTGGTCACGAGGGTGGGCG	
PNA1/3 target-30 nt-top	GATCCGCCCACCCTCGTGACCACCCTGTGCTCAGTCAGAGCTCTGATGAGAACTGAGGGAGGTTCTCAATGCGGAG	4D
PNA1/3 target-30 nt-bottom	AATTCTCCGCATTGAGAACCTCCCTCAGTTCTCATCAGAGCTCTGACTGAGCACAGGGTGGTCACGAGGGTGGGCG	
γPNA1/3-1-nt spacer inward TOP	GATCGAGCTCGGGTGGTCACGAGGGTGGGCTTCCGCATTGAGAACCTCCCT	4E

γPNA1/3-1-nt spacer inward Bottom	AATTAGGGAGGTTCTCAATGCGGAAGCCCACCC TCGTGACCACCCGAGCTC	
γPNA1/3-3-nt spacer inward TOP	GATCGAGCTCGGGTGGTCACGAGGGTGGGCTGT TCCGCATTGAGAACCTCCCT	4E
γPNA1/3-3-nt spacer inward Bottom	AATTAGGGAGGTTCTCAATGCGGAACAGCCCAC CCTCGTGACCACCCGAGCTC	
γPNA1/3-5-nt spacer inward TOP	GATCGAGCTCGGGTGGTCACGAGGGTGGGCGCT GTTCCGCATTGAGAACCTCCCT	4E
γPNA1/3-5-nt spacer inward Bottom	AATTAGGGAGGTTCTCAATGCGGAACAGCGCCC ACCCTCGTGACCACCCGAGCTC	
γPNA1/3-6-nt spacer inward TOP	AATTCGGGTGGTCACGAGGGTGGGCGAGCTCTC CGCATTGAGAACCTCCCTG	4E
γPNA1/3-6-nt spacer inward Bottom	GATCCAGGGAGGTTCTCAATGCGGAGAGCTCGC CCACCCTCGTGACCACCCG	
γPNA1/3-10-nt spacer inward TOP	AATTCGGGTGGTCACGAGGGTGGGCCAGAGCTC CATCCGCATTGAGAACCTCCCTG	4E
γPNA1/3-10-nt spacer inward Bottom	GATCCAGGGAGGTTCTCAATGCGGATGGAGCTC TGGCCCACCCTCGTGACCACCCG	
γPNA1/3-15-nt spacer inward TOP	AATTCGGGTGGTCACGAGGGTGGGCCAGTGAGC TCGCACATCCGCATTGAGAACCTCCCTG	4E
γPNA1/3-15-nt spacer inward Bottom	GATCCAGGGAGGTTCTCAATGCGGATGTGCGAG CTCACTGGCCCACCCTCGTGACCACCCG	
γPNA1/3-20-nt spacer inward TOP	AATTCGGGTGGTCACGAGGGTGGGCCAGTTCTG AGCTCGAGCACATCCGCATTGAGAACCTCCCTG	4E
γPNA1/3-20-nt spacer inward Bottom	GATCCAGGGAGGTTCTCAATGCGGATGTGCTCG AGCTCAGAACTGGCCCACCCTCGTGACCACCCG	
γPNA1/3-30-nt spacer inward TOP	AATTCGGGTGGTCACGAGGGTGGGCCAGTTCTC ATCAGAGCTCTGACTGAGCACATCCGCATTGAG AACCTCCCTG	4E
γPNA1/3-30-nt spacer inward Bottom	GATCCAGGGAGGTTCTCAATGCGGATGTGCTCA GTCAGAGCTCTGATGAGAACTGGCCCACCCTCG TGACCACCCG	
γPNA1_γPNA2 target_1-nt_ top	AATTGCCCACCCTCGTGACCACCCAGACCTACG GCGTGCAGTGCTGAGCTC	S1B
γPNA1_γPNA2 target _1nt_ bottom	GATCGAGCTCAGCACTGCACGCCGTAGGTCTGG GTGGTCACGAGGGTGGGC	
γPNA3_γPNA4 target_1-nt_top	AATTTCCGCATTGAGAACCTCCCTAAGTCACAA AATCTGGATATAGAGCTC	S1B
γPNA3_γPNA4 target_1-nt_bottom	GATCGAGCTCTATATCCAGATTTTGTGACTTAG GGAGGTTCTCAATGCGGA	
γPNA2_γPNA4 target_1-nt_top	AATTAGCACTGCACGCCGTAGGTCAAGTCACAA AATCTGGATATAGAGCTC	S1B
γPNA2_γPNA4 target_1-nt_bottom	GATCGAGCTCTATATCCAGATTTTGTGACTTGA CCTACGGCGTGCAGTGCT	
γtcPNA1 and γtcPNA2 main target TOP	AATTCCTTCTTCCTCGCGTCCTACAGGCGAAGA GGGAGGAGAGGAGCTC	S3C
γtcPNA1 and γtcPNA2 main target BOTTOM	GATCGAGCTCCTCTCCTCCCTCTTCGCCTGTAG GACGCGAGGAAGAAGG	

γPNA1 and γPNA3_both_1-nt mismatch 3' end top	AATTGCCCACCCTCGTGACCACCGATGGGAGGTTCTCAATGCGGAGAGCTC	S9B and S10B
γPNA1 and γPNA3_both_1-nt mismatch 3' end bottom	GATCGAGCTCTCCGCATTGAGAACCTCCCATCGGTGGTCACGAGGGTGGGC	
γPNA1 and γPNA3_both_2-nt mismatch 3' end top	AATTGCCCACCCTCGTGACCACGGATCGGAGGTTCTCAATGCGGAGAGCTC	S9B and S10B
γPNA1 and γPNA3_both_2-nt mismatch 3' end bottom	GATCGAGCTCTCCGCATTGAGAACCTCCGATCCGTGGTCACGAGGGTGGGC	
γPNA1 and γPNA3_both_3-nt mismatch 3' end top	AATTGCCCACCCTCGTGACCAGGGATCCGAGGTTCTCAATGCGGAGAGCTC	S9B and S10B
γPNA1 and γPNA3_both_3-nt mismatch 3' end bottom	GATCGAGCTCTCCGCATTGAGAACCTCGGATCCCTGGTCACGAGGGTGGGC	
γPNA1 and γPNA3_both_4-nt mismatch 3' end top	AATTGCCCACCCTCGTGACCTGGGATCCCAGGTTCTCAATGCGGAGAGCTC	S9B and S10B
γPNA1 and γPNA3_both_4-nt mismatch 3' end bottom	GATCGAGCTCTCCGCATTGAGAACCTGGGATCCCAGGTCACGAGGGTGGGC	
γPNA1 and γPNA3_both_1-nt mismatch-5' end top	AATTGGCCACCCTCGTGACCACCCAAGGGAGGTTCTCAATGCGGTGAGCTC	S9B and S10B
γPNA1 and γPNA3_both_1-nt mismatch-5' end bottom	GATCGAGCTCACCGCATTGAGAACCTCCCTTGGGTGGTCACGAGGGTGGCC	
γPNA1 and γPNA3_both_2-nt mismatch-5' end top	AATTGGGCACCCTCGTGACCACCCAAGGGAGGTTCTCAATGCGCTGAGCTC	S9B and S10B
γPNA1 and γPNA3_both_2-nt mismatch-5' end bottom	GATCGAGCTCAGCGCATTGAGAACCTCCCTTGGGTGGTCACGAGGGTGCCC	
γPNA1 and γPNA3_both_3-nt mismatch-5' end top	AATTGGGGACCCTCGTGACCACCCAAGGGAGGTTCTCAATGCCCTGAGCTC	S9B and S10B
γPNA1 and γPNA3_both_3-nt mismatch-5' end bottom	GATCGAGCTCAGGGCATTGAGAACCTCCCTTGGGTGGTCACGAGGGTCCCC	
γPNA1 and γPNA3_both_4-nt mismatch-5' end top	AATTGGGGTCCCTCGTGACCACCCAAGGGAGGTTCTCAATGGCCTGAGCTC	S9B and S10B
γPNA1 and γPNA3_both_4-nt mismatch-5' end bottom	GATCGAGCTCAGGCCATTGAGAACCTCCCTTGGGTGGTCACGAGGGACCCC	

γPNA1 and γPNA3_both_1-nt mismatch top-CENTRAL	AATTGCCCACCATCGTGACCACCCAAGGGAGGTTATCAATGCGGAGAGCTC	S9B and S10B
γPNA1 and γPNA3_both_1-nt mismatch bot-CENTRAL	GATCGAGCTCTCCGCATTGATAACCTCCCTTGGGTGGTCACGATGGTGGGC	
γPNA1 and γPNA3_both_2-nt mismatch top-CENTRAL	AATTGCCCACCAACGTGACCACCCAAGGGAGGTTAACAATGCGGAGAGCTC	S9B and S10B
γPNA1 and γPNA3_both_2-nt mismatch bot-CENTRAL	GATCGAGCTCTCCGCATTGTTAACCTCCCTTGGGTGGTCACGTTGGTGGGC	
γPNA1 and γPNA3_both_3-nt mismatch top-CENTRAL	AATTGCCCACCAAGGTGACCACCCAAGGGAGGTTAAGAATGCGGAGAGCTC	S9B and S10B
γPNA1 and γPNA3_both_3-nt mismatch bot-CENTRAL	GATCGAGCTCTCCGCATTCTTAACCTCCCTTGGGTGGTCACCTTGGTGGGC	
γPNA1 and γPNA3_both_4-nt mismatch top-CENTRAL	AATTGCCCACCAAGCTGACCACCCAAGGGAGGTTAAGTATGCGGAGAGCTC	S9B and S10B
γPNA1 and γPNA3_both_4-nt mismatch bot-CENTRAL	GATCGAGCTCTCCGCATACTTAACCTCCCTTGGGTGGTCAGCTTGGTGGGC	
γPNA1 and γPNA3_both_5-nt mismatch 3' end top	AATTGCCCACCCTCGTGACGTGGGATCCCTGGTTCTCAATGCGGAGAGCTC	S9B and S10B
γPNA1 and γPNA3_both_5-nt mismatch 3' end bottom	GATCGAGCTCTCCGCATTGAGAACCAGGGATCCCACGTCACGAGGGTGGGC	
γPNA1 and γPNA3_both_7-nt mismatch 3' end top	AATTGCCCACCCTCGTGTGGTGGGATCCCTCCTTCTCAATGCGGAGAGCTC	S9B and S10B
γPNA1 and γPNA3_both_7-nt mismatch 3' end bottom	GATCGAGCTCTCCGCATTGAGAAGGAGGGATCCCACCACACGAGGGTGGGC	
γPNA1 and γPNA3_both_9-nt mismatch 3' end top	AATTGCCCACCCTCGACTGGTGGGATCCCTCCAACTCAATGCGGAGAGCTC	S9B and S10B
γPNA1 and γPNA3_both_9-nt mismatch 3' end bottom	GATCGAGCTCTCCGCATTGAGTTGGAGGGATCCCACCAGTCGAGGGTGGGC	
γPNA1 and γPNA3_both_11-nt mismatch 3' end top	AATTGCCCACCCTGCACTGGTGGGATCCCTCCAAGACAATGCGGAGAGCTC	S9B and S10B
γPNA1 and γPNA3_both_11-nt mismatch 3' end bottom	GATCGAGCTCTCCGCATTGTCTTGGAGGGATCCCACCAGTGCAGGGTGGGC	

γPNA1 and γPNA3_both_5-nt mismatch-5' end top	AATTGGGGTGCCTCGTGACCACCCAAGGGAGGT TCTCAATCGCCTGAGCTC	S9B and S10B
γPNA1 and γPNA3_both_5-nt mismatch-5' end bottom	GATCGAGCTCAGGCGATTGAGAACCTCCCTTGG GTGGTCACGAGGCACCCC	
γPNA1 and γPNA3_both_7-nt mismatch-5' end top	AATTGGGGTGGGTCGTGACCACCCAAGGGAGGT TCTCATACGCCTGAGCTC	S9B and S10B
γPNA1 and γPNA3_both_7-nt mismatch-5' end bottom	GATCGAGCTCAGGCGTATGAGAACCTCCCTTGG GTGGTCACGACCCACCCC	
γPNA1 and γPNA3_both_9-nt mismatch-5' end top	AATTGGGGTGGGAGGTGACCACCCAAGGGAGGT TCTGTTACGCCTGAGCTC	S9B and S10B
γPNA1 and γPNA3_both_9-nt mismatch-5' end bottom	GATCGAGCTCAGGCGTAACAGAACCTCCCTTGG GTGGTCACCTCCCACCCC	
γPNA1 and γPNA3_both_11-nt mismatch-5' end top	AATTGGGGTGGGAGCAGACCACCCAAGGGAGGT TGAGTTACGCCTGAGCTC	S9B and S10B
γPNA1 and γPNA3_both_11-nt mismatch-5' end bottom	GATCGAGCTCAGGCGTAACTCAACCTCCCTTGG GTGGTCTGCTCCCACCCC	
γPNA1 and γPNA3_non-consecutive mis_2-nt top	AATTGCCCACCGTGGTGACCACCCAAGGGAGGT ACACAATGCGGAGAGCTC	S9B and S10B
γPNA1 and γPNA3_non-consecutive mis_2-nt bottom	GATCGAGCTCTCCGCATTGTGTACCTCCCTTGG GTGGTCACCACGGTGGGC	
γPNA1 and γPNA3_non-consecutive mis_4-nt top	AATTGCCCAGCGTGGAGACCACCCAAGGGAGCT ACACTATGCGGAGAGCTC	S9B and S10B
γPNA1 and γPNA3_non-consecutive mis_4-nt bottom	GATCGAGCTCTCCGCATAGTGTAGCTCCCTTGG GTGGTCTCCACGCTGGGC	
γPNA1 and γPNA3_non-consecutive mis_6-nt top	AATTGCCGAGCGTGGAGTCCACCCAAGGGTGCT ACACTAAGCGGAGAGCTC	S9B and S10B
γPNA1 and γPNA3_non-consecutive mis_6-nt bottom	GATCGAGCTCTCCGCTTAGTGTAGCACCCTTGG GTGGACTCCACGCTCGGC	
γPNA1 and γPNA3_non-consecutive mis_8-nt top	AATTGGCGAGCGTGGAGTCGACCCAAGCGTGCT ACACTAAGGGGAGAGCTC	S9B and S10B
γPNA1 and γPNA3_non-consecutive mis_8-nt bottom	GATCGAGCTCTCCCCTTAGTGTAGCACGCTTGG GTCGACTCCACGCTCGCC	
ssDNA target	AATTAGCACTGCACGCCGTAGGTCT GGGTGGTCACGAG**GGTGGGCATCC GCATTGAGAACCT**CCCTAAGTCAC AAAATCTGGATATAGTGAGCTC	S4

Supplementary Table S4. Primers used in this study

Primer number	Primer name	Sequence (5′ to 3′)	Used for
1442	RFP-Seq-F2	GGCCCCGTAATGCAGAAGAAG	Confirmation of cloning into pMRS vector, amplification of PNA invasion targets for mobility shift assays and determining the cleavage site by Sanger sequencing
1444	GFP-Seq-R2	CTCGGCGCGGGTCTTGTAG	Confirmation of cloning into pMRS vector and determining the cleavage site by Sanger sequencing
1447	pUC19-F	GGGCTGGCTTAACTATGCGG	Confirmation of cloning into pUC19 vector by Sanger sequencing
1448	pUC19-R	CGAGGAAGCGGAAGAGCG	Confirmation of cloning into pUC19 vector by Sanger sequencing
1557	GFP-Seq-R1	TCGCCCTTGCTCACTGGA	Amplification of PNA invasion targets for mobility shift assays

Supplementary Table S5. Guides used in this study

Name	Designation	Sequence (5′ to 3′)
CbAgo-γPNA-target 2	1500	/5PHOS/AGACCTACGGCGTGCAGTGC
CbAgo-γPNA-L-flank targeting bottom	1503	/5PHOS/TAAAACGACGGCCAGTGAATT
CbAgo-γPNA-R-flank targeting Top	1506	/5PHOS/TGCAGGTCGACTCTAGAGGAT
gDNA pMRS 13% GC targeting bottom	1531	/5PHOS/TATTAATAGTAATCAA
gDNA pMRS 13% GC targeting top	1532	/5PHOS/TTGATTACTATTAATA
gDNA pMRS 22% GC targeting bottom	1533	/5PHOS/TAATAGTAATCAATTACG
gDNA pMRS 22% GC targeting top	1534	/5PHOS/CGTAATTGATTACTATTA
gDNA pMRS 27% GC targeting bottom	1535	/5PHOS/AATAATGACGTATGT
gDNA pMRS 27% GC targeting top	1536	/5PHOS/ACATACGTCATTATT
gDNA pMRS 31% GC targeting bottom	1537	/5PHOS/TATTAGTCATCGCTAT
gDNA pMRS 31% GC targeting top	1538	/5PHOS/ATAGCGATGACTAATA
gDNA pMRS 35% GC targeting bottom	1539	/5PHOS/TTGTTTTGGCACCAAAA
gDNA pMRS 35% GC targeting top	1540	/5PHOS/TTTTGGTGCCAAAACAA
gDNA γPNA 1_invaded strand targeting_16 nt	1542	/5PHOS/ACCCTCGTGACCACCC
gDNA γPNA 2_invaded strand targeting_16nt	1543	/5PHOS/ACTGCACGCCGTAGGT
gDNA γPNA 3_16nt_1	1545	/5PHOS/AGGTTCTCAATGCGGA
gDNA γPNA 3_invaded strand targeting_16nt	1546	/5PHOS/TCCGCATTGAGAACCT
gDNA γPNA1_16nt	1561	/5PHOS/TGGTCACGAGGGTGGG
gDNA γPNA2_16nt_2	1563	/5PHOS/TACGGCGTGCAGTGCT
gDNA γPNA4_16nt	1564	/5PHOS/TCACAAAATCTGGATA
gRNA γPNA1_16nt	1622	/5PHOS/rUrGrGrUrCrArCrGrArGrGrGrUrGrGrG
gRNA γPNA3_16nt	1624	/5PHOS/rArGrGrUrUrCrUrCrArArUrGrCrGrGrA
gDNA Mis1	1669	/5PHOS/TGGTCACGATGGTGGG
gDNA Mis2	1670	/5PHOS/TGGTCACTTGGGTGGG
gDNA Mis3	1671	/5PHOS/TGGTCAGTTGGGTGGG
gDNA Mis4	1672	/5PHOS/TGGTCAGTTCGGTGGG
gDNA Mis5	1673	/5PHOS/TGGTCACTTGGGTGGG
gDNA Mis6	1674	/5PHOS/TGGTCAGTTGGGTGGG
gDNA Mis7	1675	/5PHOS/TGGTCAGTTCGGTGGG

gDNA Mis8	1676	/5PHOS/AGGTTATCAATGCGGA
gDNA Mis9	1677	/5PHOS/AGGTTAACAATGCGGA
gDNA Mis10	1678	/5PHOS/AGGTTAAGAATGCGGA
gDNA Mis11	1679	/5PHOS/AGGTTAAGTATGCGGA
gDNA Mis12	1680	/5PHOS/AGGTCACGAGGGTGGG
gDNA Mis13	1681	/5PHOS/TGGTCACGAGGGTGGC
gDNA Mis14	1682	/5PHOS/AGGTTCTCAATGCGGT
gDNA Mis15	1683	/5PHOS/TGGTCACGAGGGTGCC
gDNA Mis16	1684	/5PHOS/AGGTTCTCAATGCGCT
gDNA Mis17	1685	/5PHOS/TGGTCACGAGGGTCCC
gDNA Mis18	1686	/5PHOS/AGGTTCTCAATGCCCT
gDNA Mis19	1687	/5PHOS/TGGTCACGAGGGACCC
gDNA Mis20	1688	/5PHOS/AGGTTCTCAATGGCCT
gDNA γPNA 1_1-nt mismatch anchor	1714	/5PHOS/AGGTCACGAGGGTGGG
gDNA γPNA 1_1-nt mismatch seed	1715	/5PHOS/TGGACACGAGGGTGGG
gDNA γPNA 1_1-nt mismatch central	1716	/5PHOS/TGGTCACGTGGGTGGG
gDNA γPNA 1_1-nt mismatch supplementary	1717	/5PHOS/TGGTCACGAGGGTCGG
gDNA γPNA 1_1-nt mismatch tail	1718	/5PHOS/TGGTCACGAGGGTGGC
gDNA γPNA 1_2-nt mismatch seed	1719	/5PHOS/TGGAGACGAGGGTGGG
gDNA γPNA 1_2-nt mismatch central	1720	/5PHOS/TGGTCACGTCGGTGGG
gDNA γPNA 1_2-nt mismatch supplementary	1721	/5PHOS/TGGTCACGAGGGACGG
gDNA γPNA 3_1-nt mismatch anchor	1723	/5PHOS/TGGTTCTCAATGCGGA
gDNA γPNA 3_1-nt mismatch seed	1724	/5PHOS/AGGATCTCAATGCGGA
gDNA γPNA 3_1-nt mismatch central	1725	/5PHOS/AGGTTCTCTATGCGGA
gDNA γPNA 3_1-nt mismatch supplementary	1726	/5PHOS/AGGTTCTCAATGCTGA
gDNA γPNA 3_1-nt mismatch tail	1727	/5PHOS/AGGTTCTCAATGCGGT
gDNA γPNA 3_2-nt mismatch seed	1728	/5PHOS/AGGAACTCAATGCGGA
gDNA γPNA 3_2-nt mismatch central	1729	/5PHOS/AGGTTCTCTTTGCGGA
gDNA γPNA 3_2-nt mismatch supplementary	1730	/5PHOS/AGGTTCTCAATGATGA
gDNA γPNA1_16-nt_5'OH	1731	TGGTCACGAGGGTGGG
gDNA γPNA 3_16-nt_5'OH	1732	AGGTTCTCAATGCGGA
gDNA γPNA1_20-nt	1733	/5PHOS/GGGTGGTCACGAGGGTGGGC
gDNA γPNA1_18-nt	1734	/5PHOS/GTGGTCACGAGGGTGGGC

gDNA γPNA1_14-nt	1735	/5PHOS/TGGTCACGAGGGTG
gDNA γPNA1_12-nt	1736	/5PHOS/TGGTCACGAGGG
gDNA γPNA1_10-nt	1737	/5PHOS/TGGTCACGAG
gDNA γPNA 3_20-nt	1738	/5PHOS/AGGGAGGTTCTCAATGCGGA
gDNA γPNA 3_18-nt	1739	/5PHOS/GGAGGTTCTCAATGCGGA
gDNA γPNA 3_14-nt	1740	/5PHOS/AGGTTCTCAATGCG
gDNA γPNA 3_12-nt	1741	/5PHOS/AGGTTCTCAATG
gDNA γPNA 3_10-nt	1742	/5PHOS/AGGTTCTCAA
gDNA γPNA 5	1749	/5PHOS/TCCGAGCAGAAGAAGA
gDNA γPNA 6	1750	/5PHOS/TTGATGTGATGGGAGC
gDNA γPNA 3_1 PT_5'	1776	/5PHOS/A*GGTTCTCAATGCGGA
gDNA γPNA 3_2 PT_5'	1777	/5PHOS/A*G*GTTCTCAATGCGGA
gDNA γPNA 3_3 PT_5'	1778	/5PHOS/A*G*G*TTCTCAATGCGGA
gDNA γPNA 3_4 PT_5'	1779	/5PHOS/A*G*G*T*TCTCAATGCGGA
gDNA γPNA 3_5 PT_5'	1780	/5PHOS/A*G*G*T*T*CTCAATGCGGA
gDNA γPNA 3_1 PT_3'	1781	/5PHOS/AGGTTCTCAATGCGG*A
gDNA γPNA 3_2 PT_3'	1782	/5PHOS/AGGTTCTCAATGCG*G*A
gDNA γPNA 3_3 PT_3'	1783	/5PHOS/AGGTTCTCAATGC*G*G*A
gDNA γPNA 3_4 PT_3'	1784	/5PHOS/AGGTTCTCAATG*C*G*G*A
gDNA γPNA 3_5 PT_3'	1785	/5PHOS/AGGTTCTCAAT*G*C*G*G*A
γtcPNA guide 1	1867	/5PHOS/CGAAGAGGGAGGAGAG
γtcPNA guide 2	1869	/5PHOS/GACGCGAGGAAGAAGG
γPNA1 3′ end partial specific Guide	1871	/5PHOS/GGGTGGTCACGAGGGT
γPNA3 3′ end partial specific Guide	1872	/5PHOS/AGGGAGGTTCTCAATG
γPNA1 3′ end 1nt mismatch-specific guide	1873	/5PHOS/CGGTGGTCACGAGGGT
γPNA3 3′ end 1nt mismatch-specific guide	1874	/5PHOS/TGGGAGGTTCTCAATG
γPNA1 3′ end 2nt mismatch-specific guide	1875	/5PHOS/CCGTGGTCACGAGGGT
γPNA3 3′ end 2nt mismatch-specific guide	1876	/5PHOS/TCGGAGGTTCTCAATG
γPNA1 3′ end 3nt mismatch-specific guide	1877	/5PHOS/CCCTGGTCACGAGGGT
γPNA3 3′ end 3nt mismatch-specific guide	1878	/5PHOS/TCCGAGGTTCTCAATG
γPNA1 3′ end 4nt mismatch-specific guide	1879	/5PHOS/CCCAGGTCACGAGGGT
γPNA3 3′ end 4nt mismatch-specific guide	1880	/5PHOS/TCCCAGGTTCTCAATG
γPNA1 middle 2-nt mismatch-specific guide	1881	/5PHOS/TGGTCACGTTGGTGGG
γPNA1 middle 3-nt mismatch-specific guide	1882	/5PHOS/TGGTCACCTTGGTGGG
γPNA1 middle 4-nt mismatch-specific guide	1883	/5PHOS/TGGTCAGCTTGGTGGG

γPNA3 full PT gDNA	1884	/5PHOS/A*G*G*T*T*C*T*C*A*A*T*G*C*G*G*A
γPNA1 full PT gDNA	1885	/5PHOS/T*G*G*T*C*A*C*G*A*G*G*G*T*G*G*G
γPNA1 3′ end 5-nt mismatch-specific guide	1886	/5PHOS/CCCACGTCACGAGGGT
γPNA3 3′ end 5-nt mismatch-specific guide	1887	/5PHOS/TCCCTGGTTCTCAATG
γPNA1 3′ end 7-nt mismatch-specific guide	1888	/5PHOS/CCCACCACACGAGGGT
γPNA3 3′ end 7-nt mismatch-specific guide	1889	/5PHOS/TCCCTCCTTCTCAATG
γPNA1 3′ end 9-nt mismatch-specific guide	1890	/5PHOS/CCCACCAGTCGAGGGT
γPNA3 3′ end 9-nt mismatch-specific guide	1891	/5PHOS/TCCCTCCAACTCAATG
γPNA1 3′ end 11-nt mismatch-specific guide	1892	/5PHOS/CCCACCAGTGCAGGGT
γPNA3 3′ end 11-nt mismatch-specific guide	1893	/5PHOS/TCCCTCCAAGACAATG
γPNA1 5′ end 5-nt mismatch-specific guide	1894	/5PHOS/TGGTCACGAGGCACCC
γPNA3 5′ end 5-nt mismatch-specific guide	1895	/5PHOS/AGGTTCTCAATCGCCT
γPNA1 5′ end 7-nt mismatch-specific guide	1896	/5PHOS/TGGTCACGACCCACCC
γPNA3 5′ end 7-nt mismatch-specific guide	1897	/5PHOS/AGGTTCTCATACGCCT
γPNA1 5′ end 9-nt mismatch-specific guide	1898	/5PHOS/TGGTCACCTCCCACCC
γPNA3 5′ end 9-nt mismatch-specific guide	1899	/5PHOS/AGGTTCTGTTACGCCT
γPNA1 5′ end 11-nt mismatch-specific guide	1900	/5PHOS/TGGTCTGCTCCCACCC
γPNA3 5′ end 11-nt mismatch-specific guide	1901	/5PHOS/AGGTTGAGTTACGCCT
γPNA1 non-consecutive 2-nt mismatch specific guide	1902	/5PHOS/TGGTCACCACGGTGGG
γPNA3 non-consecutive 2-nt mismatch specific guide	1903	/5PHOS/GAGGTACACAATGCGG
γPNA1 non-consecutive 4-nt mismatch specific guide	1904	/5PHOS/TGGTCTCCACGCTGGG
γPNA3 non-consecutive 4-nt mismatch specific guide	1905	/5PHOS/GAGCTACACTATGCGG

γPNA1 non-consecutive 6-nt mismatch specific guide	1906	/5PHOS/TGGACTCCACGCTCGG
γPNA3 non-consecutive 6-nt mismatch specific guide	1907	/5PHOS/GTGCTACACTAAGCGG
γPNA1 non-consecutive 8-nt mismatch specific guide	1908	/5PHOS/TCGACTCCACGCTCGC
γPNA3 non-consecutive 8-nt mismatch specific guide	1909	/5PHOS/CGTGCTACACTAAGGG
gDNA γPNA 1_ 2-nt mismatch anchor	2120	/5PHOS/ACGTCACGAGGGTGGG
gDNA γPNA 1_ 2-nt mismatch tail	2121	/5PHOS/TGGTCACGAGGGTGCC
gDNA γPNA 3_ 2-nt mismatch anchor	2122	/5PHOS/TTGTTCTCAATGCGGA
gDNA γPNA 3_ 2-nt mismatch tail	2123	/5PHOS/AGGTTCTCAATGCGTT
gDNA γPNA 1_ 3-nt mismatch anchor	2124	/5PHOS/ACCTCACGAGGGTGGG
gDNA γPNA 1_ 3-nt mismatch seed	2125	/5PHOS/TGGAGTCGAGGGTGGG
gDNA γPNA 1_ 3-nt mismatch central	2126	/5PHOS/TGGTCACCTCGGTGGG
gDNA γPNA 1_ 3-nt mismatch supplementary	2127	/5PHOS/TGGTCACGAGCCAGGG
gDNA γPNA 1_ 3-nt mismatch tail	2128	/5PHOS/TGGTCACGAGGGTCCC
gDNA γPNA 3_ 3-nt mismatch anchor	2129	/5PHOS/TCCTTCTCAATGCGGA
gDNA γPNA 3_ 3-nt mismatch seed	2130	/5PHOS/AGGAAGTCAATGCGGA
gDNA γPNA 3_ 3-nt mismatch central	2131	/5PHOS/AGGTTCAGTATGCGGA
gDNA γPNA 3_ 3-nt mismatch supplementary	2132	/5PHOS/AGGTTCTCAAACGGGA
gDNA γPNA 3_ 3-nt mismatch tail	2133	/5PHOS/AGGTTCTCAATGCCCT
gDNA γPNA 1_ 4-nt mismatch anchor	2134	/5PHOS/ACCACACGAGGGTGGG
gDNA γPNA 1_ 4-nt mismatch seed	2135	/5PHOS/TGGAGTGGAGGGTGGG
gDNA γPNA 1_ 4-nt mismatch central	2136	/5PHOS/TGGTCACCTCCGTGGG
gDNA γPNA 1_ 4-nt mismatch supplementary	2137	/5PHOS/TGGTCACGACCCAGGG
gDNA γPNA 1_ 4-nt mismatch tail	2138	/5PHOS/TGGTCACGAGGGACCC
gDNA γPNA 3_ 4-nt mismatch anchor	2139	/5PHOS/TCCATCTCAATGCGGA

gDNA γPNA 3_ 4-nt mismatch seed	2140	/5PHOS/AGGAAGACAATGCGGA
gDNA γPNA 3_ 4-nt mismatch central	2141	/5PHOS/AGGTTCAGTTTGCGGA
gDNA γPNA 3_ 4-nt mismatch supplementary	2142	/5PHOS/AGGTTCTCATACGGGA
gDNA γPNA 3_ 4-nt mismatch tail	2143	/5PHOS/AGGTTCTCAATGGCCT
new 16-nt shorter guide 1	2144	/5PHOS/GGTCACGAGGGTGGGC
14-nt shorter guide 1	2145	/5PHOS/TCACGAGGGTGGGC
10-nt shorter guide 1	2146	/5PHOS/GAGGGTGGGC
new 16-nt shorter guide 2	2147	/5PHOS/AGGTTCTCAATGCGGA
14-nt shorter guide 2	2148	/5PHOS/GTTCTCAATGCGGA
10-nt shorter guide 2	2149	/5PHOS/TCAATGCGGA
γPNA1-Full complimentary guide 1	2155	/5PHOS/GGTCACGAGGGTGGGC
γPNA1-Flanking 1-nt guide 1	2156	/5PHOS/GTCACGAGGGTGGGCA
γPNA1-Flanking 2-nt guide 1	2157	/5PHOS/TCACGAGGGTGGGCAA
γPNA1-Flanking 3-nt guide 1	2158	/5PHOS/CACGAGGGTGGGCAAT
γPNA1-Flanking 4-nt guide 1	2159	/5PHOS/ACGAGGGTGGGCAATT
γPNA1-Flanking 5-nt guide 1	2160	/5PHOS/CGAGGGTGGGCAATTC
γPNA1-Flanking 6-nt guide 1	2161	/5PHOS/GAGGGTGGGCAATTCG
γPNA1-Flanking 7-nt guide 1	2162	/5PHOS/AGGGTGGGCAATTCGG
γPNA1-Flanking 8-nt guide 1	2163	/5PHOS/GGGTGGGCAATTCGGC
γPNA1-Flanking 9-nt guide 1	2164	/5PHOS/GGTGGGCAATTCGGCG
γPNA1-Flanking 10-nt guide 1	2165	/5PHOS/GTGGGCAATTCGGCGC
γPNA1-Flanking 11-nt guide 1	2166	/5PHOS/TGGGCAATTCGGCGCC
γPNA1-Flanking 12-nt guide 1	2167	/5PHOS/GGGCAATTCGGCGCCG
γPNA1-Flanking 13-nt guide 1	2168	/5PHOS/GGCAATTCGGCGCCGG
γPNA1-Flanking 14-nt guide 1	2169	/5PHOS/GCAATTCGGCGCCGGT
γPNA1-Flanking 15-nt guide 1	2170	/5PHOS/CAATTCGGCGCCGGTG
γPNA1-Flanking 16-nt guide 1	2171	/5PHOS/AATTCGGCGCCGGTGG
γPNA1-Flanking 17-nt guide 1	2172	/5PHOS/ATTCGGCGCCGGTGGA
γPNA1-Flanking 18-nt guide 1	2173	/5PHOS/TTCGGCGCCGGTGGAG
γPNA1-Flanking 19-nt guide 1	2174	/5PHOS/TCGGCGCCGGTGGAGT
γPNA1-Flanking 20-nt guide 1	2175	/5PHOS/CGGCGCCGGTGGAGTG
γPNA3-Full complimentary guide 2	2176	/5PHOS/AGGTTCTCAATGCGGA
γPNA3-Flanking 1-nt guide 2	2177	/5PHOS/GGTTCTCAATGCGGAG
γPNA3-Flanking 2-nt guide 2	2178	/5PHOS/GTTCTCAATGCGGAGA
γPNA3-Flanking 3-nt guide 2	2179	/5PHOS/TTCTCAATGCGGAGAG
γPNA3-Flanking 4-nt guide 2	2180	/5PHOS/TCTCAATGCGGAGAGC
γPNA3-Flanking 5-nt guide 2	2181	/5PHOS/CTCAATGCGGAGAGCT
γPNA3-Flanking 6-nt guide 2	2182	/5PHOS/TCAATGCGGAGAGCTC
γPNA3-Flanking 7-nt guide 2	2183	/5PHOS/CAATGCGGAGAGCTCG
γPNA3-Flanking 8-nt guide 2	2184	/5PHOS/AATGCGGAGAGCTCGA
γPNA3-Flanking 9-nt guide 2	2185	/5PHOS/ATGCGGAGAGCTCGAT

γPNA3-Flanking 10-nt guide 2	2186	/5PHOS/TGCGGAGAGCTCGATC
γPNA3-Flanking 11-nt guide 2	2187	/5PHOS/GCGGAGAGCTCGATCC
γPNA3-Flanking 12-nt guide 2	2188	/5PHOS/CGGAGAGCTCGATCCA
γPNA3-Flanking 13-nt guide 2	2189	/5PHOS/GGAGAGCTCGATCCAG
γPNA3-Flanking 14-nt guide 2	2190	/5PHOS/GAGAGCTCGATCCAGT
γPNA3-Flanking 15-nt guide 2	2191	/5PHOS/AGAGCTCGATCCAGTG
γPNA3-Flanking 16-nt guide 2	2192	/5PHOS/GAGCTCGATCCAGTGA
γPNA3-Flanking 17-nt guide 2	2193	/5PHOS/AGCTCGATCCAGTGAG
γPNA3-Flanking 18-nt guide 2	2194	/5PHOS/GCTCGATCCAGTGAGC
γPNA3-Flanking 19-nt guide 2	2195	/5PHOS/CTCGATCCAGTGAGCA
γPNA3-Flanking 20-nt guide 2	2196	/5PHOS/TCGATCCAGTGAGCAA
γPNA1 Full complimentary 3	2233	/5PHOS/GGGTGGTCACGAGGGT
γPNA1 inward 1-nt flanking	2234	/5PHOS/AGGGTGGTCACGAGGG
γPNA1 inward 2-nt flanking	2235	/5PHOS/CAGGGTGGTCACGAGG
γPNA1 inward 3-nt flanking	2236	/5PHOS/CCAGGGTGGTCACGAG
γPNA1 inward 4-nt flanking	2237	/5PHOS/TCCAGGGTGGTCACGA
γPNA1 inward 5-nt flanking	2238	/5PHOS/CTCCAGGGTGGTCACG
γPNA1 inward 6-nt flanking	2239	/5PHOS/GCTCCAGGGTGGTCAC
γPNA1 inward 7-nt flanking	2240	/5PHOS/AGCTCCAGGGTGGTCA
γPNA1 inward 8-nt flanking	2241	/5PHOS/GAGCTCCAGGGTGGTC
γPNA1 inward 9-nt flanking	2242	/5PHOS/AGAGCTCCAGGGTGGT
γPNA1 inward 10-nt flanking	2243	/5PHOS/CAGAGCTCCAGGGTGG
γPNA3 Full complimentary 4	2247	/5PHOS/AGGGAGGTTCTCAATG
γPNA3 inward 1-nt flanking	2248	/5PHOS/GAGGGAGGTTCTCAAT
γPNA3 inward 2-nt flanking	2249	/5PHOS/TGAGGGAGGTTCTCAA
γPNA3 inward 3-nt flanking	2250	/5PHOS/CTGAGGGAGGTTCTCA
γPNA3 inward 4-nt flanking	2251	/5PHOS/TCTGAGGGAGGTTCTC
γPNA3 inward 5-nt flanking	2252	/5PHOS/CTCTGAGGGAGGTTCT
γPNA3 inward 6-nt flanking	2253	/5PHOS/GCTCTGAGGGAGGTTC
γPNA3 inward 7-nt flanking	2254	/5PHOS/AGCTCTGAGGGAGGTTC
γPNA3 inward 8-nt flanking	2255	/5PHOS/GAGCTCTGAGGGAGGT
γPNA3 inward 9-nt flanking	2256	/5PHOS/GGAGCTCTGAGGGAGG
γPNA3 inward 10-nt flanking	2257	/5PHOS/TGGAGCTCTGAGGGAG

Supplementary methods

Argonaute *in vitro* cleavage assay with circular plasmid DNA

1) γPNA invasion into target dsDNA (linear or circular)

- Prepare 1 µM stock of γPNA and aliquot
- Recommended: prior to use heat the aliquots at 90°C for 20 minutes to reconstitute γPNA

Component	Stock concentration	Volume	Final concentration
H_2O	/	2	
MOPS buffer	10X	1	1X
γPNA 1	1 µM	1	100 nM
γPNA 3	1 µM	1	100 nM
Plasmid DNA	40 ng/µL (~ 3nM)	5	20 ng/µL (~ 1.5 nM)
		**10**	

Prepare at room temperature and incubate the reaction at 37°C for 45 minutes (for circular plasmids) or at 37°C overnight (for linear plasmids); lid temperature 39°C, no cooling step

2.) Guide loading

- Prepare 10 µM Argonaute (CbAgo or KmAgo) protein secondary stock from primary Argonaute protein stock using 1X pAgo reaction buffer
- Assemble each reaction for paired Agos in 2 half-reactions

Half reaction 1:

Component	Stock concentration	Volume	Final concentration
H_2O	/	12	
pAgo reaction buffer	10X	2	1X
Guide DNA1	10 µM	2	1 µM
Guide DNA2	10 µM	/	1 µM
Argonaute	10 µM	2	1 µM
		16	

Half reaction 2:

Component	Stock concentration	Volume	Final concentration
H_2O	/	10	
pAgo reaction buffer	10X	2	1X
Guide 1	10 μM	/	1 μM
Guide 2	10 μM	2	1 μM
Argonaute	10 μM	2	1 μM
		16	

- Incubate the reactions at 37°C for 15 minutes; lid temperature 39°C, no cooling step
- Combine 8 μL of half-reaction 1 with 8 uL of half-reaction 2 in one tube
- Add 4 uL of invaded plasmid (80 ng final) or 2 μL of non-invaded plasmid (from 40 ng/uL stock) and 2 uL H_2O
- Incubate the reaction at 37°C for 60 minutes; lid temperature 39°C
- In case of circular plasmid, add 2 uL of 10X CutSmart and 1 uL of appropriate restriction enzyme into the reaction and incubate at 37°C for 30 minutes. This step is not necessary for linearized targets.
- Add 1 μL of proteinase K (Invitrogen; catalog #: 25530049) and incubate at 37°C for 30 minutes
- Add 4 μL of 6X Gel Loading Dye, Purple (NEB; catalog #: B7024S) and load the sample onto 0.9% agarose gel with GelRed® and run the 200 mL gel for 1h and 30 minutes at 145 V.
